# Exploring the possibility of predicting human head hair greying from DNA using whole-exome and targeted NGS data

**DOI:** 10.1186/s12864-020-06926-y

**Published:** 2020-08-05

**Authors:** Ewelina Pośpiech, Magdalena Kukla-Bartoszek, Joanna Karłowska-Pik, Piotr Zieliński, Anna Woźniak, Michał Boroń, Michał Dąbrowski, Magdalena Zubańska, Agata Jarosz, Tomasz Grzybowski, Rafał Płoski, Magdalena Spólnicka, Wojciech Branicki

**Affiliations:** 1 Malopolska Centre of Biotechnology, Jagiellonian University, Kraków, Poland; 2 Faculty of Biochemistry, Biophysics and Biotechnology, Jagiellonian University, Kraków, Poland; 3 Faculty of Mathematics and Computer Science, Nicolaus Copernicus University, Toruń, Poland; 4 Institute of Environmental Sciences, Faculty of Biology, Jagiellonian University, Kraków, Poland; 5 Central Forensic Laboratory of the Police, Warsaw, Poland; 6 Laboratory of Bioinformatics, Nencki Institute of Experimental Biology, Warsaw, Poland; 7 Faculty of Law and Administration, Department of Criminology and Forensic Sciences, University of Warmia and Mazury in Olsztyn, Olsztyn, Poland; 8 Department of Forensic Medicine, Collegium Medicum of the Nicolaus Copernicus University, Bydgoszcz, Poland; 9 Department of Medical Genetics, Warsaw Medical University, Warsaw, Poland

**Keywords:** Head hair greying, Whole-exome sequencing, Targeted next-generation sequencing, Prediction modelling, *KIF1A*, *FGF5*

## Abstract

**Background:**

Greying of the hair is an obvious sign of human aging. In addition to age, sex- and ancestry-specific patterns of hair greying are also observed and the progression of greying may be affected by environmental factors. However, little is known about the genetic control of this process. This study aimed to assess the potential of genetic data to predict hair greying in a population of nearly 1000 individuals from Poland.

**Results:**

The study involved whole-exome sequencing followed by targeted analysis of 378 exome-wide and literature-based selected SNPs. For the selection of predictors, the minimum redundancy maximum relevance (mRMRe) method was used, and then two prediction models were developed. The models included age, sex and 13 unique SNPs. Two SNPs of the highest mRMRe score included whole-exome identified *KIF1A* rs59733750 and previously linked with hair loss *FGF5* rs7680591. The model for greying vs. no greying prediction achieved accuracy of cross-validated AUC = 0.873. In the 3-grade classification cross-validated AUC equalled 0.864 for no greying, 0.791 for mild greying and 0.875 for severe greying. Although these values present fairly accurate prediction, most of the prediction information was brought by age alone. Genetic variants explained < 10% of hair greying variation and the impact of particular SNPs on prediction accuracy was found to be small.

**Conclusions:**

The rate of changes in human progressive traits shows inter-individual variation, therefore they are perceived as biomarkers of the biological age of the organism. The knowledge on the mechanisms underlying phenotypic aging can be of special interest to the medicine, cosmetics industry and forensics. Our study improves the knowledge on the genetics underlying hair greying processes, presents prototype models for prediction and proves hair greying being genetically a very complex trait. Finally, we propose a four-step approach based on genetic and epigenetic data analysis allowing for i) sex determination; ii) genetic ancestry inference; iii) greying-associated SNPs assignment and iv) epigenetic age estimation, all needed for a final prediction of greying.

## Background

Aging is inherently connected with changes in human appearance. Progressive physical traits include skin aging signs, greying of hair and hair loss and are perceived as biomarkers of individual’s aging rate and general health [1–3]. Therefore, there is an increasing interest in understanding the mechanisms underlying the differences in the pace of changes in human appearance and thus the mechanisms of aging processes. This knowledge could find practical application in medicine by boosting the accuracy of assessment of the risk of age-related diseases and in cosmetic industry in order to develop products that could prevent or slow down the clinical signs of phenotypic aging [1, 4, 5]. The studies on age-related physical traits are also useful in forensics and anthropology. Genetic prediction of human appearance based on DNA left at the crime scene or extracted from human remains may speed up the process of human identification [6].

Hair greying is an age-dependent trait and is understood as a progressive loss of pigment from the growing hair shaft. According to the 50–50-50 rule about 50% of the population experiences about 50% of grey hair at the age of 50 years [7]. Under healthy aging conditions, the onset of hair greying in Europeans occurs at the age of ~ 35 years, while greying observed under the age of 30 years is usually termed as premature hair greying [8, 9]. However, the progression of hair greying varies between populations with Africans and Asians showing less grey hair with the onset of hair greying occurring ~ 10 years later when comparing to Europeans [10, 11]. Although the cause of hair greying has been extensively studied, it is still poorly understood, appears to be very complex and may involve many different mechanisms [5, 9]. These mechanisms include dysfunction of the follicular melanocytes and thus pigmentary machinery malfunction and defective self-maintenance of the melanocyte stem cells (MSCs) present in the hair bulge [8, 12]. MSCs are activated during hair regeneration, migrate out from the bulge to the hair matrix region and differentiate into pigment producing melanocytes. Therefore, numerous factors involved in a proper regulation of the MSCs maintenance can be involved in the development of hair greying. A factor that seems to contribute to all of the mechanisms leading to hair greying is oxidative stress [5, 9, 13]. Such oxidative toxicity in the hair follicle can be induced by both intrinsic (melanogenesis itself, genetics, hormones, active hair growth) and extrinsic (UV exposure, inflammation, drugs, smoking, obesity, emotional stress, poor nutrition) factors [5, 14–16].

Little is known about genetic predispositions to the development of early greying. Recent analyses conducted by Adhikari and colleagues have shown that hair greying exhibits only 27% heritability [17] in contrast to previous studies that pointed out the key contribution of genetic factors in hair greying susceptibility (90% heritability) [2, 16]. However, it has been revealed that different methods used for heritability measurement in genome-wide association studies (GWAS) studies have different accuracies. In particular, the method used by Adhikari et al. (REML implemented in GCTA method) has been shown to consistently underestimate the trait heritability level [18]. The abovementioned group has conducted GWAS on Latin Americans that revealed only one gene, *IRF4*, being significantly associated with hair greying [17]. *IRF4* has been previously associated with various appearance traits including hair colour, freckles and hair loss e.g. [19–22]. *IRF4* encodes interferon regulatory factor that interacts with the MITF transcription factor. MITF activates expression of the *TYR* gene encoding tyrosinase, a key enzyme in melanin synthesis [23]. Importantly, MITF has been also suggested to be engaged in MSCs maintenance through induction of the expression of the *BCL2* gene, a key factor in protection against oxidative stress in melanocytes [12, 24]. However, *IRF4* rs12203592 was found to explain only a small proportion of the total variation observed in head hair greying [17].

The goal of the current study was to assess the potential of genetic variants to predict head hair greying status in individuals of European descent. The study included a discovery stage which involved the exome-wide association analysis. Additionally, an expanded list of DNA variants previously associated with pigmentation, head hair shape/thickness and head hair loss was also evaluated for their association with head hair greying and their impact on prediction accuracy. Finally, the minimum redundancy maximum relevance (mRMRe) method was used for predictors selection and a set of carefully selected SNPs combined with the information on age and sex was next used to develop the prototype models for head hair greying prediction.

## Results

### Characteristics of the study population

**Fig. 1:**
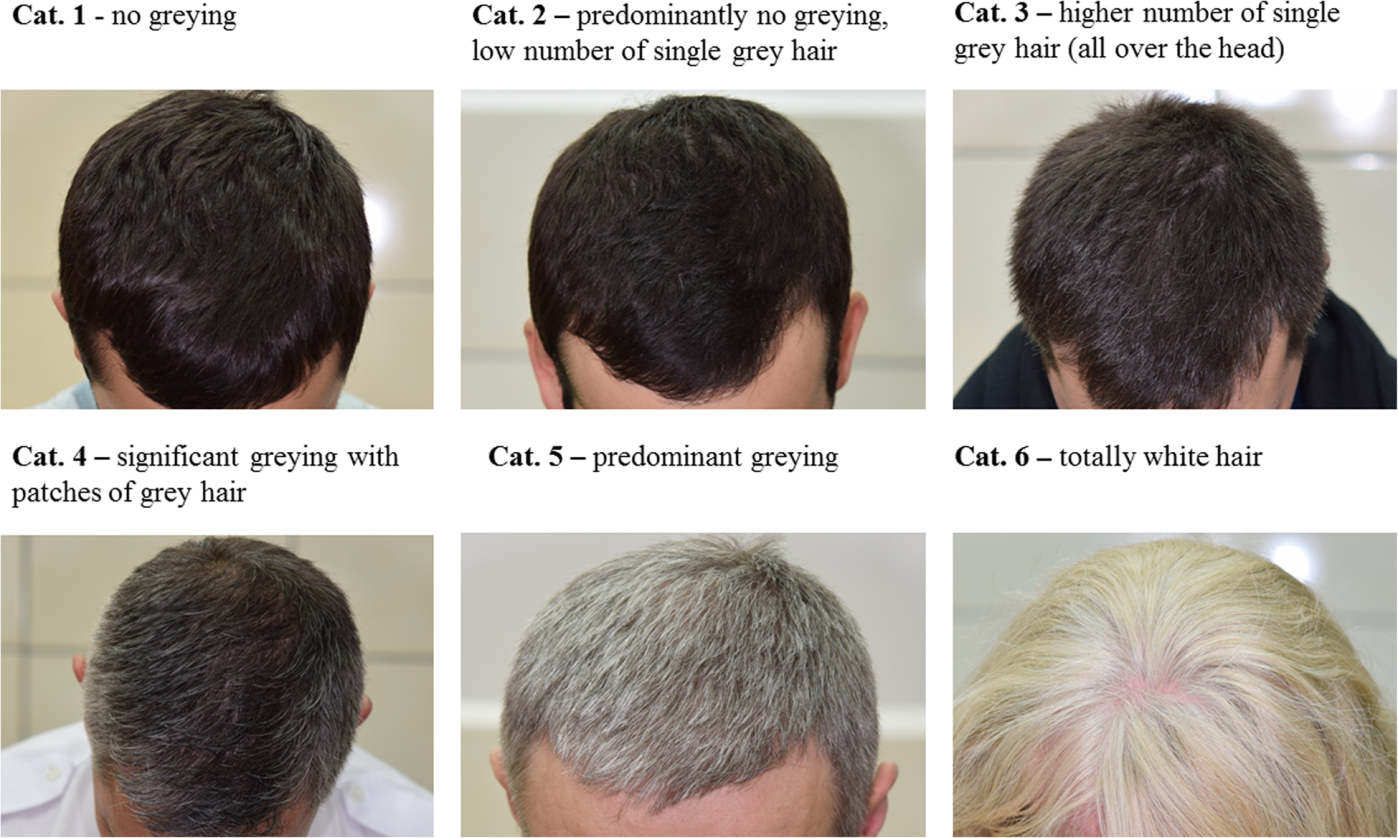
Hair greying 6-stage classification and examples

Hair greying was investigated in a set of 998 individuals from Poland aged ≥18 years. Study population included 673 males (67.4%) and 325 (32.6%) females. The mean age of the participants was 30.5 ± 8.8. Participants were assessed for grey hair using 6-stage classification (Fig. 1). For 26 samples only binary classification of hair greying was available (greying vs. no greying). Since information on the age of onset of hair greying was not available for all samples or was given only approximately, we did not use this information in the final statistical calculations. As expected, age was found to be significantly correlated with hair greying (Pearson’s *r* = 0.637, *P*-value = 2.183 × 10^− 114^). Hair greying was recorded in 14.3% of individuals aged 18–30 and the prevalence of grey hair was noted to be significantly higher in young males when comparing to young females (17.8 and 9.2%, respectively; χ^2^*P*-value = 0.004). The incidence of grey hair increased to 29.5% in the group of people aged 18–40 years and was 84.2% when people aged ≥40 years were considered. Analysis of head hair feature correlations showed significant and positive correlation of grey hair with dark hair colour (Pearson’s *r* = 0.152, *P*-value = 1.432 × 10^− 6^) and with hair loss in men (Pearson’s *r* = 0.297, *P*-value = 4.400 × 10^− 15^) but not with hair shape (Pearson’s *r* = − 0.044, *P*-value = 0.169). Straight hair was weakly and negatively correlated with grey hair but only when 6 hair greying categories were considered (Pearson’s *r* = − 0.084, *P*-value = 0.009).

A set of carefully selected (maintaining an appropriate age distribution and representativeness of particular hair greying categories) 149 samples was used as the discovery cohort for whole-exome sequencing (WES). The remaining 849 samples were used as a replication and prediction modelling cohort for the purpose of i) validation of DNA variants disclosed by WES; ii) validation of literature-based selected SNPs; iii) subsequent prediction model development. The characteristics of the sample cohorts under study are summarized in Supplementary Table 1.

### Exome-wide SNP association testing

WES enriched by regulome sequencing was conducted for 149 individuals with the defined degree of hair greying. After bioinformatic analysis and quality control filtering steps over 77 K common SNPs (MAF ≥ 5%) located within targeted regions and meeting the established criteria were extracted and subjected to exome-wide association testing. Regression analyses have revealed association with *P*-value < 5 × 10^− 4^ for 50 SNPs. LD-pruning (r^2^ ≤ 0.7) reduced the list of SNPs to 34 independent signals (Supplementary Tables 2–3). Two SNPs from this list showed nominal association (*P*-value < 0.05) in an independent cohort of 849 individuals (Table 1 and Supplementary Table 4). *KIF1A* rs59733750 (chr2:240780193) was found to be significantly associated with hair greying in all regression analyses with the highest significance achieved in multinomial ordinal logistic regression for 6 hair greying categories (MLR6) (*P*-value = 5.473 × 10^− 4^). *NSMCE1* rs1127228 (chr16:27226789) was found to be significantly associated with 3-stage and 6-stage hair greying classifications with the highest significance obtained in multinomial ordinal logistic regression for 3 hair greying categories (MLR3) (*P*-value = 0.015) (Table 1).

**Table 1:** Selection of exome-wide significant (*P*-value < 5 × 10^− 4^) and replicated (*P*-value < 0.05) SNPs associated with hair greying in a discovery and replication cohorts of 149 and 849 individuals from Poland, respectively

**SNP_ID**	**Chr position** **GRCh38**	**Gene**	**fMA**	**BLR**		**MLR3**		**MLR6**	
				**beta**	***P*-value**^**a**^	**beta**	***P*-value**^**a**^	**beta**	***P*-value**^**a**^
**Discovery cohort (*N*** **= 149);*P*-value < 5 × 10**^**− 4**^									
rs59733750	2:240780193	*KIF1A*	G 0.144	−1.782	0.013	−1.787	0.002	−1.861	**2.798 × 10**^**−4**^
rs1127228	16:27226789	*NSMCE1*	T 0.362	−1.360	0.005	−1.452	**4.312 × 10**^**−4**^	−1.404	**1.227 × 10**^**− 4**^
**Replication cohort (*N*** **= 849);*P*-value < 0.05**									
rs59733750	2:240780193	*KIF1A*	G 0.163	−0.484	**0.007**	−0.541	**0.002**	−0.569	**5.473 × 10**^**−4**^
rs1127228	16:27226789	*NSMCE1*	T 0.347	−0.241	0.079	−0.314	**0.015**	−0.283	**0.022**

BLR, binomial logistic regression; MLR3, multinomial ordinal logistic regression for 3 hair greying categories; MLR6, multinomial ordinal logistic regression for 6 hair greying categories; MA, minor allele; fMA, frequency of minor allele

^a^Results adjusted for age, sex and hair colour

### Association testing for literature-based selected candidate hair greying SNPs

**Fig. 2:**
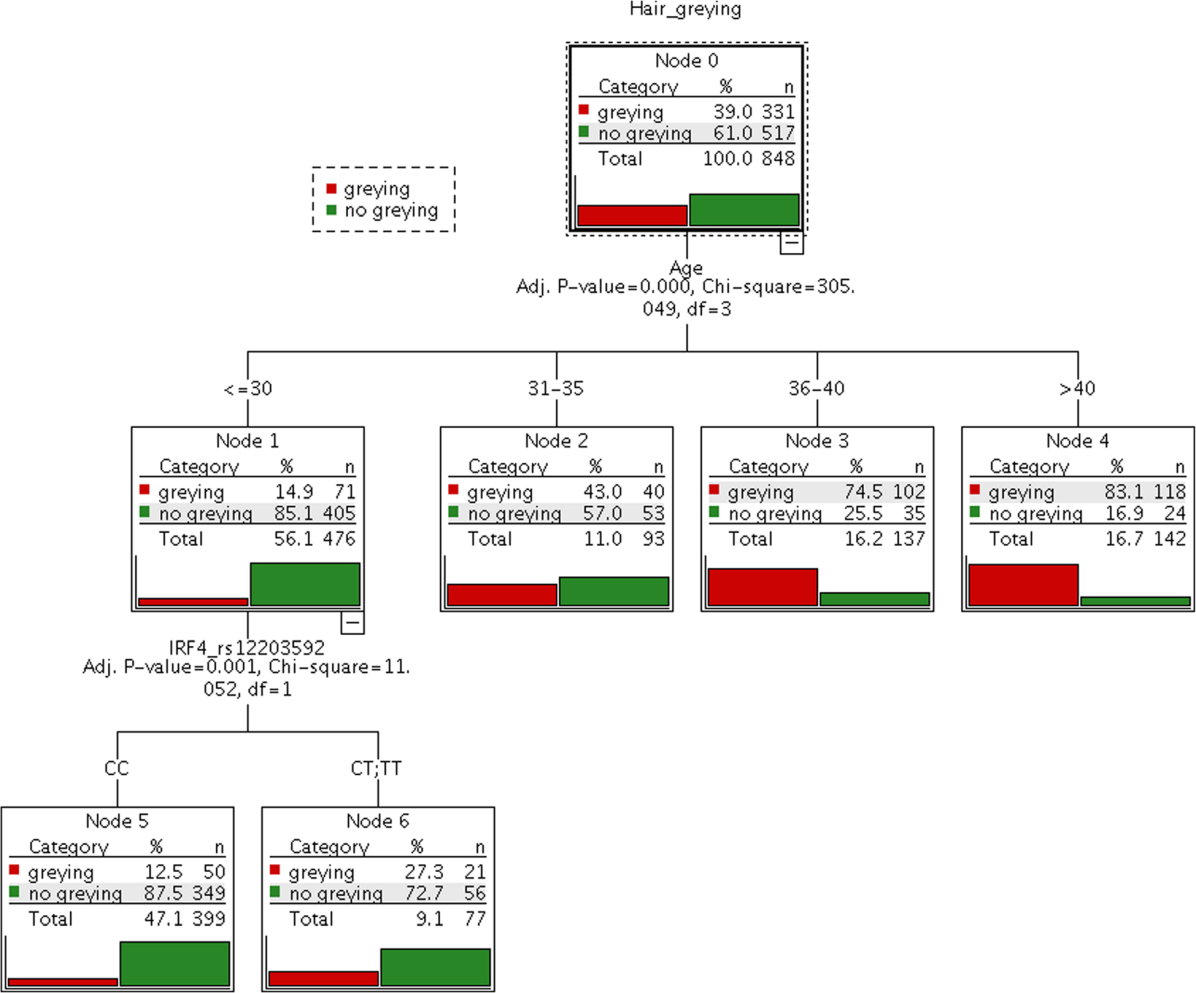
CHAID classification tree generated for greying vs. no greying classification in a replication cohort of 849 individuals from Poland using the data for age and *IRF4* rs12203592 only. The tree has 7 nodes with 5 terminal nodes. One sample was discarded from analysis due to the lack of information on age

*IRF4* rs12203592 (chr6:396321) was the only SNP discovered by Adhikari and colleagues of genome-wide significance in the only one genome-wide association study conducted so far for hair greying [17]. In the current study we replicated the effect of *IRF4* rs12203592 and showed it to be significantly associated with heir greying in a cohort of 849 samples (Table 2). In an univariate binomial regression analysis conducted for greying vs. no greying the minor T allele (fMA = 0.08) was found to increase the odds of grey hair by a factor of 2.0 (95% CI = 1.3–3.2, age and sex adjusted *P*-value = 0.003). From the Nagelkerke *R*^*2*^ statistic, this SNP explains 0.9% of the total variation observed in hair greying. To visualize the effect of *IRF4* rs12203592 on hair greying development CHAID tree analysis was conducted. As expected, age was at the top of the tree emphasizing its dominant role in hair greying formation (Fig. 2). Individuals ≤30 years old have only 14.9% probability of grey hair. *IRF4* rs12203592 T allele was found to impact grey hair occurrence in this youngest group of individuals increasing the probability of grey hair in CT/TT-rs12203592 individuals by ~ 15 p.p. (to a final probability of 27.3%) when comparing to CC-rs12203592 carriers.

**Table 2:** Validation of SNPs associated with hair greying by Adhikari et al. (2016) in a replication cohort of 849 individuals from Poland

**SNP_ID**	**Chr position** **GRCh38**	**Gene**	**fMA**	**BLR**		**MLR3**		**MLR6**	
				**beta**	***P*-value**^**a**^	**beta**	***P*-value**^**a**^	**beta**	*P*-value^*^
**rs12203592**	**chr6:396321**	***IRF4***	T 0.08	**0.700**	**0.003**	**0.669**	**0.002**	**0.780**	**1.121** × **10**^**−4**^
**rs2361506**	**chr2:233830694**	***MROH2A***	T 0.37	**0.360**	**0.008**	0.220	0.077	0.156	0.183
rs2085601	chr4:88974793	*FAM13A*	C 0.31	−0.068	0.630	−0.099	0.460	−0.056	0.658
rs7009516	chr8:24351334	*ADAM28*	G 0.46	0.036	0.785	−0.099	0.420	−0.107	0.361
rs1912702	chr11:79462038	*MIR708; TENM4*	T 0.37	0.029	0.823	−0.022	0.854	−0.012	0.917
rs11621135	chr14:71192892	*PCNX*; *LOC145474; SNORD56B*	A 0.44	0.028	0.829	0.065	0.588	0.031	0.786
rs281229	chr15:47426258	*SEMA6D*	T 0.00	-^b^	-^b^	-^b^	-^b^	-^**^	-^**^
rs1005241	chr22:47291868	*LOC101927722; TBC1D22A*	C 0.45	−0.153	0.252	−0.097	0.441	− 0.075	0.530

Significant results (*P*-value < 0.05) are marked with bold

BLR, binomial logistic regression; MLR3, multinomial ordinal logistic regression for 3 hair greying categories; MLR6, multinomial ordinal logistic regression for 6 hair greying categories; MA, minor allele; fMA, frequency of minor allele

^a^Results adjusted for age and sex

^b^Monomorphic SNP

Although *IRF4* rs12203592 was the only SNP that reached genome-wide significance in a study conducted by Adhikari et al., several suggestive associations have been also revealed for additional 7 loci. Besides *IRF4* rs12203592 we replicated the effect of *MROH2A* rs2361506 (chr2:233830694) (OR = 1.4, 95% CI = 1.1–1.9, age and sex adjusted *P*-value = 0.008) (Table 2). Age, *IRF4* rs12203592 and *MROH2A* rs2361506 altogether were found to explain 49.1% of variation observed in hair greying with 47.5% of variation explained by age and the remaining 1.6% explained by *IRF4* and *MROH2A*.

Because there are indications that the genetics underlying the various head characteristics may overlap to some extent, we evaluated the relationship to hair greying for additional list of 336 SNPs previously associated with hair colour / pigmentation, hair loss, shape and thickness. Out of these SNPs, positive signal of association, considering *P*-value < 0.05 as statistically significant and achieved in at least one of regression tests, was observed for 35 SNPs from 24 loci; *ERRFI1*/*SLC45A1*, *TCHH/RPTN*, *TEX41*, *HOXD-AS2/HOXD3*, LOC391485, *FGF5*, *EBF1*, *IRF4*, *SNX13, BRINP1,* 10p14*, GATA3, GRID1,* 11q24.2, *KRT71*, *P2RY5*, *OCA2*, *HERC2*, 16q24.1, *DPEP1*, *DEF8*, *APCDD1*, *PTK6* and *RUNX1*. Of the above, 8 loci (9 SNPs) were previously associated with hair loss, 8 loci (15 SNPs) were previously associated with pigmentation and the remaining 8 (11 SNPs) were linked to head hair shape (Supplementary Table 5). The highest significance (*P*-value < 0.01) was noted for *FGF5* rs7680591 (chr4:80276795) with *P*-value = 0.003 and P-value = 0.004 in MLR3 and MLR6 analyses, respectively and *DPEP1* rs164741 (chr16:89625890) with *P*-value = 0.004 and *P*-value = 0.007 in binomial logistic regression (BLR) and MLR3 analyses, respectively. These two SNPs were previously associated with hair loss and pigmentation, respectively, both explaining less than 1% of the total variation observed in hair greying in the studied population.

### Prediction modelling analyses

To increase the rate of success in selection of the most relevant set of SNP predictors we applied the mRMRe model selection method allowing simultaneous analysis of large sets of SNPs. SNPs were analysed in a 849-sample cohort, ranked according to the mRMRe score and the top 30 variants were extracted for both hair greying classifications (Supplementary Fig. 1 and Supplementary Table 6) and pruned based on analysis of scree plots (Supplementary Fig. 2). This analysis allowed pre-selection of 13 predictors for binomial hair greying classification (age + sex + 11 SNPs) and 16 for 3-stage classification (age + sex + 14 SNPs). Pre-selected variables were then assessed for their impact on prediction accuracy based on AUC values. This analysis led to a final list of 12 (age + sex + 10 SNPs) and 14 (age + sex + 12 SNPs) predictors included in the final binary neural network (BNN) and multi-class neural network (MNN) prediction models, respectively (Table 3). The workflow for the final selection of predictors is summarized on Supplementary Fig. 1. For both hair greying classifications, the highest mRMRe score was attributed to age. Age itself was found to explain Nagelkerke *R*^2^ 47.5% of the total variation observed in hair greying defined in a binary way (45.5% for hair 3-stage classification) and ensures the accuracy of head hair greying prediction at the level of AUC = 0.863 for greying vs. no greying and AUC = 0.859 for no greying, AUC = 0.788 for mild greying, AUC = 0.892 for severe greying in 3-stage classification (Table 3). Sex was ranked at the third position in BNN and at the second position in MNN but in contrast to age, sex explains significantly smaller proportion of the total variance observed in hair greying (5.5% for BNN and 7.3% for MNN) and its impact on prediction accuracy was also found to be lower. Thirteen unique SNPs were included in BNN and/or MNN models with 9 SNPs overlapping between both sets of predictors. These 13 DNA variants included 4 exome-wide identified SNPs (*KIF1A* rs59733750, *NSMCE1* rs1127228, *SEMA4D* rs45483393, *TMEM132C* rs1683723), 3 SNPs associated with hair greying in Adhikari et al. (*IRF4* rs12203592, *MROH2A* rs2361506, *TBC1D22A* rs1005241), 5 SNPs previously associated with hair loss (*FGF5* rs7680591, *TEX41* rs10928235, *RUNX1* rs68088846, *BRINP1* rs2416699, *GRID1* rs2814331) and 1 SNP previously linked with pigmentation (*DPEP1* rs164741). Ten out of 13 SNPs were found to achieve nominally significant association in regression tests (Tables 1 and 2, Supplementary Table 5), the remaining 3 SNPs included two SNPs (*SEMA4D* rs45483393 and *TMEM132C* rs1683723) identified with exome sequencing but not replicated in a set of 849 samples and one SNP (*TBC1D22A* rs1005241) with suggestive association in Adhikari et al. [17]. Among all the SNPs included in the models the highest mRMRe score was attributed to exome-wide identified *KIF1* rs59733750 (2^nd^ place in BNN and 4^rd^ place in MNN) and previously linked with hair loss *FGF5* rs7680591 (3^rd^ place in MNN model and 6^th^ place in BNN). Genetic variants were found to explain only a very small percentage of total variance in head hair greying. Altogether, 10 SNPs from BNN model and 12 SNPs from MNN explains 7.3% (total variance explained by age + sex + SNPs = 52.7%) and 9% (total variance explained by age + sex + SNPs = 52.4%), respectively. Therefore, their impact on prediction accuracy was found to be small with AUC change < 0.02 for individual SNPs (Table 3). Final models built on 849-sample set and including information on age, sex and DNA variation were found to predict hair greying status with accuracy of AUC = 0.900 for greying vs. no greying (increase by 0.037 when comparing to age-based model) and AUC = 0.894 for no greying (increase by 0.035 comparing to age-based model), AUC = 0.836 for mild greying (increase by 0.048) and AUC = 0.904 (increase by 0.012) for severe greying in 3-stage classification.

**Table 3:** The list of predictors included in the final binary (BNN) and 3-stage hair greying classification (MNN) models

**BNN model (greying vs. no greying)**						**MNN model (no greying vs. mild greying vs. severe greying)**							
**Rank**	**SNP_ID**	**Chr position** **GRCh38**	**Gene/Locus**	**mRMRe Score**	**AUC**	**Rank**	**SNP_ID**	**Chr position** **GRCh38**	**Gene/Locus**	**mRMRe Score**	**AUC**		
											**No greying**	**Mild greying**	**Severe greying**
1	Age	–	*–*	0.2429273	0.863	1	Age	–	–	0.2555938	0.859	0.788	0.892
2	rs59733750	2:240780193	*KIF1A*	0.0026937	0.864	2	Sex	–	–	0.0071533	0.861	0.803	0.891
3	Sex	–	*–*	0.0026841	0.866	3	rs7680591	4:80276795	*FGF5*	0.0026000	0.864	0.8	0.893
4	rs68088846	21:34835870	*RUNX1*	0.0026483	0.869	4	rs59733750	2:240780193	*KIF1A*	0.0024376	0.867	0.802	0.901
5	rs1005241	22:47291868	*TBC1D22A*	0.0023004	0.869	5	rs10928235	2:144920547	*TEX41*	0.0024138	0.869	0.803	0.901
6	rs7680591	4:80276795	*FGF5*	0.0022084	0.878	6	rs68088846	21:34835870	*RUNX1*	0.0023223	0.867	0.804	0.899
7	rs2361506	2:233830694	*MROH2A*	0.0020803	0.878	7	rs2361506	2:233830694	*MROH2A*	0.0023167	0.87	0.808	0.897
8	rs12203592	6:396321	*IRF4*	0.0016857	0.880	8	rs45483393	9:89378809	*SEMA4D*	0.0020403	0.87	0.806	0.899
9	rs45483393	9:89378809	*SEMA4D*	0.0015962	0.887	9	rs12203592	6:396321	*IRF4*	0.0020377	0.875	0.81	0.901
10	rs2416699	9:119434462	*BRINP1*	0.0015622	0.886	10	rs1005241	22:47291868	*TBC1D22A*	0.0016952	0.875	0.804	0.890
11	rs164741	16:89625890	*DPEP1*	0.0014107	0.888	11	rs2416699	9:119434462	*BRINP1*	0.0016313	0.878	0.812	0.896
12	rs1683723	12:128415460	*TMEM132C*	0.0010814	0.900	12	rs2814331	10:86233584	*GRID1*	0.0009898	0.879	0.821	0.903
–	–	–	*–*	*–*	–	13	rs164741	16:89625890	*DPEP1*	0.0008983	0.881	0.819	0.899
–	–	–	*–*	*–*	–	14	rs1127228	16:27226789	*NSMCE1*	0.0005584	0.894	0.836	0.904

mRMRe score and the impact on prediction accuracy measured by AUC values in a 849-sample cohort was presented for particular predictors

The accuracy of prediction of the final BNN and MNN hair greying models was further assessed by 10-fold cross-validation (CV). The cross-validated AUC values obtained were 0.873 for BNN and 0.864, 0.791 and 0.875 for no greying, mild greying and severe greying categories in MNN, respectively (Table 4). Sensitivity achieved for BNN equalled 0.734; out of 331 individuals with hair greying symptoms for 243 of them prediction was correct. Specificity was higher and reached 0.854; 442 individuals out of 518 individuals without any signs of hair greying were correctly assigned to no greying category. PPV value reached 0.762 which means that in 319 cases in which hair were classified as greying, for 243 of them prediction result was correct. NPV value at the level of 0.834 means that out of 530 no hair greying classifications in 83.4% of them prediction was correct and individuals indeed did not show any signs of hair greying. In case of MNN model, the highest sensitivity at the level of 0.886 was achieved for no greying category, the sensitivity for mild greying was 0.589 while the lowest value was gained for severe greying with only 7.7% of severe greying cases correctly predicted. However, for severe greying category the highest specificity was obtained at the level of 0.997. The specificity for no greying and mild greying equalled 0.643 and 0.821, respectively (Table 4).

**Table 4:** Final accuracy estimates of the BNN and MNN models for hair greying prediction designated in a 849-sample cohort using 10-fold cross-validation procedure

Model		AUC	Sensitivity	Specificity	PPV	NPV
**12-variable BNN**	**greying vs. no greying**	0.873	0.734 (243/331)	0.853 (442/518)	0.762 (243/319)	0.834 (442/530)
**14-variable MNN**	**no greying**	0.864	0.886 (459/518)	0.643 (196/305)	0.808 (459/568)	0.769 (196/255)
	**mild greying**	0.791	0.589 (149/253)	0.821 (468/570)	0.594 (149/251)	0.818 (468/572)
	**severe greying**	0.875	0.077 (4/52)	0.997 (769/771)	0.667 (2/6)	0.941 (769/817)

**Fig. 3:**
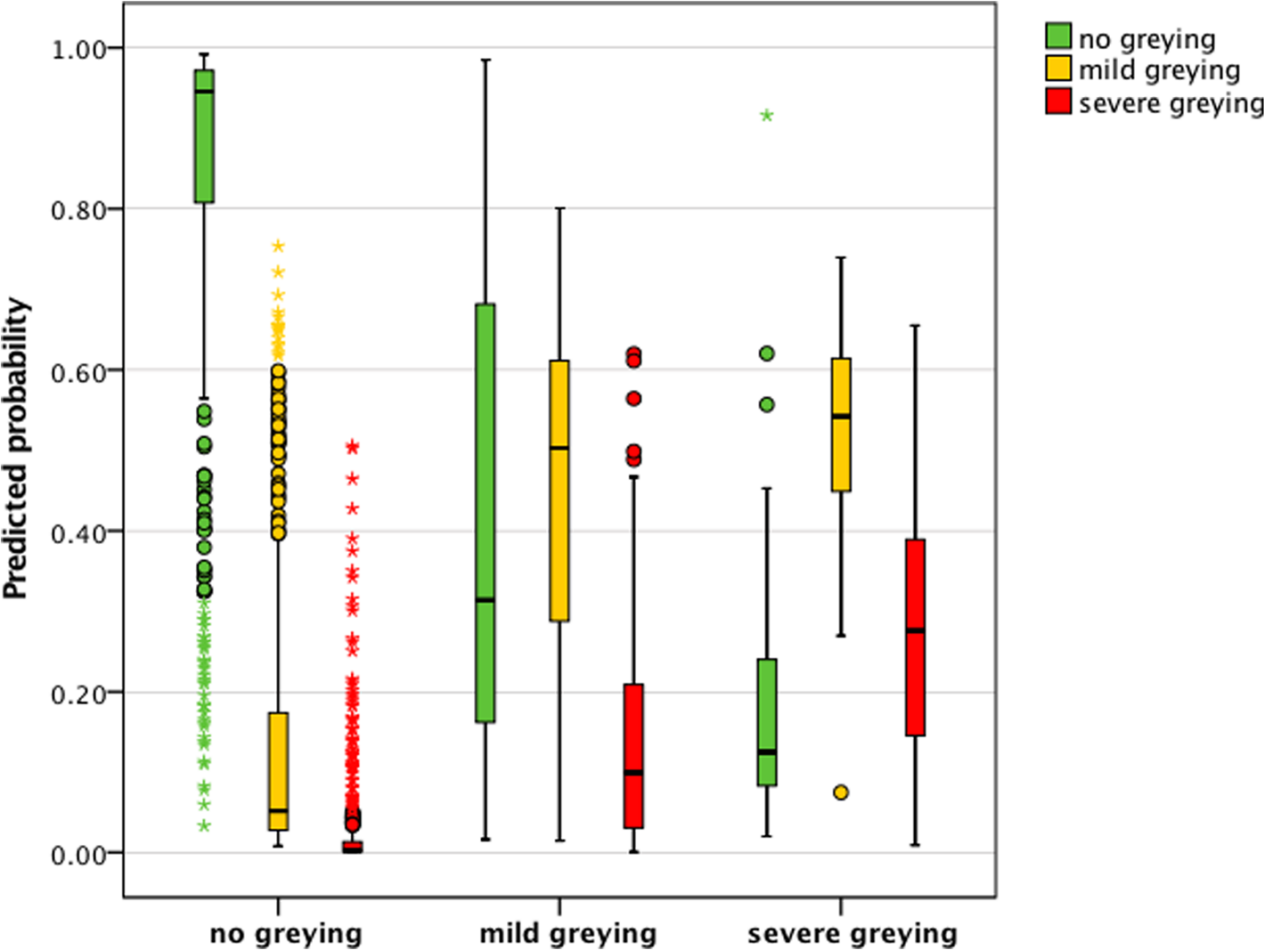
The distribution of hair greying predicted probabilities generated with MNN model

Low value of sensitivity observed for severe greying category means that most of the samples of severe greying (42/52) were classified as mild greying which indicates problems with differentiation between mild and severe greying categories. This is reflected by high probabilities for mild greying category generated for samples in severe greying category (Fig. 3). However, at the same time, an analysis of the distribution of probabilities generated with MNN model shows expected increase in the probability of severe greying when moving from no greying to severe greying category. The mean probability for severe greying category in mild greying category equalled 11.4 ± 10.5% while for individuals in severe greying category increased to 20.7 ± 13.6% (*P*-value = 9.62 × 10^− 8^).

The total number of correct predictions achieved with BNN model equalled 684/848 (80.7%) and was higher when comparing to the model built using information on age only (677/848) (Table 5). As the goal of the study was to evaluate if information on SNPs can improve accuracy of hair greying predictions, we focused on individuals i) young ((≤30 years old) and greying, *N* = 71 and ii) older (≥40 years old) and non-greying, *N* = 29 where age itself is non-informative (0% of correct predictions based on sole information from age, Table 5). This analysis showed that information on SNPs included in the models allowed proper recognition of 6 (8.5%) (BNN) or 3 (4.4%) (MNN) young and greying individuals and 2 (6.9%) old and no greying individuals when using MNN model.

**Table 5:** Success rate in prediction of hair greying status in a total 849-sample set and in two extreme phenotypic groups; i) ≤30 years old and greying, ii) ≥40 years old and no greying

Correct predictions	Total *N*	Young (≤30 y.o.) and greying	Old (≥40 y.o.) and no greying
**Age-based BNN model**	677/848^a^ (79.8%)	0/71 (0.0%)	0/29 (0.0%)
**12-variable BNN model**	684/848^a^ (80.7%)	6/71 (8.5%)	0/29 (0.0%)
**Age-based MNN model**	609/822^b^ (74.1%)	0/68 (0.0%)	0/29 (0.0%)
**14-variable MNN model**	611/822^b^ (74.3%)	3/68 (4.4%)	2/29 (6.9%)

^a^One sample was discarded from BNN analyses due to the lack of information on age

^b^One sample was discarded from MNN analyses due to the lack of information on age and 26 additional samples were omitted due to the availability of information on binary status of hair greying only

## Discussion

### The etiology of hair greying

Hair greying occurs as people age, but its progression is influenced by many different factors including sex, biogeographic ancestry, genetic predispositions and the impact of environment/lifestyle [5, 10, 16]. The average time before a first grey hair appears was estimated at age ~ 35 years in Europeans [11]. In our study ~ 14% of people at age below 30 years had grey hair and therefore were diagnosed with premature hair greying. The incidence of grey hair was found to be higher in males than females which is concordant with previous reports [11]. Furthermore, our study showed that dark-haired individuals harbor significantly more grey hair than the light-haired individuals which is in line with previous report [11]. Although the etiology of hair greying has been widely studied, it is still not fully understood. The image that arises from the current research indicates multifactorial background of hair greying with a wide range of different factors, different mechanisms and molecular pathways included [5, 9]. Hair greying comes along with many diseases including among others Waardenburg syndrome, spastic paraplegia, pernicious anemia and progeria (Hutchinson–Gilford and Werner syndrome) [25–28]. The genetic predisposition to the development of premature greying of hair has been recognized only to a very limited extent. Hair greying appears to be a polygenic trait, with a large number of genes involved with small or medium effect sizes. In a GWA study conducted in 2016 on a large sample of admixed Latin Americans, a single *IRF4* gene reached genome-wide significance. rs12203592 in *IRF4* was shown to explain merely ~ 8% of the total hair greying variation [17]. In the current research we have confirmed the effect of association of *IRF4* gene with hair greying in a Polish population and to the best of our knowledge this is the first study replicating the association disclosed by Adhikari et al. [17]. However, the proportion of the variation explained by *IRF4* rs12203592 in our study was found to be even smaller (~ 1%). *IRF4* rs12203592 was included in both models developed in our study but surprisingly was ranked low by mRMRe, at 8th and 9th positions in BNN and MNN models, respectively. Although *IRF4* rs12203592 was the only SNP that achieved GWA-significance, Adhikari et al. also reported suggestive associations for other 7 SNPs [17]. In univariate tests we replicated association of *MROH2A* rs2361506. This SNP was included in both models (ranked at 7^th^ position).

### Prediction modelling strategy

Although the human genome project provided impressive insights into the genetic architecture of many complex traits, large proportion of heritability of these phenotypes still remains unexplained [29]. Recent studies have demonstrated that accurate prediction of human complex traits may require a change in prediction modelling strategy and inclusion of large number of SNPs selected excluding the association criterion [30, 31]. This strategy gives the chance to reduce the problem of missing heritability that affects genetic prediction [29, 31, 32]. It has been shown that SNPs that show no signs of association in single tests can still improve prediction accuracy (e.g. [33]). Therefore, to increase the rate of success in identification of the relevant predictors for head hair greying: i) the significance threshold was lowered and considered *P*-value of 5 × 10^− 4^ as significant in exome-wide association study; ii) the list of candidate DNA variants was expanded by analysis of SNPs previously linked with different head hair features; iii) mRMRe method known to outperform classical approaches in terms of predictors selection was employed (mRMRe does not require SNPs to show significance in single tests). This approach allowed us to develop prototype models for head hair greying prediction that consider information on age, sex and DNA variation.

### Genetic component in the hair greying prediction models

The genetic component was found to explain merely < 10% of hair greying variation in the studied population which is substantially lower than the variation explained by age itself (> 45%). Consequently, the impact of particular SNPs on prediction accuracy was small. The list of genes implicated in hair greying phenotype include loci previously linked with smoking status (*KIF1A*, *RUNX1*, *IRF4*, *BRINP1*, *TEX41*, *GRID1*), BMI/obesity (*GRID1*, *TEX41*, *SEMA4D*, *TBC1D22A*, *RUNX1*), bone mineral density (*RUNX1*, *TBC1D22A, TEX41*), cardiovascular diseases (*FGF5*, *MROH2A*, *DPEP1*, *GRID1*) and immunology (*RUNX1*, *TBC1D22A, IRF4, TMEM132C, GRID1).* Importantly, all the above mentioned factors (particularly smoking) were linked with hair greying syndrome in previous research and therefore the link between these genes and greying seems to be justified [16, 33–36]. Three genes have been previously linked with ageing effects, that is longevity (*TBC1D22A*), skin aging (*IRF4*) and DNA methylation aging (*RUNX1*) [37–39]. Importantly, age-related changes in DNA methylation can stop melanocyte stem cells growth and thus potentially affect hair greying development [40]. It is also clear that variation in non-protein coding regions contributes to the phenotype. Of the 13 predictor SNPs in our final hair greying models, only two (rs1683723, rs1127228) affect protein sequence, whereas four (rs12203592, rs68088846, rs45483393, rs1127228) are located in known regulatory regions (enhancer, promoters, a CTCF binding site) [41].

### Novel genes associated with hair greying

Exome-wide association analysis and replication identified two novel DNA variants rs59733750 in *KIF1A* and rs1127228 in *NSMCE1* to be associated with hair greying. Of all SNPs included in the BNN model, rs59733750 in *KIF1A* received the highest mRMRe score (2nd position in the model). *KIF1A* (2q37.3) encodes a member of the kinesin family proteins that are responsible for the anterograde transport of synaptic-vesicle precursors along axons [42]. Mutations in *KIF1A* have been previously associated with disorders like spastic paraplegia 30 and hereditary neuropathy [42, 43]. Interestingly, specific types of spastic paraplegia disorders have been shown to be linked with pigmentary abnormalities, including premature hair greying and also associated with prematurely aged facial appearance [26, 44]. Noteworthy, neuropathy is the most common complication of diabetes which has been shown to alter expression of *KIF1A* and can lead to hair follicle damage [45–47]. It will be interesting to elucidate the role of *KIF1A* in molecular etiology behind hair greying. The analysis of genetic variation associated with various diseases can be controversial in forensic genetics for bioethical reasons. However, because pleiotropy is so common, it would be impossible to predict natural phenotypes avoiding genes involved in determination of pathological phenotypes. The penetrance of individual SNP variants is usually low and they altogether can only explain a small fraction of the predisposition to the disease [48] but the inclusion of disease-related genes in models for the genetic prediction of human appearance traits seems inevitable. This topic is currently widely discussed by forensic geneticists and bioethicians.

The second exome-wide identified locus, *NSMCE1* (16p12.1), was found to improve the accuracy of hair greying prediction in MNN model (ranked 14^th^). The NSMCE1 protein is a RING-type zinc finger-containing E3 ubiquitin ligase which belongs to the structural maintenance of chromosomes (SMC) proteins and is involved in the maintenance of genome integrity, DNA damage response and DNA repair [49]. Hair follicles are exposed to high levels of oxidative stress and the resulting DNA damage. Therefore, defects in DNA repair systems are considered as the important contributors to hair greying development [5, 9, 13]. The final models for hair greying included two additional exome-wide selected loci, *SEMA4D* rs45483393 and *TMEM132C* rs1683723 that were among the top mRMRe scored variables and were found to positively affect the accuracy of prediction even though these loci did not achieve statistical significance in single tests conducted in a replication cohort. *SEMA4D* (9q22.2) encodes semaphoring 4D protein that is a cell surface receptor for PLXNB1 and PLXNB2 and plays a role in axon guidance, immune response, tissue development, cell migration, cell-cell signaling and skin healing process [50]. It is noteworthy that rs281229 in *SEMA6D* (15q21.1), which is another member of the semaphoring family, has shown a suggestive association with hair greying in a study conducted by Adhikari et al. [17] but this DNA variant was monomorphic in our population. *TMEM132C* (12q24.32) encodes transmembrane protein 132C. Transmembrane proteins (TMEM) are components of various cell membranes and play important physiological functions although the biological meaning of particular proteins remains mostly unknown [51]. TMEM proteins were previously implicated in hair shape determination [52].

### Pleiotropic effects in head hair features

Aging of hair follicle is manifested by greying of the hair and hair loss, with both phenotypes being linked to each other [13, 17]. This correlation was also observed in our study. Out of 13 SNPs included in our predictive models for hair greying, 6 (*FGF5* rs7680591, *RUNX1* rs68088846, *IRF4* rs12203592, *BRINP1* rs2416699, *TEX41* rs10928235, *GRID1* rs2814331) were previously associated with male pattern baldness (MPB) [17, 21, 53, 54]. All 6 SNPs showed association with hair greying in our population. The highest significance was noted for *IRF4* rs12203592 and *FGF5* rs7680591. The *FGF5* gene (4q21.21) encodes Fibroblast Growth Factor 5. FGF proteins possess important functions in the regulation of cell growth and are engaged in a broad range of biological processes. FGF proteins have been suggested to be crucial regulators of hair growth [e.g. 15]. Mutations in *FGF5* have been linked with trichomegaly, a pathological condition involving abnormally long eyelashes [55]. The role of *FGF5* polymorphism in the development of MPB has recently been discovered in two large GWAS studies [21, 54]. Its importance for hair greying also seems possible because active hair growth, which leads to oxidative stress, has been proposed as one of the possible hypotheses for hair greying [15]. Indeed, gene expression of *FGF5* was downregulated in grey hair compared to black hair [15]. In our study, rs7680591 in *FGF5* was the highest-ranked DNA variant in the MNN model thus highlighting its role in hair greying prediction.

### Prediction of hair greying in practice

Prediction of hair greying status may have a practical value. In forensics, information about hair greying can be used for intelligence purposes [6]. As of yet, first predictive models for hair colour, hair loss and hair shape have been reported [56–60] but there are no studies examining the capabilities of head hair greying prediction. Although the prototype models that have been developed in the current research predicted hair greying status with very high accuracies of CV AUC = 0.873 for greying vs. no greying and CV AUC = 0.864 for no greying, CV AUC = 0.791 for mild greying and CV AUC = 0.875 for severe greying, the contribution of genetic predictor was very small. Due to the small effects sizes attributed to particular DNA variants only 2 or 3 hair greying categories were considered in the final statistical analyses. Larger studies should identify additional predictors that will allow better resolution in the future. It is worth noting that in forensics, the greatest practical significance will be predicting hair greying in extreme age categories. We have shown that the genetic component could correctly predict hair greying status in up to ~ 9% of individuals included in extreme phenotype groups. In addition to information about the age of a person, the prediction of hair greying also depends on additional factors, e.g. ancestry and environment. Recent years have proved that aging is associated with widespread changes in genome-wide DNA methylation [61, 62]. The observed differences in aging rates are in ∼30–40% heritable [63] but can be also influenced by the environment. It might be therefore anticipated that DNA methylation plays a mediating role between the environment and hair greying and that epigenetic age reflecting biological age of a person will be a significant predictor of grey hair, replacing chronological age in the models and at the same time accounting for environmental effects on hair greying. This hypothesis should be evaluated in the future.

**Fig. 4:**
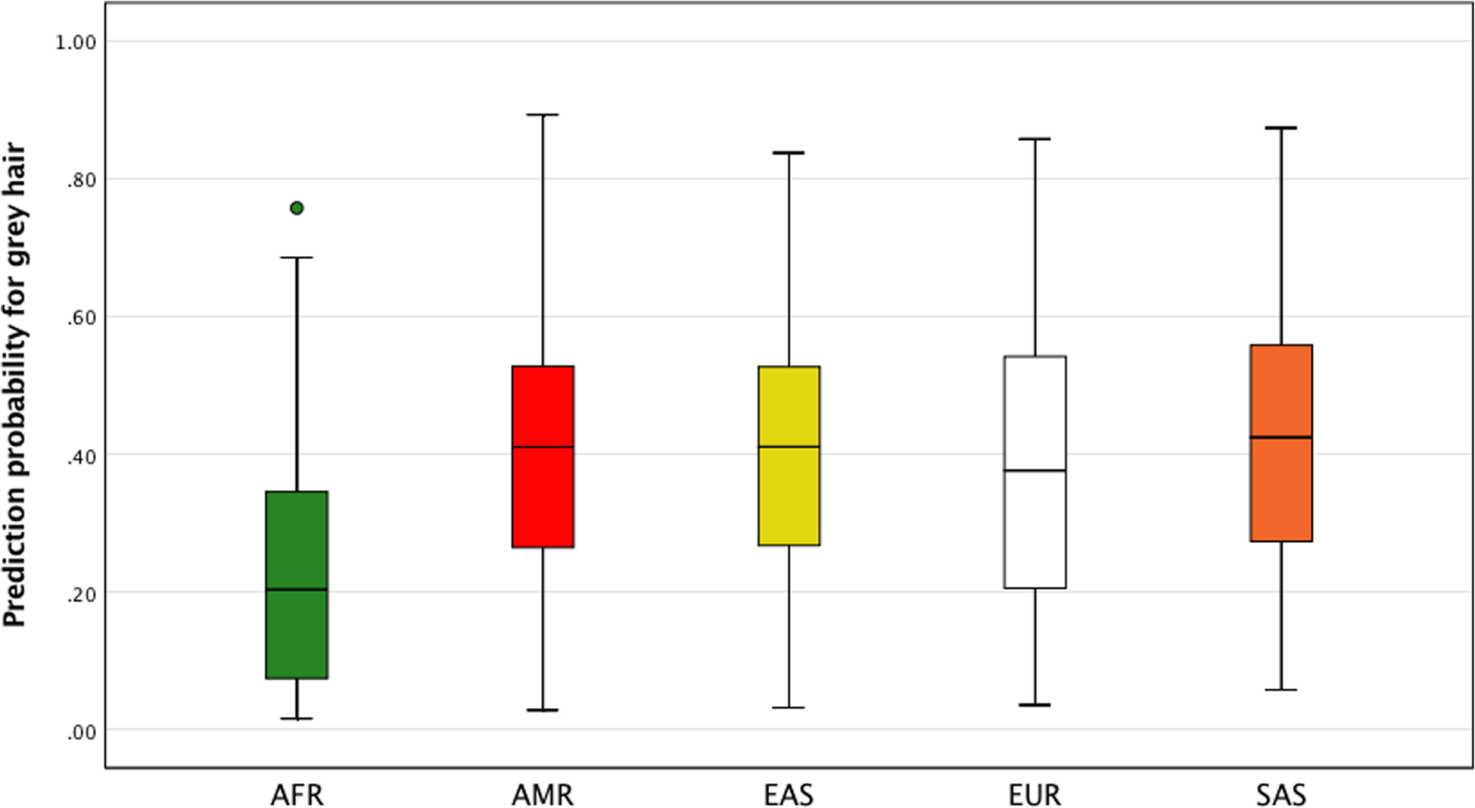
The distribution of the predicted grey hair probabilities in 2504 subjects from 19 worldwide countries extracted from The 1000 Genomes Project data. Prediction analysis was conducted using BNN model. Analysis included samples from Europeans (EUR), Africans (AFR), admixed Americans (AMR), South Asians (SAS), and East Asians (EAS)

Because our predictive models were developed using data for a Polish cohort, further research is needed to confirm their usefulness in other European and worldwide populations. The analysis of the 1000 Genomes data shows significant differences in minor allele frequencies for substantial number of SNPs (Supplementary Fig. 3 A-M). As both the age of onset and the rate of hair greying are linked to ancestry, with generally lower incidence of grey hair observed in African and Asian subjects [10, 11], it seems possible that genetic background will also differ to some degree. This is the case with hair loss for which inter-population differences are well described with the lowest incidence observed in Africans and later onset in Asians [57, 64]. To initially evaluate the performance of our SNPs in different populations we have applied our BNN model to 1000 Genome samples (extracted from ensembl.org) and analysed the distribution of hair greying probabilities. Although the phenotypes are not available for 1000 Genome samples we have generally observed significantly lower prediction probability values for grey hair in Africans (Fig. 4). The mean probability for grey hair in Europeans was estimated at 0.38 ± 0.19 while for Africans it amounted to 0.23 ± 0.17 (*P*-value = 5.092 × 10^− 40^). This outcome may result from substantial differences in allele frequencies between these two populations for a significant number of SNPs, including the highly rated in our models *FGF5* rs7680591 and *KIF1A* rs59733750 (Supplementary Fig. 3H and 3L).

**Fig. 5:**
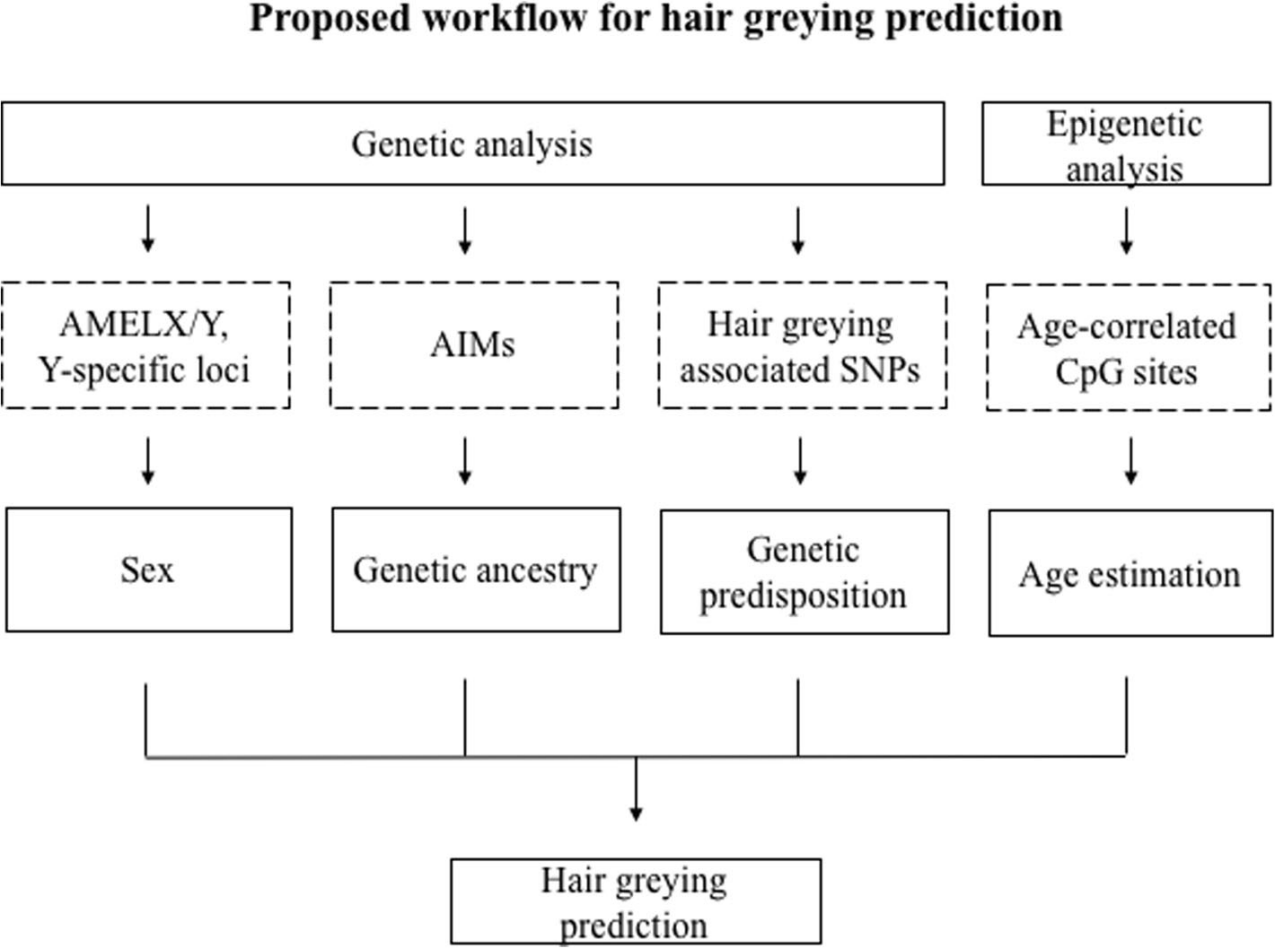
Proposed workflow for hair greying prediction based on genetic data and DNA methylation

Prediction of hair greying status solely based on genetic information is currently impossible and, as with other progressive human traits, should be accompanied by the estimation of a person’s age. It seems that a predictive algorithm based on genetic and epigenetic data may be practical in forensic investigations. Systematic approach that we propose should include: i) sex determination (typically done during standard STR profiling in forensic investigations via analysis of STR markers of Amelogenin gene on chromosomes X/Y); ii) genetic ancestry inference (through analysis of Ancestry Informative Markers (AIM-SNPs and/or AIM-Indels); iii) determination of genetic predispositions to develop hair greying through analysis of greying associated SNPs and iv) epigenetic age estimation (Fig. 5). Analogical solution was proposed to predict facial features. Information about genomic ancestry and sex was used to create a base-face which is next supplemented by genetic information on 24 facial variation associated SNPs [65]. Information on biogeographic ancestry (BGA) can still help predicting various appearance traits. This can complicate the implementation of predictive methods in forensics in those countries where DNA-based BGA inference is restricted by law [66], but cannot change until we fully understand the genetics of the traits, we do not identify all genes and functional variants and their interactions. In case of hair greying, there are clear indications in the literature that the age of onset and the rate of hair greying vary between populations. Future research will show if this is due to differences in allele frequency, different genetic basis and/or epistasis.

## Conclusions

To improve our understanding on the role of DNA variation in hair greying development we have conducted a novel study that enrolled 998 individuals from Poland that were carefully phenotyped for various head hair phenotypes. We have disclosed two novel DNA variants that were selected in a whole-exome analysis conducted in a discovery cohort and successfully validated in a replication cohort, namely *KIF1A* rs59733750 and *NSMCE1* rs1127228. Moreover, we have replicated the association of *IRF4* rs12203592 and *MROH2A* rs2361506 disclosed previously in a GWA study of Adhikari et al. (2016). We have also showed positive signal of association for 35 SNPs from 24 loci previously linked with pigmentation, hair loss and hair shape thus providing another evidence supporting hypothesis that the genetics underlying these characteristics is to some degree overlapping. The prototype models developed for 2- or 3-grade hair greying classification include information on age, sex and DNA variation within 13 unique SNPs, of which 3 variants showed no association in univariate regression tests. The developed models provided fairly accurate prediction of hair greying but most of the prediction information was fulfilled by age itself. DNA variants were found to explain < 10% of hair greying variation and have small impact on prediction parameters thus confirming hair greying as being genetically a very complex trait. Although our study is a step forward in better understanding of greying processes, further studies, including genetic and molecular analyses are certainly needed. More statistical power is needed to identify additional markers of hair greying and to facilitate prediction of this trait especially in extreme age groups. The role of DNA methylation aging in hair greying development and its impact on prediction should also be evaluated. Finally, we propose a complex predictor that should include sex determination, genomic ancestry inference, analysis of DNA variation and epigenetic age estimation for a final prediction of hair greying status. The validity of this approach should be evaluated in the future studies.

## Methods

### Sample collection and phenotypic classification

Blood samples were collected from 998 unrelated individuals from Poland at age ≥ 18 years (age range 19–62; Supplementary Table 1). Research participants were recruited at the Police Academy in Szczytno with financial support of the National Centre for Research and Development within the framework of the project NEXT (DOB-BIO7/17/01/2015). This research was approved by the Ethics Committee of the Jagiellonian University in Krakow (decision no. KBET/122/6120/11/2016). All participants provided written informed consent. With the help of the person conducting examination, participants completed a questionnaire including basic demographic data and phenotypic characteristics including hair greying status. Participants were asked to provide information on the occurrence of grey hair and the age at onset of hair greying. Additionally, high-quality photographs of the front part and the temporal part of the head were taken and evaluated for the progression of grey hair. 6-stage classification of grey hair has been applied as the adaptation of the 5-stage classification system used by Adhikari and co-workers [17]. The following categories were distinguished: 1 – no greying; 2 – predominantly no greying, low number of single grey hair; 3 - higher number of single grey hair (all over the head); 4 – significant greying with patches of grey hair; 5 – predominant greying; 6 – totally white hair (Fig. 1). For the purpose of statistical calculations, a simplified 3-stage classification was used, where categories 2 and 3 were merged and accounted for ‘mild greying’ while categories 4, 5 and 6 were combined into a category ‘severe greying’. Categories ‘mild greying’ and ‘severe greying’ were pooled for some analyses classifying hair greying as the binary outcome (greying vs. no greying). Participants were surveyed for hair dyeing, and if hair dyeing was found, detailed information on the condition and number of grey hair was collected.

### DNA extraction and quantification

Whole blood samples collected from all volunteers were subjected to DNA extraction using PrepFiler™ Forensic DNA Extraction Kit (ThermoFisher Scientific) according to the manufacturer’s protocol. DNA concentration was measured using Qubit dsDNA High-Sensitivity Assay Kit (Thermo Fisher Scientific) and Plexor® HY System (Promega).

### Whole-exome sequencing

WES analysis was conducted in a carefully selected cohort of 149 individuals maintaining adequate representativeness of particular phenotypic categories. Exonic regions (66 Mbp) have been enriched by regulatory sequences for > 160 loci previously associated with human appearance traits (including pigmentation, hair loss, hair shape/thickness) (1.5 Mbp) extracted from Nencki Genomics Database and FANTOM [67–69]. Libraries were prepared using SeqCap EZ MedExome Target Enrichment Kit (Roche NimbleGen, Wisconsin, USA). Sequencing was conducted on HiSeq1500 Illumina machine offering economical and high-throughput sequencing. HiSeq SBS v4 chemistry and paired-end reads protocol were applied (Illumina, San Diego, CA USA). Raw data generated with WES were subjected to the bioinformatic pipeline aimed at analysis of SNP variation. First, read quality control was performed with FastQC (http://www.bioinformatics.babraham.ac.uk/projects/fastqc). Next, reads were mapped to the GRCh38 human genome with Bowtie 2 [70]. To avoid allele amplification bias in variant calling the Picard MarkDuplicates command was used for duplicate reads removal (http://broadinstitute.github.io/picard/). To detect and correct systematic errors in base quality scores recalibration was performed with Genome Analysis Toolkit (GATK) BaseRecalibrator [71, 72]. Dbsnp138 available from GATK resource bundle was used as known sites database. Default parameter values were used. Regions which required realignment were selected by GATK RealignerTargetCreator, and local realignment was performed with GATK IndelRealigner [71, 72]. Variant calling restricted to the target region was performed with GATK Unified Genotyper. Filtering was performed with GATK VariantFiltration. To put the variant confidence QUAL score into perspective of the amount of coverage available variants with QD < 2.0 were filtered out. Also variants in which reads supporting the alternate allele had significantly lower mapping quality scores (MQRankSum < − 12.5) than those supporting the reference allele were filtered out. Genotypes with genotype quality < 20 and depth < 12 were treated as missing. For the downstream statistical analyses we have selected variants with less than 20% of missing data and global minor allele frequency (MAF) ≥ 5%.

### Candidate hair greying SNPs selection using exome-wide association testing

Exome-wide data (over 77 K SNPs) generated for 149 discovery samples was subjected to association testing to select candidate DNA variants for hair greying. As testing single variables is still the most common method in association studies (mainly due to computational burden) [73], such approach was also applied in the current study. Exome-wide association analyses were conducted using logistic regression methods for hair greying status defined as greying vs. no greying (binomial logistic regression; BLR) or using 3-stage or 6-stage classifications (multinomial ordinal logistic regression; MLR3 and MLR6). Analyses were conducted with R v3.5.2 using ‘ordinal’ package. Analyses were adjusted for age, sex and hair colour (dark vs. light) and additive allele effect was assumed. SNPs with *P*-values smaller than 5 × 10^− 4^ were considered as significant. Exome-wide association testing results were visualized using Manhattan and Q-Q graphs (Supplementary Figs. 4–5) plotted using R v3.5.2 and ‘qqman’ library. SNPs with *P*-values < 5 × 10^− 4^ were subjected to LD pruning which was conducted using PLINK 1.07. SNPs with independent effects (r^2^ ≤ 0.7) were retained in each region reducing the initial list of 50 candidate hair greying SNPs to 34 DNA variants.

### Candidate hair greying SNPs selection using literature data

It is anticipated that the genetics and biochemical pathways underlying different hair characteristics may be to some degree overlapping [17, 57, 58]. Adhikari et al. have shown correlations between different head hair features [17]. At least several genes (e.g. *EDAR*, *WNT10A*, *IRF4*, *SUCNR5*) have been proposed to influence more than one hair feature. Therefore, in the current study SNPs previously linked with hair loss, hair shape/thickness and pigmentation were evaluated for their association with hair greying. A detailed review of the literature allowed the selection of 344 SNPs (including 8 SNPs associated with hair greying, 90 SNPs associated with hair shape, 12 SNPs associated with hair thickness, 128 SNPs associated with hair loss (with 2 SNPs overlapping between hair shape and hair loss) and 108 SNPs associated with pigmentation) (Table 2 and Supplementary Table 5).

### Multiplex SNP genotyping using Ion Torrent S5

The final set of 378 candidate SNPs (including 34 WES-selected variants and 344 literature based-selected variants) was genotyped in a replication cohort of 849 Polish samples. Targeted NGS implemented in Ion AmpliSeq™ technology (ThermoFisher) which is offering efficient, sensitive and high-multiplexing solution was used to collect SNP genotypes. Data for the selected SNPs were generated within two Ion AmpliSeq™ panels covering a total of 828 SNPs, including 378 candidate SNPs for head hair greying and 450 candidate SNPs for other human appearance traits studied in the project NEXT. Primer pools were designed using an Ion AmpliSeq™ Designer tool (https://www.ampliseq.com) with a support of Thermo Fisher Scientific. Due to technical difficulties 3 SNPs: *TRPC3* rs1396082, *FBN3* rs56243829 and *DMKN* rs79338830 were replaced by SNPs in LD (*TRPC3* rs34306906, *FBN3* rs72993531 and *DMKN* rs77995042). DNA libraries were prepared using the Ion AmpliSeq™ Library Kit 2.0 according to the manufacturer’s protocol with a slight modification of PCR step (5–10 ng of DNA in 5 or 10 μL of total reaction volume). DNA libraries were quantified using Agilent High Sensitivity DNA Kit (Agilent Technologies, Santa Clara, USA) or Qubit dsDNA High-Sensitivity Assay Kit (Thermo Fisher Scientific), and then normalized to 40 pM. DNA libraries were combined in equal proportions and then template preparation was conducted using the Ion 520™ & Ion 530™ Kit-Chef and the Ion Chef System. Sequencing was performed with the Ion S5™ platform using Ion 530™ Chips. Raw data were analysed using Torrent Server and SNPs were called using Torrent Variant Caller v5.6.0.4 or alternatively HID SNP Genotyper plugin v5.2.2. Missing SNP-data were at 0.1%. The variable selection algorithms used in our research do not work on a set of data with missing values. Given the presence of missing data in neural network models, this would also result in poorer model quality. Therefore, the missing data were filled using ‘missForest’ method in R v3.5.2 (with a total number of trees equal to 500) and analyses were conducted on a more numerous data set.

### Association testing in a replication cohort

The whole set of genotyped SNPs was subjected to association testing in a replication cohort of 849 samples. Single-SNP association analyses were carried out with BLR and MLR3/MLR6 using libraries under R v3.5.2. All the results were adjusted for age and sex (in case of pigmentation-linked SNPs adjustment for hair colour was also applied) and additive allele effect was assumed. Results with *P*-values < 0.05 were considered as statistically significant. To visualize the effect of *IRF4* rs12203592 on hair greying development CHAID (Chi-squared Automatic Interaction Detection) analysis was conducted which is a classification method used to generate decision trees by using chi-square statistics. CHAID tree was generated with PS IMAGO PRO 5.0 (IBM SPSS Statistics 25) for a 849-sample cohort using the data for *IRF4* rs12203592 and age only.

### Pre-selection of SNPs for prediction model

Methods allowing simultaneous analysis of all the tested SNPs are assumed to outperform single SNP-tests in a selection of a final set of predictors [32]. As traditional methods like regression may not be efficient when working with a large number of SNPs we used mRMRe feature selection approach (https://www.rdocumentation.org/packages/mRMRe/versions/2.0.5) [74, 75]. mRMRe method is a fast framework for finding a set of the most relevant variables, based on a series of measures of relevance to the analysed trait and redundancy between the tested variables, thus outperforming classical approaches in terms of prediction accuracy [75]. mRMRe approach was applied to the genotypic data generated for all 378 candidate SNPs in a replication cohort, including information on age and sex. Analyses were conducted for hair greying defined in a binary way and using 3-stage classification. Classic mRMRe was applied and the top 30 variables in the output were generated. A set of 30 variables was analysed in terms of scoring and pruned based on analysis of scree plots (Supplementary Fig. 2). The resulting pre-selected set of predictors was further evaluated for the impact of particular markers on prediction accuracy measured by the area under the ROC curve (AUC) value as described in Section Prediction modelling using artificial NN and model validation*.*

### Prediction modelling using artificial NN and model validation

Prediction modelling was conducted using artificial neural network (NN) approach and a set of 849 samples. We decided not to divide samples into separate groups used for markers pre-selection and model building in order not to reduce the size of the samples. Thus, the selection of final markers was treated as a part of the model training, as in the wraper approach. Two prediction models were developed allowing prediction of hair greying at the binary level; greying vs. no greying (BNN; binary neural network) and assuming three states of hair greying; no greying, mild greying and severe greying (MNN; multi-class neural network). Multilayer perceptron with one hidden layer and an automatically selected number of neurons was used. Details of the parameters of the method used can be found in our previous study [76]. The impact of particular markers included in the mRMRe-based pre-selected set of predictors on prediction accuracy was evaluated by determining the value of the AUC parameter each time next variable with the highest mRMRe score was incorporated into the model. The analysis was stopped and the list of predictors was pruned after no clear increase in AUC value was observed (Supplementary Fig. 1). Prediction performance of the final models was verified using 10-fold cross-validation procedure , as described in detail in our previous work [76]. Then final prediction accuracy parameters including AUC, sensitivity, specificity, negative prediction value (NPV) and positive prediction value (PPV) [77] were calculated on the excluded *k*th parts of the data using the pooling strategy [78]. Analyses were carried out using PS IMAGO PRO 5.0 (IBM SPSS Statistics 25).

### Population analyses

Allele frequencies for the 13 SNPs included in the developed BNN and MNN models were plotted on the world map using allele frequency data extracted from “The 1000 Genomes Project” (https://www.ensembl.org/index.html) and using ArcMap 10.7 under ArcGIS Desktop software (Esri, Redlands, California). This data comes from 2504 subjects (19 worldwide countries) assigned to one of the following biogeographic ancestries: Europeans (EUR), Africans (AFR), admixed Americans (AMR), South Asians (SAS) and East Asians (EAS). In the next step the genotypes for these samples were extracted from http://grch37.ensembl.org/Homo_sapiens/Info/Index and analysed using BNN model. However, due to the lack of age and sex data for the samples, BNN model was rebuilt using DNA variants as the predictors only prior to analysis. Generated prediction probabilities for grey hair were then compared between populations.

## Supplementary information

**Additional file 1: Supplementary Fig. 1.** The applied workflow for the final selection of predictors for head hair greying. **Supplementary Fig. 2.** Scree plots generated for the top predictors selected with mRMRe method for A binary hair greying classification and B 3-stage hair greying classification. The mRMRe score values were plotted against their rank in the selected set of predictors. The suggestive cutoff point is indicated by the black horizontal line. In order to increase the transparency of the graphs and to improve determination of the cut-off point age (for binary hair greying classification) and age + sex (for 3-stage classification) were excluded when plotting graphs because of their significantly higher mRMRe scores. The order of the remaining predictors is in accordance with Supplementary Table 6. **Supplementary Fig. 3.** Global allele frequency distribution for 13 SNPs included in hair greying prediction models (A rs164741; B rs1005241; C rs1127228; D rs1683723; E rs2361506; F rs2416699; G rs2814331; H rs7680591; I rs10928235; J rs12203592; K rs45483393; L rs59733750; M rs68088846). Allele frequencies for the selected SNPs were plotted on the world map using data from “The 1000 Genomes Project” (http://grch37.ensembl.org/Homo_sapiens/Info/Index) and ArcMap 10.7 under ArcGIS Desktop software (Esri, Redlands, California). **Supplementary Fig. 4.** Manhattan plot of three EWAS analyses conducted in a 149 sample set for human head hair greying defined as A greying vs. no greying (BLR analysis); B no greying vs. mild greying vs. severe greying (MLR3 analysis); C 6-stage hair greying classification (MLR6 analysis). The -log10 (*P*-values) were plotted for each SNP under study according to its chromosomal position (GRCh38). The suggestive significance threshold (P-value = 5 × 10–4) is indicated as a black horizontal line and SNPs that reached the suggestive significance threshold are marked with green. **Supplementary Fig. 5.** Q-Q plots of three EWAS analyses conducted in a 149 sample set for human head hair greying defined as A greying vs. no greying (BLR analysis); B no greying vs. mild greying vs. severe greying (MLR3 analysis); C 6-stage hair greying classification (MLR6 analysis). **Supplementary Table 1.** Characteristics of the study population including discovery and replication/prediction modelling cohorts. **Supplementary Table 2.** LD analysis conducted for exome-wide selected SNPs. **Supplementary Table 3.** A selection of exome-wide identified SNPs (P-value < 5 × 10–4) associated with hair greying in a discovery cohort of 149 individuals from Poland. **Supplementary Table 4.** Replication analysis of EWAS results in a replication cohort of 849 Polish individuals. **Supplementary Table 5.** Results of association testing for 336 literature-based selected candidate SNPs for hair greying associated previously with hair colour/pigmentation, hair loss, hair shape and hair thickness. **Supplementary Table 6.** The results of mRMRe analysis for binary and 3-stage hair greying classification conducted in a 849-sample cohort

**Additional file 2: Supplementary Table 7.** The genotype-phenotype dataset generated with whole-exome sequencing.

**Additional file 3: Supplementary Table 8.** The genotype-phenotype dataset generated with targeted next-generation sequencing.

## Data Availability

The genotype-phenotype datasets generated with WES and targeted next-generation sequencing are included within the article as Supplementary Table 7 and Supplementary Table 8, respectively.

## Abbreviations

AFR: Africans
AMR: Admixed Americans
AIM: Ancestry informative markers
AUC: Area under the ROC curve
BGA: Biogeographic ancestry
BLR: Binomial logistic regression
BNN: Binary neural network
CHAID: Chi-squared Automatic Interaction Detection
CI: Confidence interval
CV: Cross-validation
DNA: Deoxyribonucleic acid
EAS: East Asians
EUR: Europeans
FGF: Fibroblast growth factor
GATK: Genome Analysis Toolkit
GWAS: Genome-wide association study
LD: Linkage disequilibrium
MAF: Minor allele frequency
MLR: Multinomial ordinal logistic regression
MNN: Multi-class neural network
MPB: Male pattern baldness
mRMRe: The minimum redundancy maximum relevance
MSCs: Melanocyte stem cells
NGS: Next-generation sequencing
NN: Neural network
NPV: Negative prediction value
PCR: Polymerase Chain Reaction
PPV: Positive prediction value
SAS: South Asians
SNP: Single nucleotide polymorphism
STR: Short tandem repeat
TMEM: Transmembrane proteins
WES: Whole-exome sequencing
