# Supplementary material for: Exploring the possibility of predicting human head hair greying from DNA using whole-exome and targeted NGS data
Source: BMC Genomics. 2020 Aug 5;21:538. doi: 10.1186/s12864-020-06926-y (PMC7430834; doi:10.1186/s12864-020-06926-y)
Supplement: Supplementary file 1 — Additional file 1: Supplementary Fig. 1. The applied workflow for the final selection of predictors for head hair greying. Supplementary Fig. 2. Scree plots generated for the top predictors selected with mRMRe method for A binary hair greying classification and B 3-stage hair greying classification. The mRMRe score values were plotted against their rank in the selected set of predictors. The suggestive cutoff point is indicated by the black horizontal line. In order to increase the transparency of the graphs and to improve determination of the cut-off point age (for binary hair greying classification) and age + sex (for 3-stage classification) were excluded when plotting graphs because of their significantly higher mRMRe scores. The order of the remaining predictors is in accordance with Supplementary Table 6. Supplementary Fig. 3. Global allele frequency distribution for 13 SNPs included in hair greying prediction models (A rs164741; B rs1005241; C rs1127228; D rs1683723; E rs2361506; F rs2416699; G rs2814331; H rs7680591; I rs10928235; J rs12203592; K rs45483393; L rs59733750; M rs68088846). Allele frequencies for the selected SNPs were plotted on the world map using data from “The 1000 Genomes Project” (http://grch37.ensembl.org/Homo_sapiens/Info/Index) and ArcMap 10.7 under ArcGIS Desktop software (Esri, Redlands, California). Supplementary Fig. 4. Manhattan plot of three EWAS analyses conducted in a 149 sample set for human head hair greying defined as A greying vs. no greying (BLR analysis); B no greying vs. mild greying vs. severe greying (MLR3 analysis); C 6-stage hair greying classification (MLR6 analysis). The -log10 (P-values) were plotted for each SNP under study according to its chromosomal position (GRCh38). The suggestive significance threshold (P-value = 5 × 10^–4^) is indicated as a black horizontal line and SNPs that reached the suggestive significance threshold are marked with green. Supplementary Fig. 5. Q-Q plots of three EWAS analyses conducted i [file 12864_2020_6926_MOESM1_ESM.pdf]

Supplementary Fig. 1 The applied workflow for the final selection of predictors for head hair greying

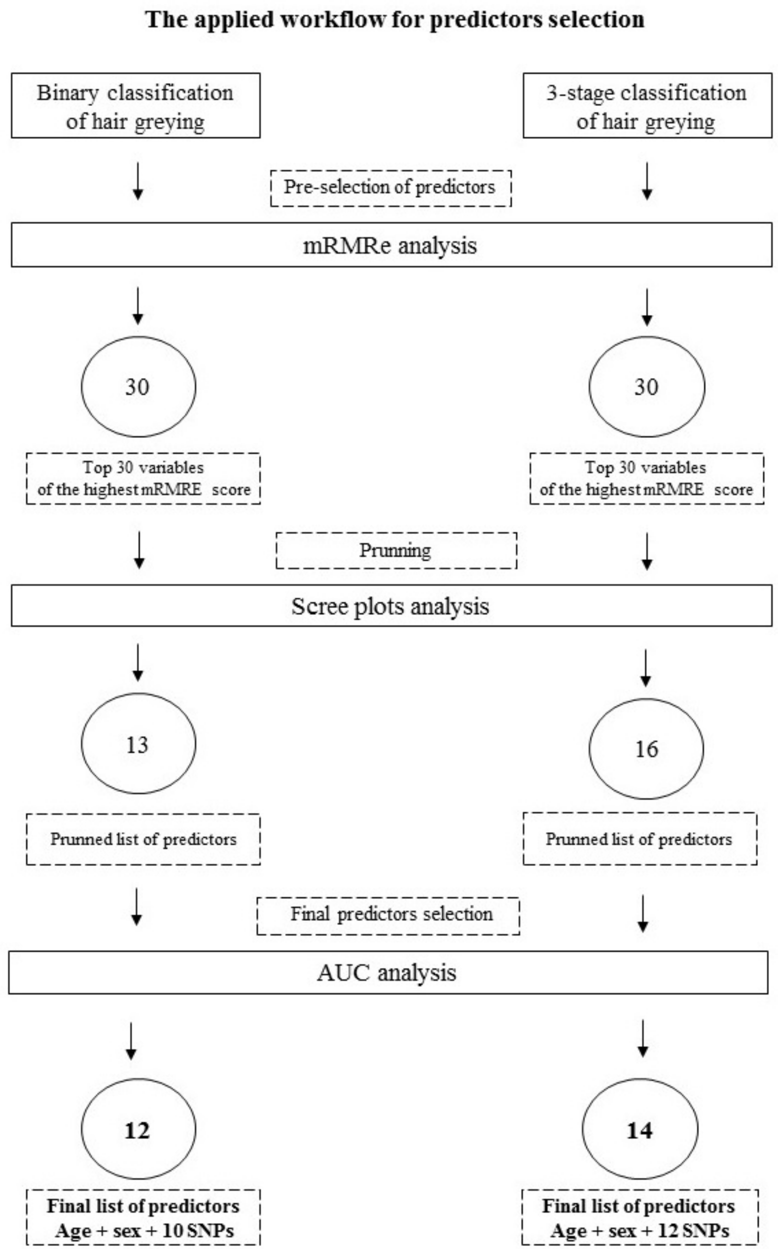

A

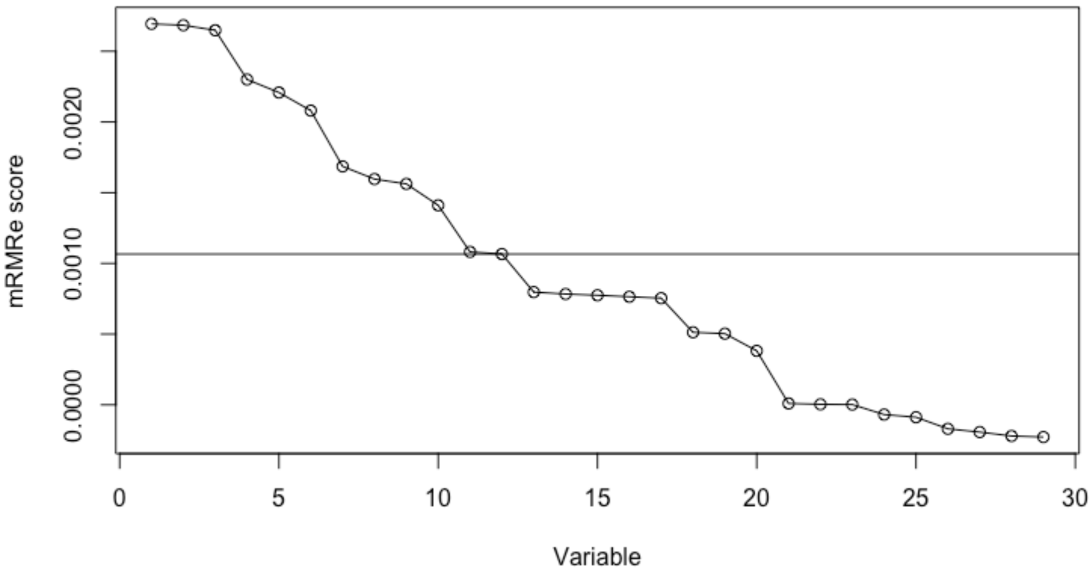

B

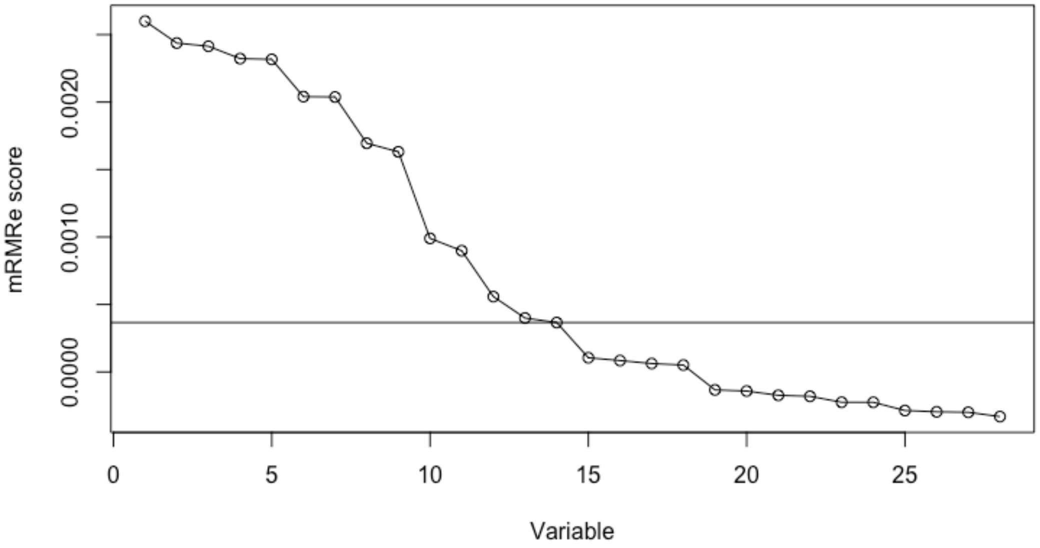

Supplementary Fig. 2 Scree plots generated for the top predictors selected with mRMRe method for **A** binary hair greying classification and **B** 3-stage hair greying classification. The mRMRe score values were plotted against their rank in the selected set of predictors. The suggestive cutoff point is indicated by the black horizontal line. In order to increase the transparency of the graphs and to improve determination of the cut-off point age (for binary hair greying classification) and age + sex (for 3-stage classification) were excluded when plotting graphs because of their significantly higher mRMRe scores. The order of the remaining predictors is in accordance with Supplementary Table 6.

A

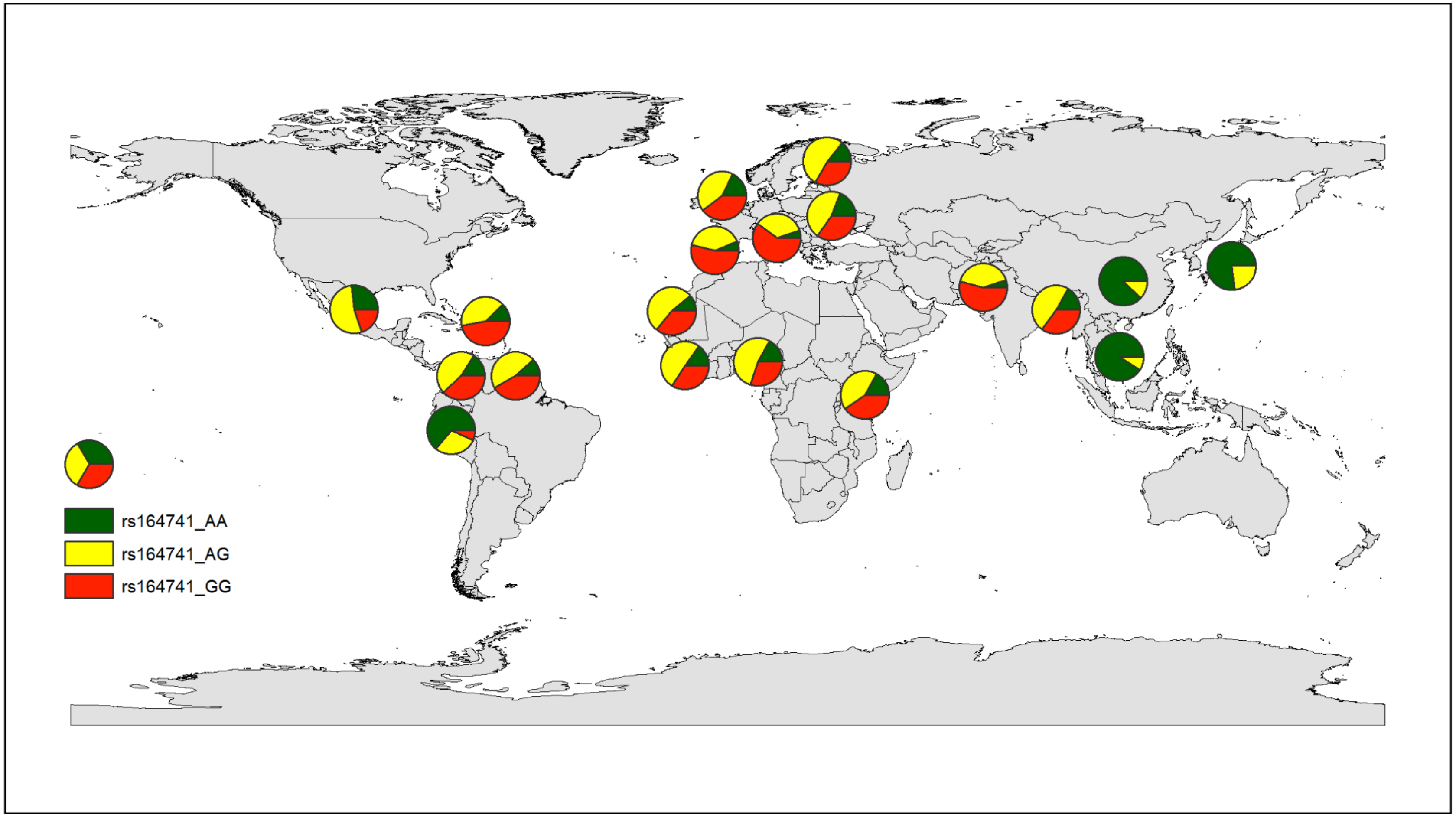

B

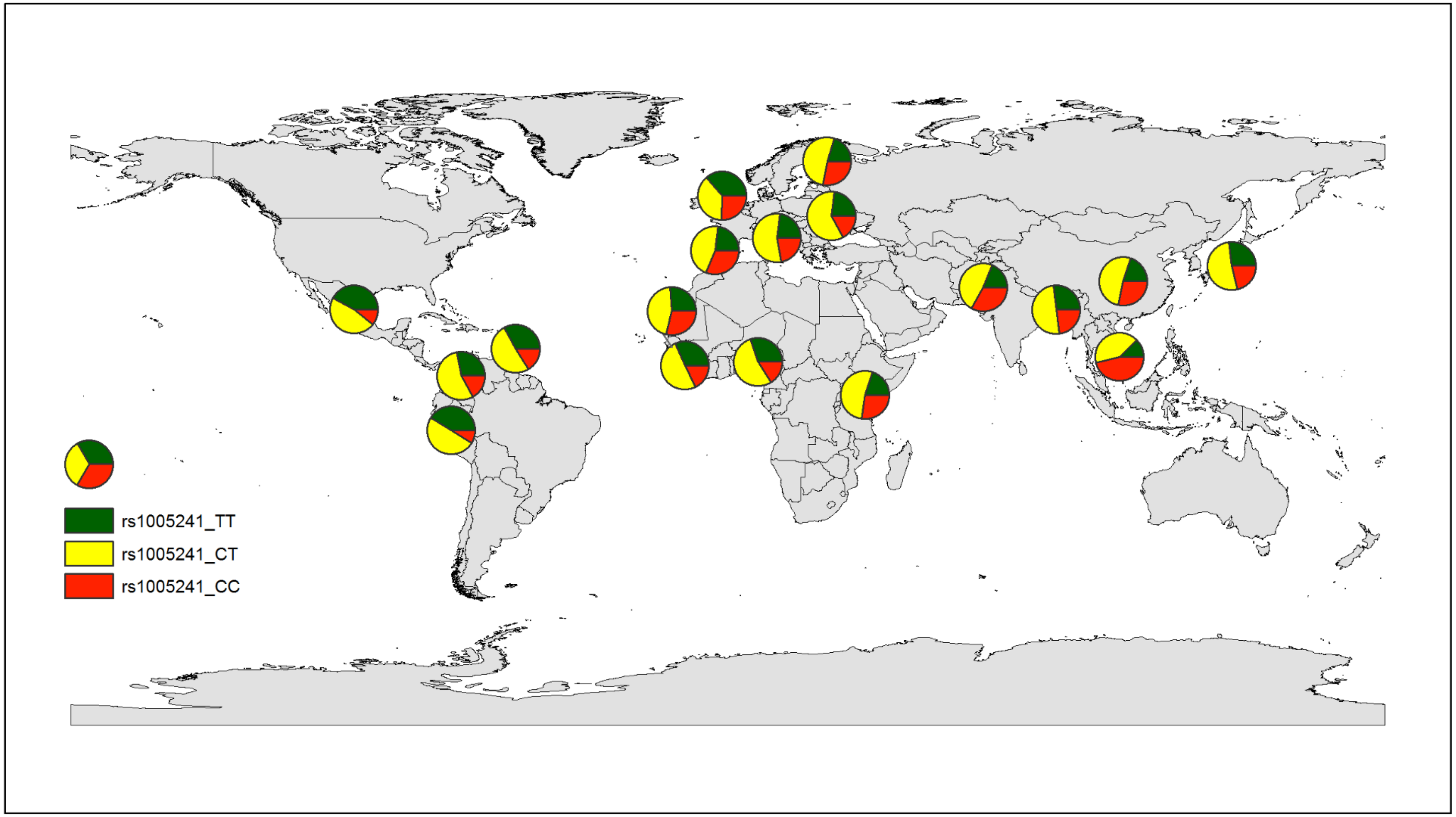

C

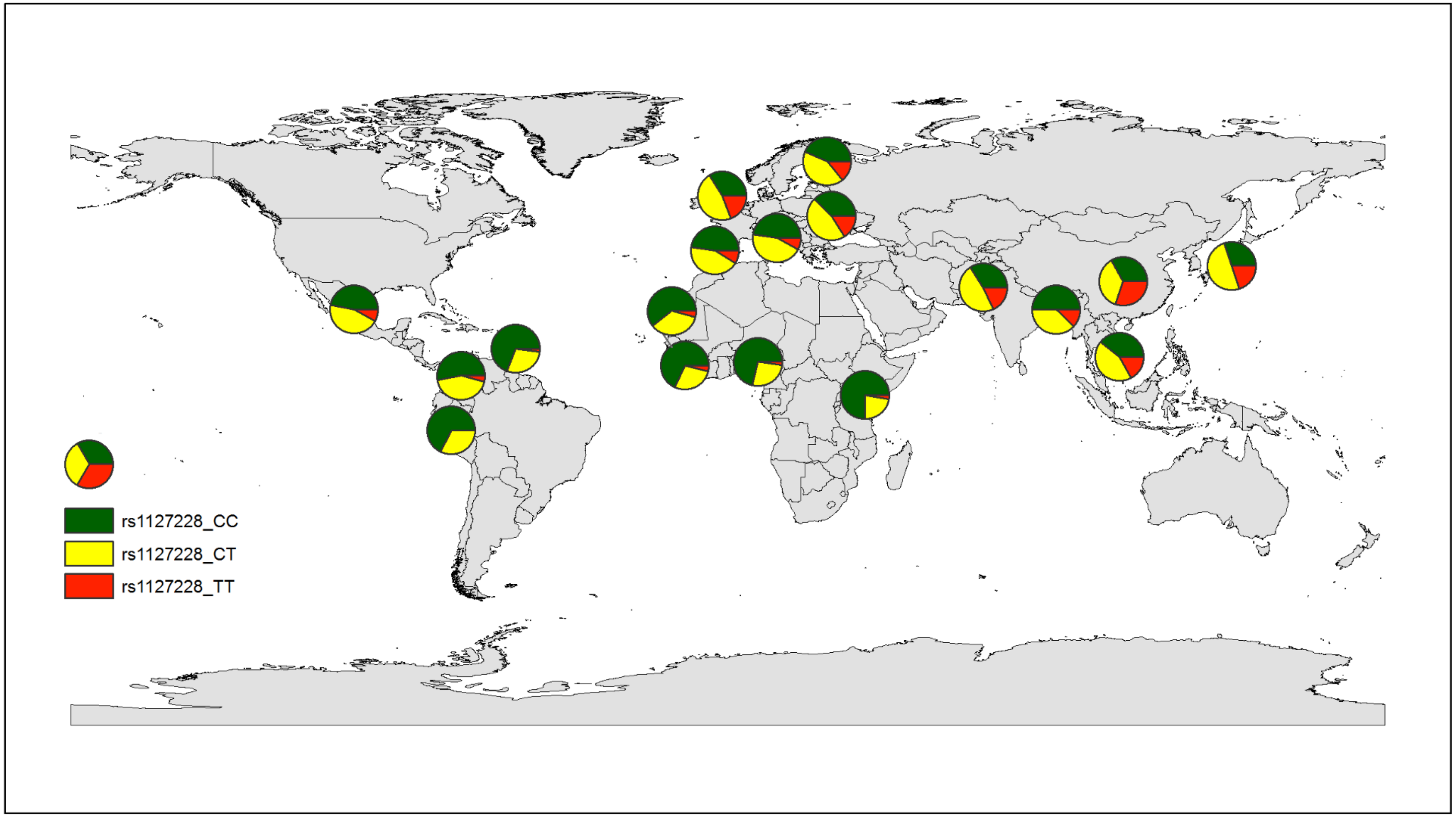

D

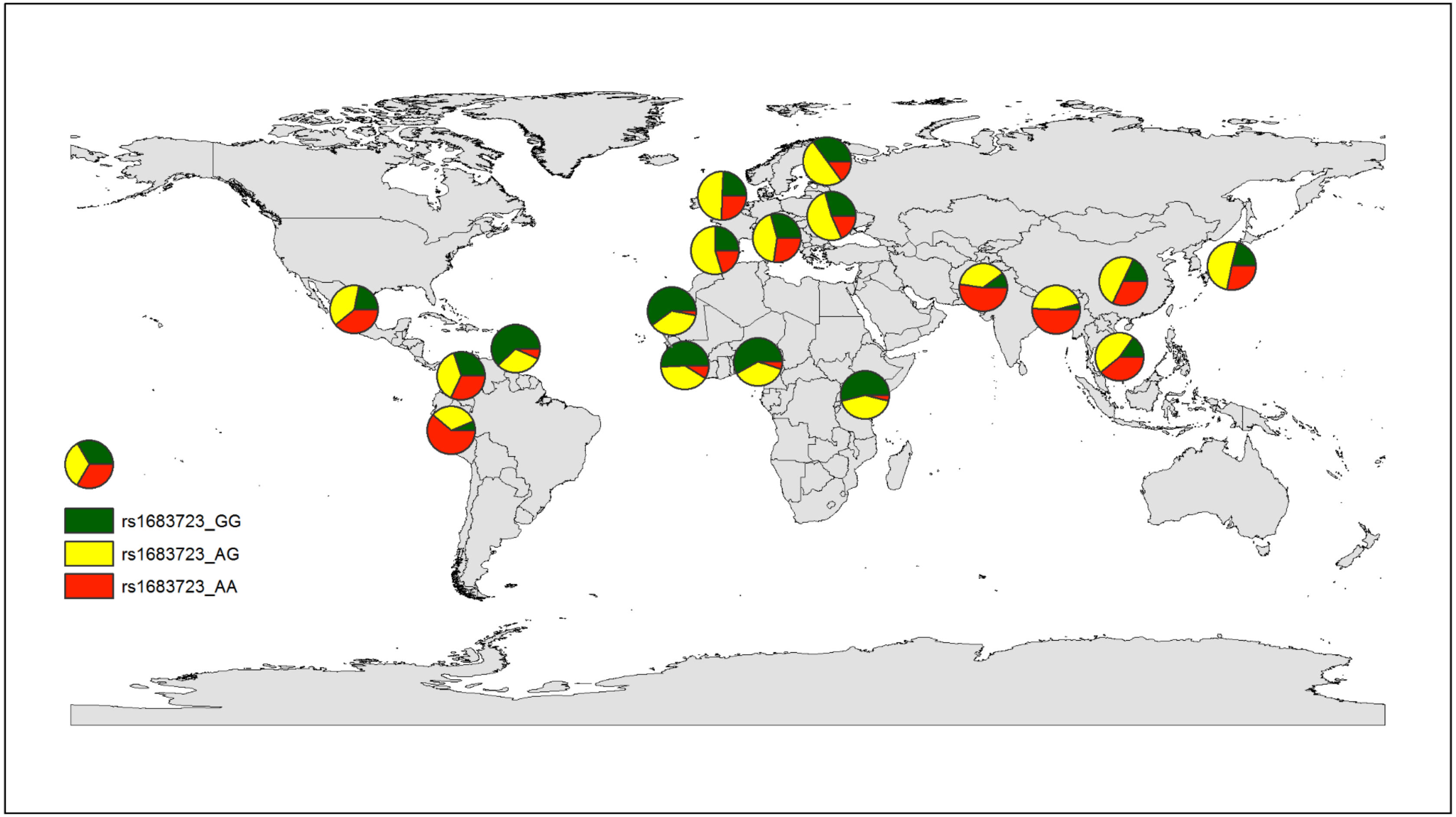

E

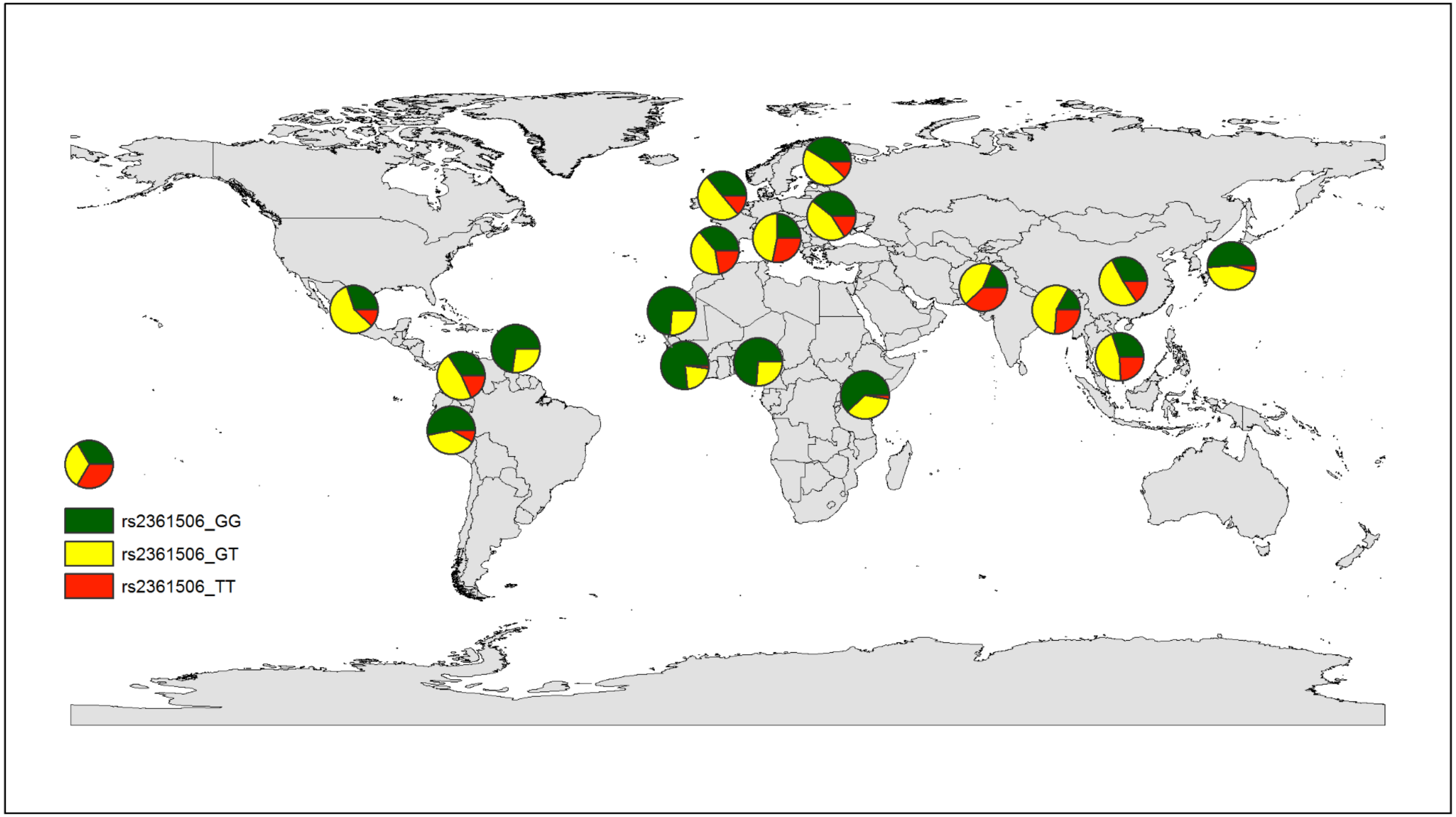

F

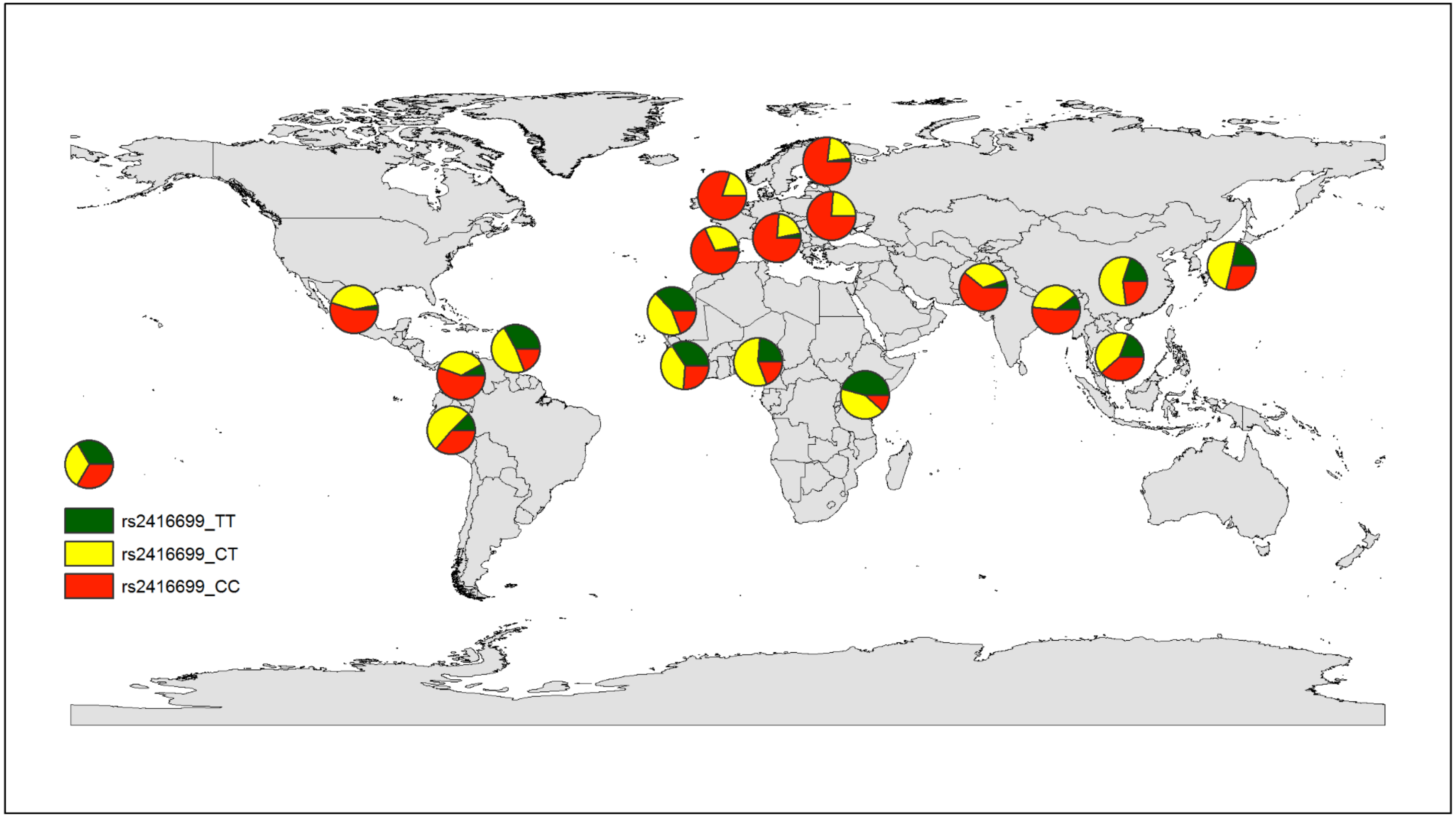

G

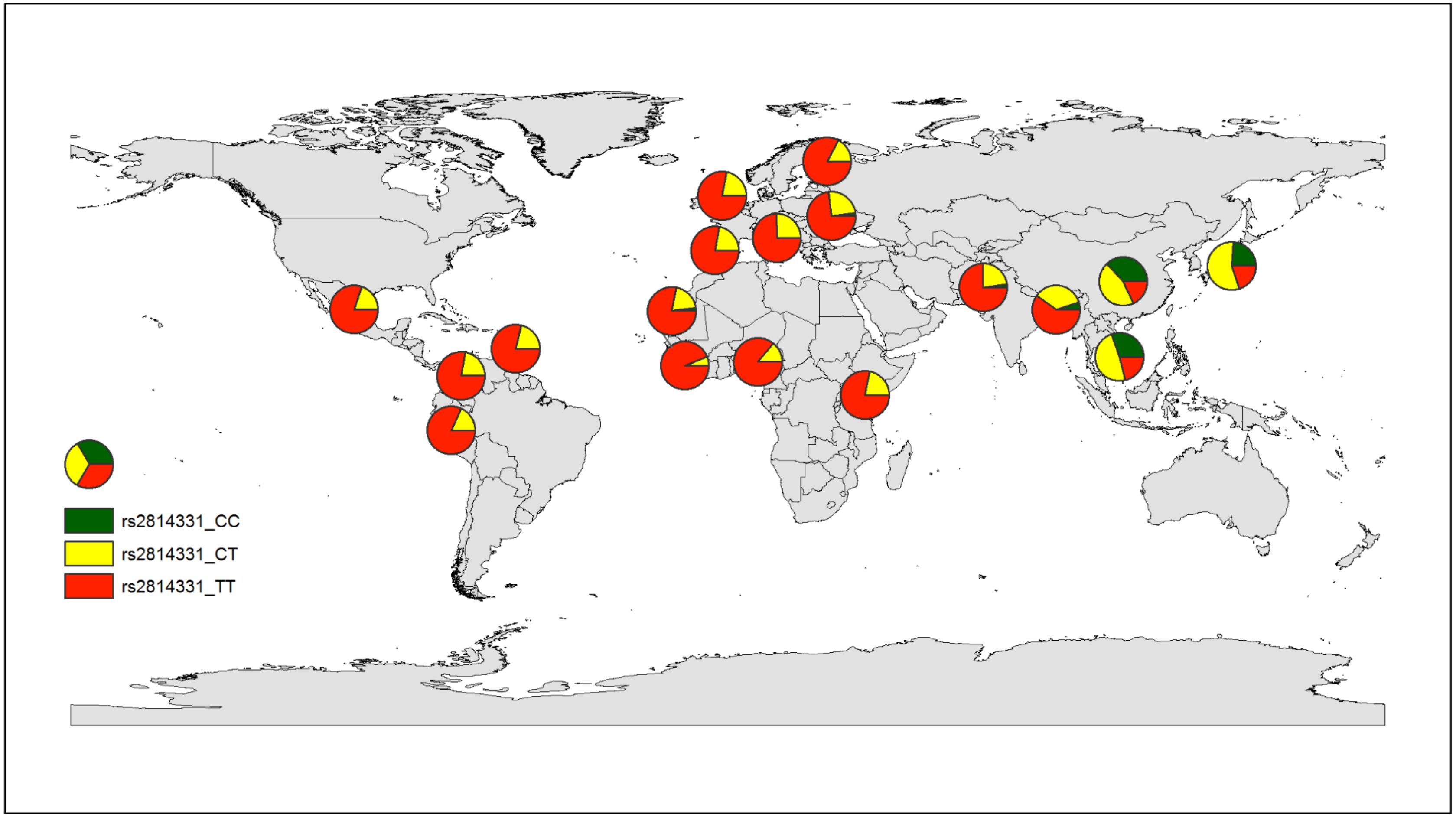

H

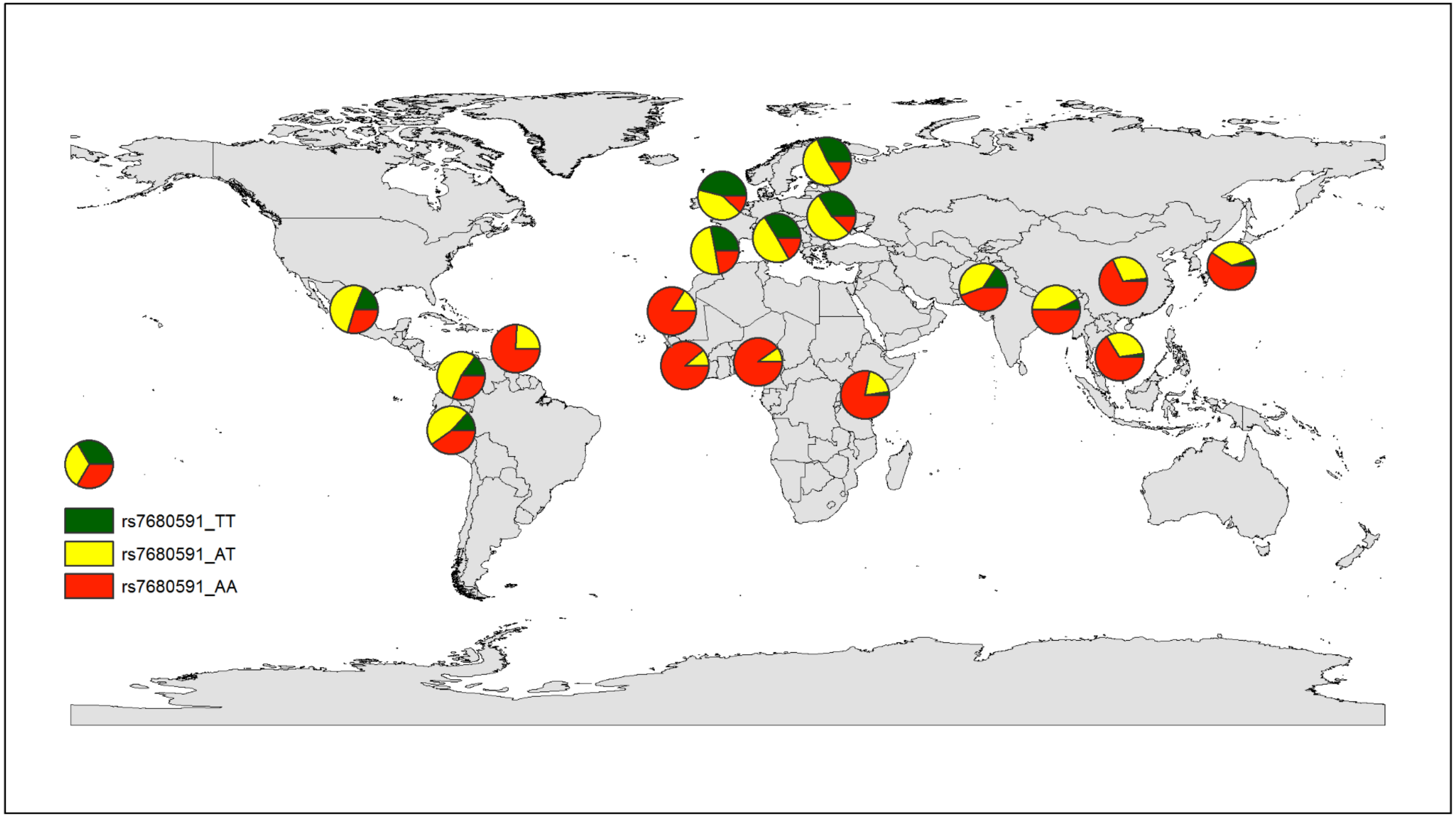

I

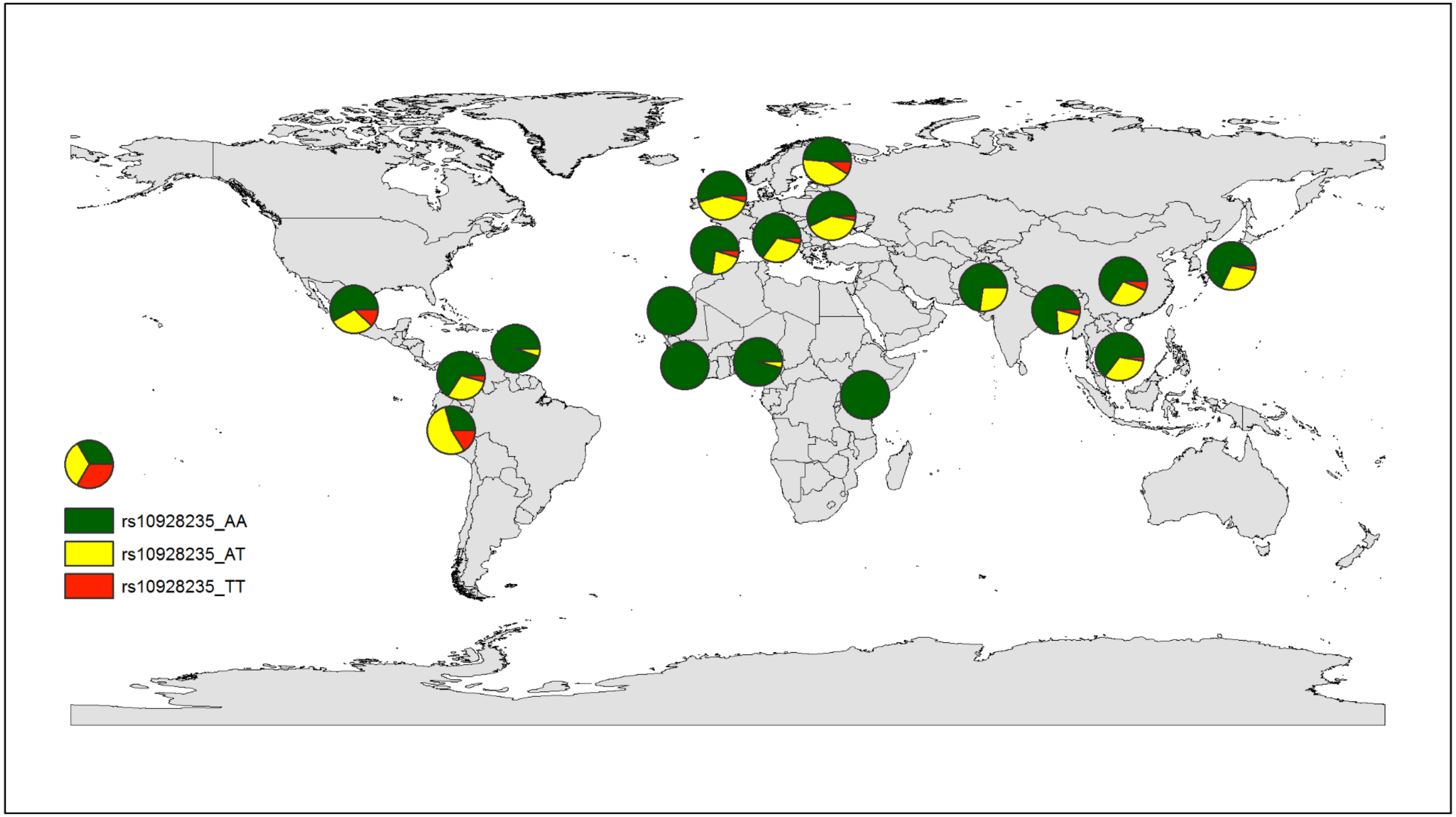

J

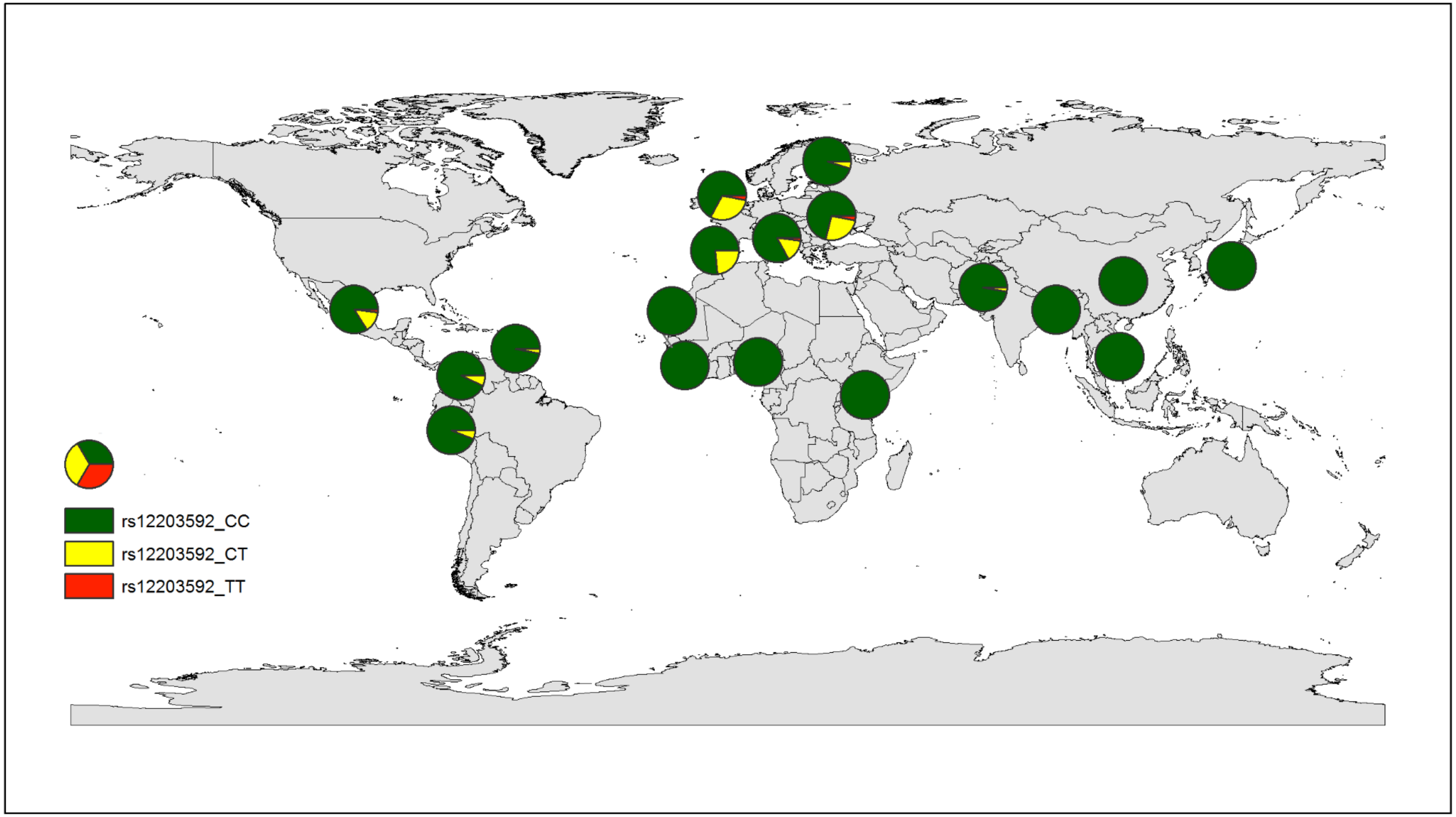

K

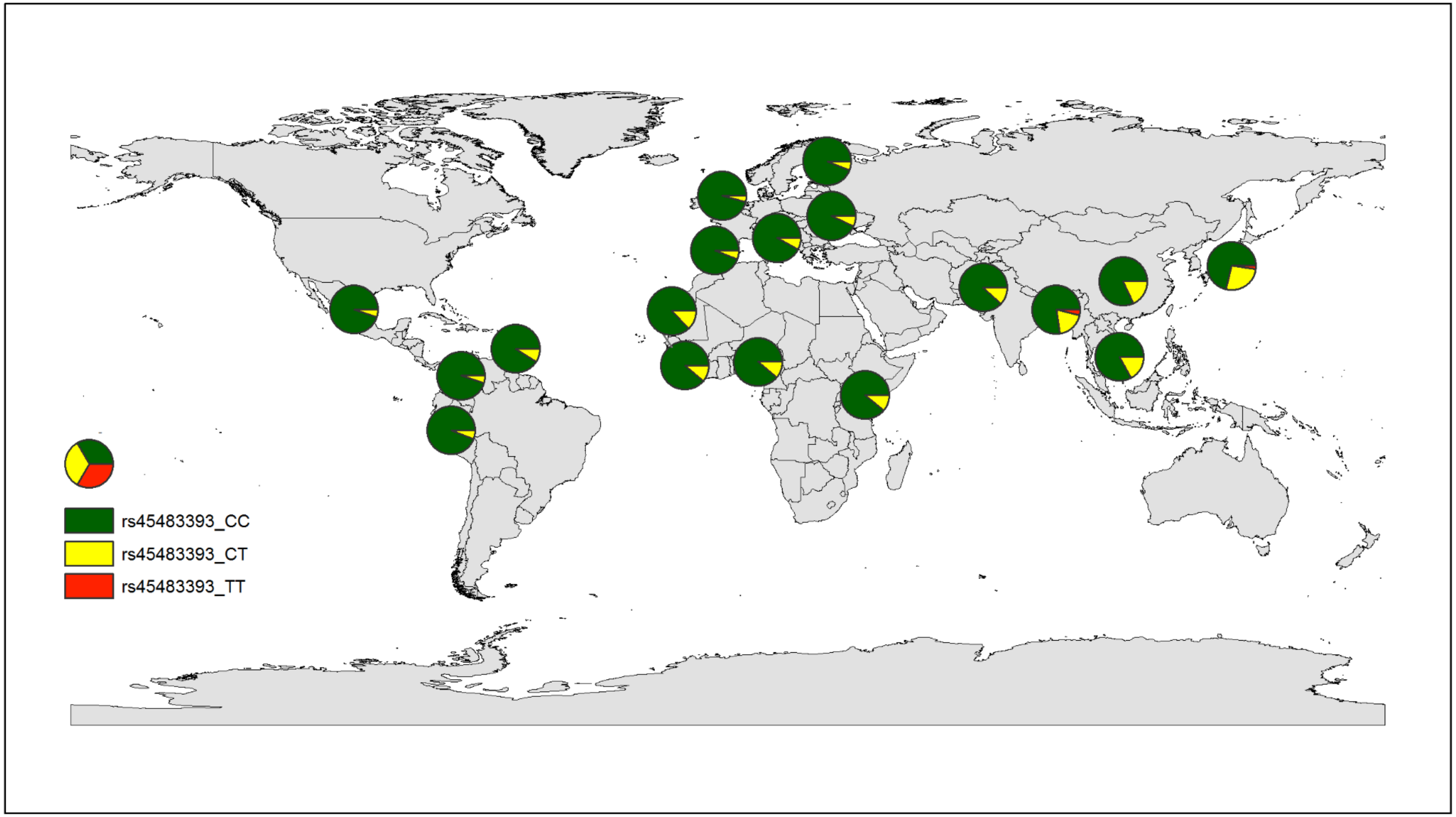

L

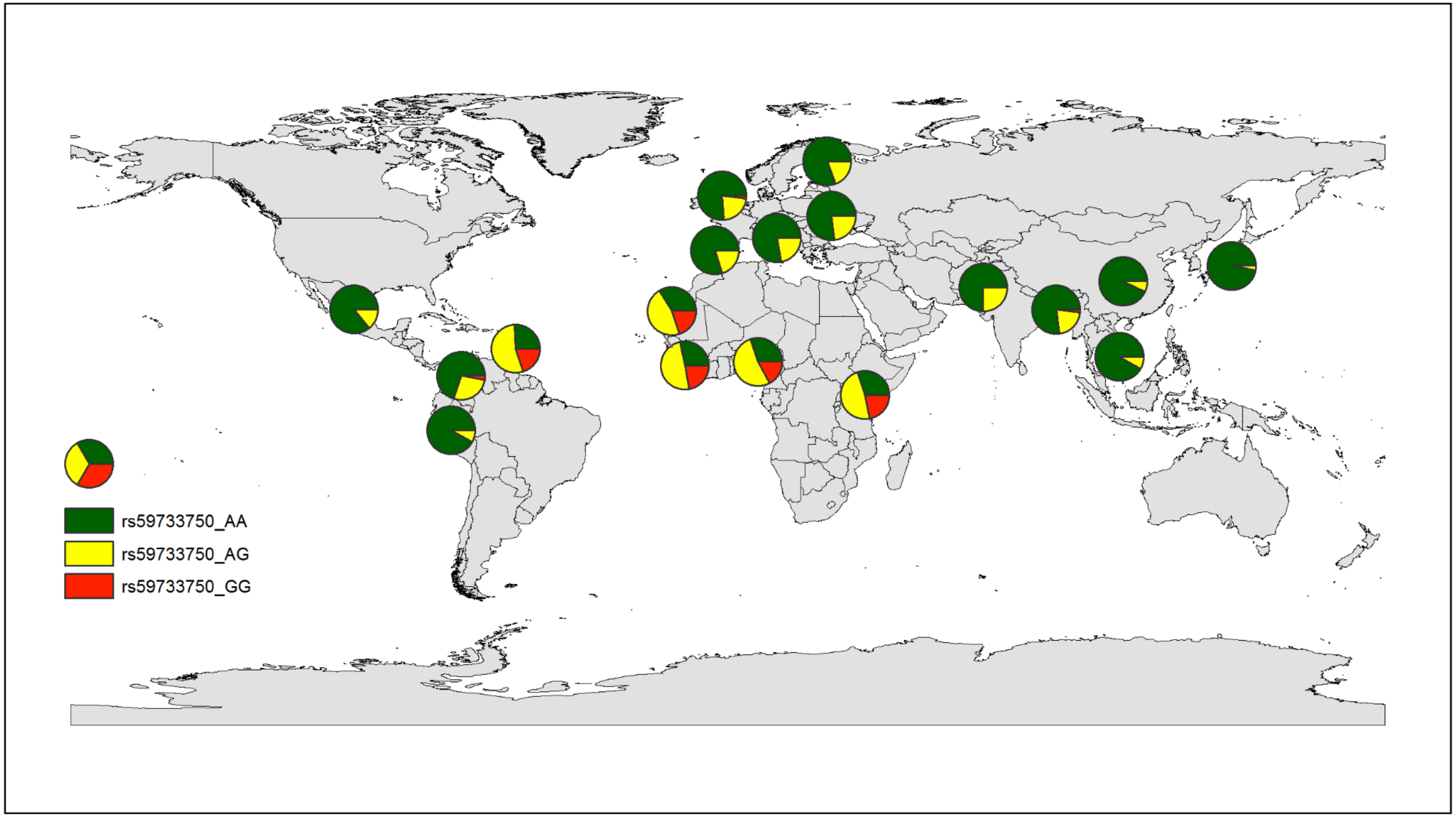

M

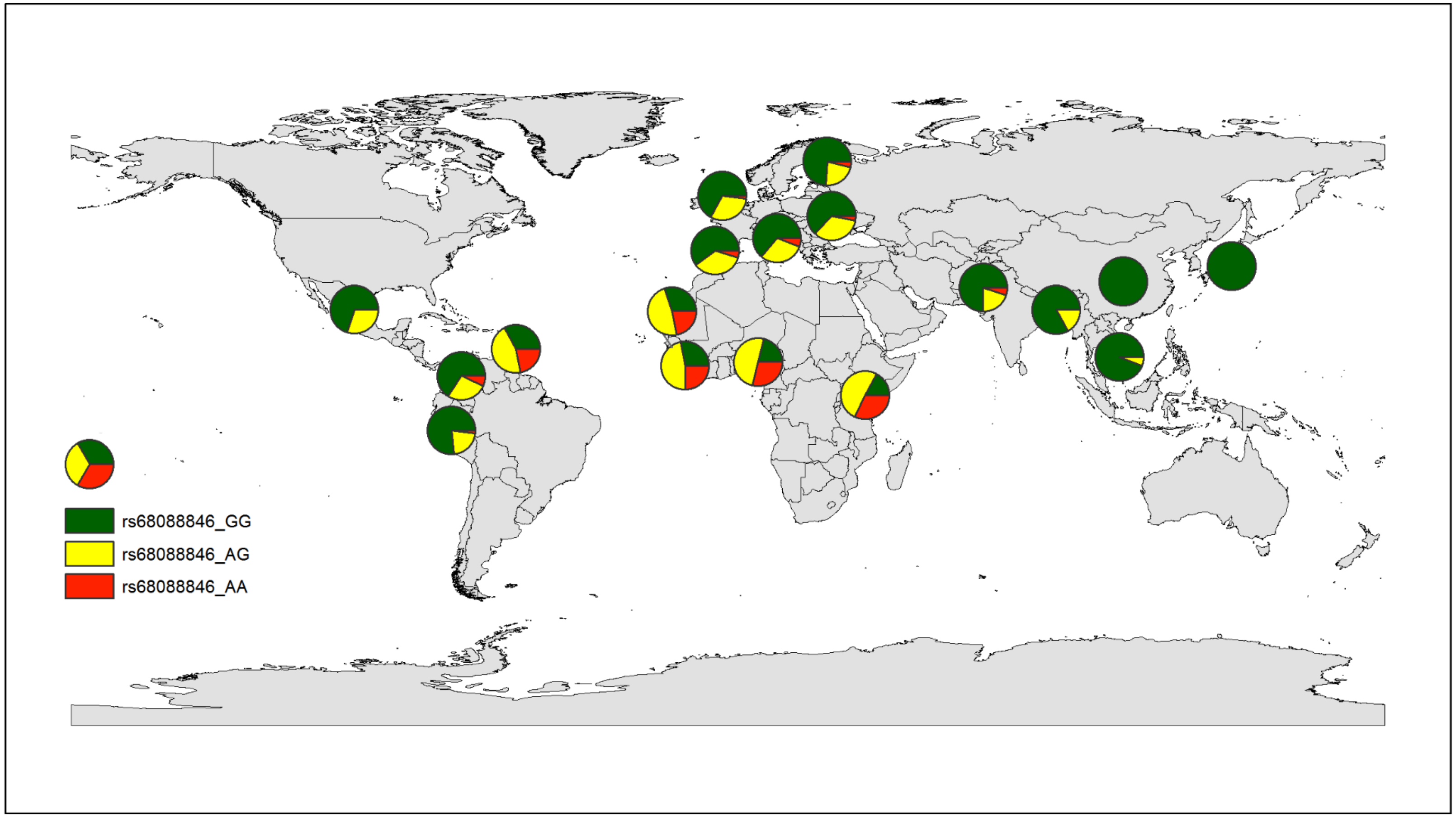

Supplementary Fig. 3 Global allele frequency distribution for 13 SNPs included in hair greying prediction models (**A** rs164741; **B** rs1005241; **C** rs1127228; **D** rs1683723; **E** rs2361506; **F** rs2416699; **G** rs2814331; **H** rs7680591; **I** rs10928235; **J** rs12203592; **K** rs45483393; **L**

rs59733750; **M** rs68088846). Allele frequencies for the selected SNPs were plotted on the world map using data from "The 1000 Genomes Project" (http://grch37.ensembl.org/Homo_sapiens/Info/Index) and ArcMap 10.7 under ArcGIS Desktop software (Esri, Redlands, California).

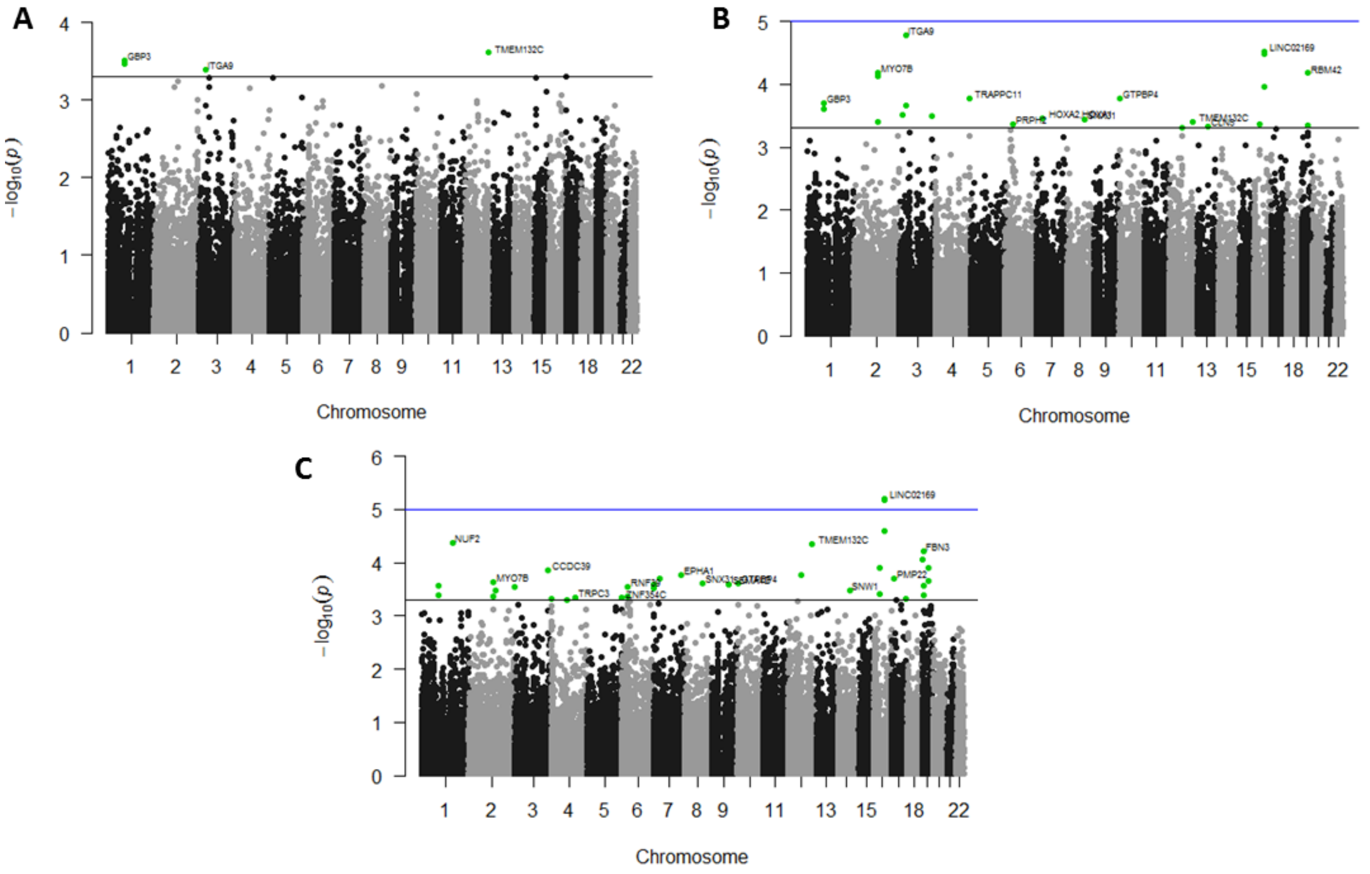

Supplementary Fig. 4 Manhattan plot of three EWAS analyses conducted in a 149 sample set for human head hair greying defined as A greying vs. no greying (BLR analysis); B no greying vs. mild greying vs. severe greying (MLR3 analysis); C 6-stage hair greying classification (MLR6 analysis). The -$\log_{10}$ (P-values) were plotted for each SNP under study according to its chromosomal position (GRCh38). The suggestive

significance threshold (*P*-value = 5×10^{-4}) is indicated as a black horizontal line and SNPs that reached the suggestive significance threshold are marked with green.

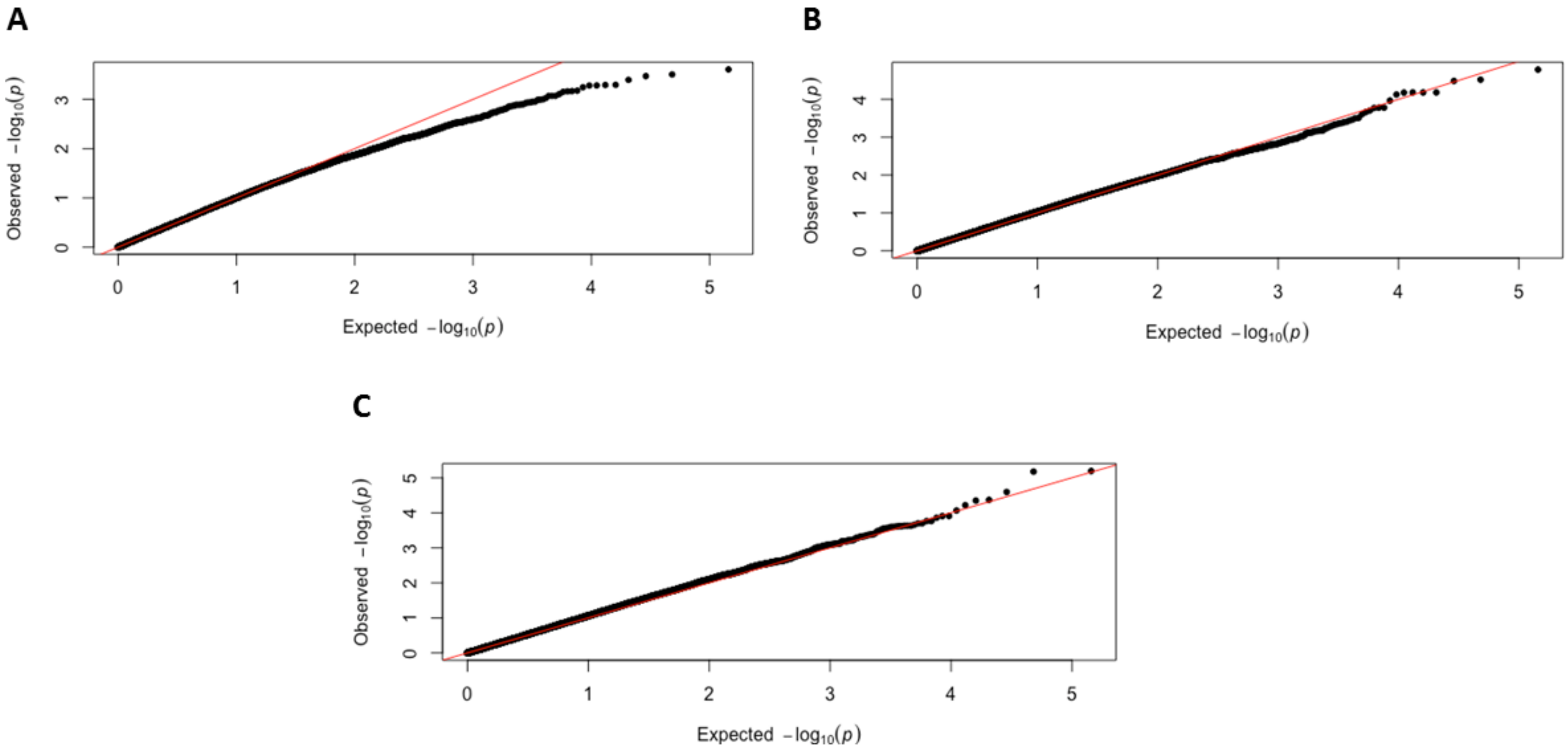

Supplementary Fig. 5 Q-Q plots of three EWAS analyses conducted in a 149 sample set for human head hair greying defined as A greying vs. no greying (BLR analysis); B no greying vs. mild greying vs. severe greying (MLR3 analysis); C 6-stage hair greying classification (MLR6 analysis).

Supplementary Table 1 Characteristics of the study population including discovery and replication/prediction modelling cohorts

Cohort	Hair greying category							
	Cat. 1	***Cat. 2***	***Cat. 3***	***Cat. 4***	***Cat. 5***	***Cat. 6***	***Cat. Grey***[a]	***Total***
Discovery cohort (N = 149)								
N [%]	89 [59.7]	33 [22.1]	14 [9.4]	10 [6.7]	2 [1.4]	1 [0.7]	-	149 [100.0]
Male [%]	43 [48.3]	18 [54.5]	9 [64.3]	10 [100.0]	2 [100.0]	1 [100.0]	-	83 [55.7]
Mean age ± SD	25.5±6.0	37.1±7.5	40.0±6.7	44.2±3.9	50.0±0.0	55	-	31.2±9.6
Replication and prediction modelling cohort (N = 849)								
N [%]	518 [61.0]	177 [20.8]	76 [9.0]	45 [5.3]	5 [0.6]	2 [0.2]	26 [3.1]	849 [100.0]
Male [%]	322 [62.2]	147 [83.1]	65 [85.5]	41 [9.1]	5 [100.0]	0 [0.0]	10 [38.5]	590 [69.5]
Mean age ± SD	26.1±6.5	35.0±7.5	37.9±6.7	41.8±5.0	46.4±7.4	50.0±17.0	38.6±5.9	30.4±8.7
Total cohort (N = 998)								
N [%]	607 [60.8]	210 [21.1]	90 [9.0]	55 [5.5]	7 [0.7]	3 [0.3]	26 [2.6]	998 [100.0]
Male [%]	365 [60.1]	165 [78.6]	74 [82.2]	51 [92.7]	7 [100.0]	1 [33.3]	10 [38.5]	673 [67.4]
Mean age ± SD	26.0±6.5	35.3±7.2	38.3±6.7	42.2±4.9	47.4±6.3	51.7±12.3	38.6±5.9	30.5±8.8

[a]for 26 samples only binary classification of greying vs. no greying was available

Supplementary Table 2 LD analysis conducted for exome-wide selected SNPs

SNP_ID_1	Chr position GRCh38	SNP_ID_2	Chr position GRCh38	r^2	SNP included in replication analysis
rs58719465	2:127609784	rs13422424	2:127624233	0.742	rs13422424
rs77159371	2:127620267	rs13422424	2:127624233	0.742	rs13422424
rs61744148	2:127621988	rs13422424	2:127624233	0.742	rs13422424
rs2507940	3:37494493	rs267561	3:37533460	0.801	rs2507940
rs17261971	2:127608697	rs58719465	2:127609784	0.808	rs13422424
rs17261971	2:127608697	rs77159371	2:127620267	0.808	rs13422424
rs17261971	2:127608697	rs61744148	2:127621988	0.808	rs13422424
rs73728107	6:30071046	rs11758516	6:30075709	0.867	rs11758516
rs72502509	6:30001413	rs11758516	6:30075709	0.881	rs11758516
rs34940876	16:27226713	rs1127228	16:27226789	0.983	rs1127228
rs36170987	7:259884	rs36139924	7:259915	0.986	rs36139924
rs58719465	2:127609784	rs77159371	2:127620267	1.000	rs13422424
rs58719465	2:127609784	rs61744148	2:127621988	1.000	rs13422424
rs77159371	2:127620267	rs61744148	2:127621988	1.000	rs13422424
rs17164384	4:1167692	rs6836335	4:1171960	1.000	rs6836335
rs72502509	6:30001413	rs73728107	6:30071046	1.000	rs11758516
rs2306408	10:1007010	rs2306406	10:1015922	1.000	rs2306406
rs72811791	16:54245728	rs16953255	16:54245775	1.000	rs72811791
rs72811791	16:54245728	rs72811792	16:54245781	1.000	rs72811791
rs16953255	16:54245775	rs72811792	16:54245781	1.000	rs72811791
rs72993508	19:8141675	rs72993510	19:8141684	1.000	rs56243829
rs72993508	19:8141675	rs56243829	19:8147279	1.000	rs56243829
rs72993510	19:8141684	rs56243829	19:8147279	1.000	rs56243829
rs4656076	1:89013171	rs3795543	1:89014075	1.000	rs4656076

Supplementary Table 3 A selection of exome-wide identified SNPs (*P*-value < 5 × 10^{-4}) associated with hair greying in a discovery cohort of 149 individuals from Poland

No.	SNP_ID	Chr position GRCh38	Gene/Locus	Discovery cohort (N = 149)							
				MA	fMA	BLR		MLR3		MLR6	
						beta	*P*-value*	beta	*P*-value*	beta	*P*-value*
1	rs4656076	1:89013171	*GBP3*	C	0.245	**2.031**	**3.115×10^{-4}**	**1.520**	**1.983×10^{-4}**	**1.350**	**2.658×10^{-4}**
2	rs2999852	1:163339512	*NUF2*	C	0.057	2.214	0.014	2.134	0.002	**2.499**	**4.262×10^{-5}**
3	rs13422424	2:127624233	*MYO7B*	T	0.151	2.147	5.668×10^{-4}	1.940	**7.452×10^{-5}**	**1.500**	**4.231×10^{-4}**
4	rs79386904	2:141015954	*LRP1B*	G	0.054	2.274	0.015	1.926	0.004	**2.209**	**3.311×10^{-4}**
5	rs59733750	2:240780193	*KIF1A*	G	0.144	-1.782	0.013	-1.787	0.002	**-1.861**	**2.798×10^{-4}**
6	rs74393830	3:23134423	*RPL24P7*	G	0.081	1.220	0.059	**1.992**	**3.096×10^{-4}**	1.496	6.113×10^{-4}
7	rs2507940	3:37494493	*ITGA9*	C	0.443	**2.066**	**4.009×10^{-4}**	**2.014**	**1.642×10^{-5}**	1.058	0.001
8	rs6836335	4:1171960	*SPON2*	C	0.107	0.972	0.093	1.441	0.004	**1.605**	**4.817×10^{-4}**
9	rs13131900	4:82580733	*AC067942.2*	G	0.151	2.601	7.018×10^{-4}	1.583	0.001	**1.427**	**4.945×10^{-4}**
10	rs1396082	4:121951564	*TRPC3*	G	0.487	0.400	0.286	0.969	0.005	**1.082**	**4.491×10^{-4}**
11	rs62358032	4:183693964	*TRAPPC11*	C	0.084	2.414	0.002	**2.252**	**1.689×10^{-4}**	1.663	7.619×10^{-4}
12	rs35780223	5:179083069	*ZNF354C*	T	0.067	2.237	0.012	1.602	0.011	**1.803**	**4.638×10^{-4}**
13	rs11758516	6:30075709	*RNF39*	C	0.057	2.509	0.002	2.222	$5.434x10^{-4}$	**2.119**	**2.860×10^{-4}**
14	rs7764439	6:42722017	*PRPH2*	A	0.456	0.627	0.092	**1.175**	**4.246×10^{-4}**	1.039	5.319×10^{-4}
15	rs36139924	7:259915	*FAM20C*	G	0.450	0.608	0.136	1.140	0.002	**1.151**	**2.520×10^{-4}**
16	rs10951154	7:27095695	*HOXA2;HOXA1*	C	0.148	1.332	0.012	**1.635**	**3.447×10^{-4}**	**1.518**	**2.000×10^{-4}**
17	rs62472724	7:143391804	*EPHA1*	T	0.138	1.879	0.004	1.736	6.867×10^{-4}	**1.744**	**1.714×10^{-4}**
18	rs2248609	8:100596691	*SNX31*	C	0.289	1.597	6.645×10^{-4}	**1.378**	**3.641×10^{-4}**	**1.254**	**2.463×10^{-4}**
19	rs45483393	9:89378809	*SEMA4D*	T	0.057	1.526	0.059	1.698	0.007	**1.996**	**2.513×10^{-4}**
20	rs2306406	10:1015922	*GTPBP4*	G	0.154	2.139	0.001	**1.734**	**1.664×10^{-4}**	**1.459**	**2.402×10^{-4}**
21	rs2228654	12:68871798	*CPM*	A	0.423	1.535	0.002	1.202	6.777×10^{-4}	**1.153**	**1.717×10^{-4}**
22	rs17120917	12:69796628	*RAB3IP*	C	0.218	1.609	0.002	**1.465**	**4.918×10^{-4}**	1.054	0.005
23	rs1683723	12:128415460	*TMEM132C*	A	0.399	**-1.993**	**2.462×10^{-4}**	**-1.397**	**3.916×10^{-4}**	**-1.422**	**4.462×10^{-5}**
24	rs1800209	13:77000848	*FBXL3; CLN5*	G	0.054	2.917	0.001	**2.496**	**4.615×10^{-4}**	2.146	7.492×10^{-4}
25	rs17752628	14:77732443	*SNW1*	C	0.054	3.534	0.007	1.905	0.016	**2.307**	**3.362×10^{-4}**
26	rs1127228	16:27226789	*NSMCE1*	T	0.362	-1.360	0.005	**-1.452**	**4.312×10^{-4}**	**-1.404**	**1.227×10^{-4}**
27	rs72811791	16:54245728	*LINC02169*	T	0.097	2.338	0.001	**2.471**	**3.035×10^{-5}**	**2.268**	**6.380×10^{-6}**

28	rs1804193	17:15230689	*PMP22*	T	0.144	-1.737	0.013	-2.087	6.956×10^{-4}	**-1.865**	**1.991×10^{-4}**
29	rs1043149	17:76081716	*EXOC7; ZACN*	T	0.161	-1.718	0.012	-1.948	0.002	**-1.881**	**4.648×10^{-4}**
30	rs778982	19:5866574	*FUT5*	C	0.329	1.291	0.014	1.261	0.003	**1.586**	**8.576×10^{-5}**
31	rs56243829	19: 8147279	*FBN3*	T	0.087	1.870	0.012	2.107	6.919×10^{-4}	**2.211**	**6.025×10^{-5}**
32	rs79338830	19: 35503484	*DMKN*	G	0.071	1.806	0.019	2.249	5.799×10^{-4}	**2.107**	**2.190×10^{-4}**
33	rs2285416	19:35637393	*RBM42*	A	0.158	1.559	0.006	**1.978**	**6.654×10^{-5}**	**1.591**	**1.240×10^{-4}**
34	rs547483	19:36950463	*ZNF568*	C	0.299	1.504	0.002	**1.254**	**4.552×10^{-4}**	0.958	0.001

Significant results are marked with bold.

BLR, binomial logistic regression; MLR3, multinomial ordinal logistic regression for 3 hair greying categories; MLR6, multinomial ordinal logistic regression for 6 hair greying categories; MA, minor allele; fMA, frequency of minor allele.

*Results adjusted for age and sex

Supplementary Table 4 Replication analysis of EWAS results in a replication cohort of 849 Polish individuals

No.	SNP_ID	Chr position GRCh38	Gene/Locus	Replication cohort (N = 849)							
				MA	fMA	BLR		MLR3		MLR6	
						beta	*P*-value[a]	beta	*P*-value[a]	beta	*P*-value[a]
1	rs4656076	1:89013171	*GBP3*	C	0.222	0.055	0.727	0.059	0.691	0.040	0.778
2	rs2999852	1:163339512	*NUF2*	C	0.051	0.097	0.728	0.104	0.675	0.187	0.423
3	rs13422424	2:127624233	*MYO7B*	T	0.178	0.047	0.775	-0.028	0.853	-0.076	0.598
4	rs79386904	2:141015954	*LRP1B*	G	0.044	-0.568	0.068	-0.493	0.103	-0.372	0.205
**5**	**rs59733750**	**2:240780193**	***KIF1A***	G	0.163	-0.484	**0.007**	-0.541	**0.002**	-0.569	**5.473×10^{-4}**
6	rs74393830	3:23134423	*RPL24P7*	G	0.091	-0.076	0.732	-0.138	0.510	-0.197	0.323
7	rs2507940	3:37494493	*ITGA9*	C	0.522	0.004	0.978	0.010	0.933	-0.015	0.896
8	rs6836335	4:1171960	*SPON2*	C	0.137	-0.014	0.942	-0.018	0.916	0.005	0.977
9	rs13131900	4:82580733	*AC067942.2*	G	0.141	0.079	0.665	0.097	0.570	0.152	0.348
10	rs34306906[b]	4:121944043	*TRPC3*	C	0.490	0.011	0.930	-0.051	0.681	-0.060	0.610
11	rs62358032	4:183693964	*TRAPPC11*	C	0.116	-0.074	0.719	0.080	0.666	0.146	0.402
12	rs35780223	5:179083069	*ZNF354C*	T	0.058	0.047	0.864	0.183	0.483	0.174	0.479
13	rs11758516	6:30075709	*RNF39*	C	0.043	-0.433	0.165	-0.507	0.116	-0.352	0.255
14	rs7764439	6:42722017	*PRPH2*	A	0.456	-0.061	0.645	0.009	0.940	-0.012	0.917
15	rs36139924	7:259915	*FAM20C*	G	0.462	-0.116	0.369	-0.073	0.543	0.012	0.914
16	rs10951154	7:27095695	*HOXA2;HOXA1*	C	0.159	0.092	0.602	-0.013	0.938	0.021	0.896
17	rs62472724	7:143391804	*EPHA1*	T	0.164	-0.085	0.631	-0.157	0.355	-0.163	0.314
18	rs2248609	8:100596691	*SNX31*	C	0.303	-0.215	0.125	-0.196	0.136	-0.161	0.196
19	rs45483393	9:89378809	*SEMA4D*	T	0.035	0.510	0.139	0.530	0.075	0.497	0.076
20	rs2306406	10:1015922	*GTPBP4*	G	0.204	0.054	0.732	0.027	0.858	0.050	0.727
21	rs2228654	12:68871798	*CPM*	A	0.437	0.190	0.163	0.101	0.426	0.092	0.443
22	rs17120917	12:69796628	*RAB3IP*	C	0.247	-0.124	0.410	-0.176	0.214	-0.162	0.228
23	rs1683723	12:128415460	*TMEM132C*	A	0.433	0.080	0.533	0.135	0.267	0.158	0.168
24	rs1800209	13:77000848	*FBXL3; CLN5*	G	0.078	-0.359	0.150	-0.377	0.112	-0.304	0.183
25	rs17752628	14:77732443	*SNW1*	C	0.067	0.227	0.383	0.216	0.365	0.174	0.437
**26**	**rs1127228**	**16:27226789**	***NSMCE1***	T	0.347	-0.241	0.079	-0.314	**0.015**	-0.283	**0.022**
27	rs72811791	16:54245728	*LINC02169*	T	0.099	0.048	0.818	0.069	0.727	0.076	0.687
28	rs1804193	17:15230689	*PMP22*	T	0.118	0.236	0.234	0.184	0.318	0.130	0.454

29	rs1043149	17:76081716	*EXOC7; ZACN*	T	0.173	-0.117	0.486	-0.154	0.336	-0.080	0.602
30	rs778982	19:5866574	*FUT5*	C	0.254	-0.040	0.788	-0.070	0.615	-0.113	0.397
31	rs72993531[b]	19:8147640	*FBN3*	T	0.115	0.035	0.862	-0.034	0.863	-0.045	0.811
32	rs77995042[b]	19:35508296	*DMKN*	T	0.056	0.088	0.745	-0.027	0.913	-0.045	0.847
33	rs2285416	19:35637393	*RBM42*	A	0.127	0.093	0.631	-0.013	0.942	-0.029	0.863
34	rs547483	19:36950463	*ZNF568*	C	0.318	0.017	0.904	0.131	0.326	0.052	0.681

Significant results are marked with bold.

BLR, binomial logistic regression; MLR3, multinomial ordinal logistic regression for 3 hair greying categories; MLR6, multinomial ordinal logistic regression for 6 hair greying categories; MA, minor allele; fMA, frequency of minor allele.

[a]Results adjusted for age and sex

[b]Due to technical difficulties three SNPs: *TRPC3* rs1396082, *FBN3* rs56243829 and *DMKN* rs79338830 were replaced by different SNPs in LD (*TRPC3* rs34306906, *FBN3* rs72993531 and *DMKN* rs77995042)

Supplementary Table 5 Results of association testing for 336 literature-based selected candidate SNPs for hair greying associated previously with hair colour/pigmentation, hair loss, hair shape and hair thickness

No.	SNP_ID	Chr position GRCh38	Gene	fMA	BLR		MLR3		MLR6		Trait association	References
					beta	*P*-value*	beta	*P*-value*	beta	*P*-value*		
1	**rs80293268**	**1:8147519**	***ERRFI1; SLC45A1***	C 0.08	**-0.483**	**0.048**	-0.390	0.090	-0.366	0.093	Hair shape	[1,2]
2	rs6658216	1:10501547	*PEX14*	C 0.30	-0.083	0.554	-0.002	0.988	0.037	0.766	Hair shape	[1,2]
3	rs10864459	1:10503552	*PEX14*	A 0.31	-0.076	0.584	-0.007	0.959	0.032	0.794	Hair shape	[1]
4	rs2847344	1:10504618	*PEX14*	G 0.32	-0.093	0.499	-0.022	0.866	0.012	0.923	Hair shape	[1]
5	rs12565727	1:10973025	*C1orf127*	G 0.22	-0.297	0.064	-0.222	0.141	-0.224	0.119	Hair loss	[3-5]
6	rs11203346	1:17274327	*PADI3*	G 0.16	-0.085	0.627	-0.096	0.550	-0.059	0.695	Hair shape	[1,2]
7	rs11585118	1:17279279	*PADI3*	A 0.15	-0.098	0.581	-0.112	0.497	-0.077	0.620	Hair shape	[6]
8	rs9803789	1:25141755	1p36.11	G 0.35	-0.100	0.450	-0.011	0.931	-0.024	0.839	Hair loss	[7]
9	rs16827770	1:40918052	*CITED4*	G 0.30	0.216	0.133	0.118	0.386	0.134	0.302	Hair loss	[8]
10	rs17371253	1:47489755	*FOXD2*	T 0.43	0.005	0.970	-0.125	0.305	-0.141	0.222	Hair loss	[9]
11	rs10888690	1:50494849	*FAF1*	C 0.30	0.017	0.902	0.006	0.961	-0.075	0.539	Hair loss	[8]
12	rs10874518	1:101806756	*OLFM3*	C 0.35	-0.123	0.381	0.009	0.946	0.037	0.762	Pigmentation	[10]
13	rs111668293	1:118896200	*TBX15; WARS2*	- 0.16	-0.130	0.470	-0.087	0.601	-0.024	0.878	Hair loss	[7]
14	rs17185996	1:119066109	*WARS2*	C 0.18	-0.168	0.310	-0.277	0.077	-0.270	0.069	Hair loss	[9]
15	rs11204897	1:151836590	*LOC100132111*	G 0.11	-0.039	0.839	-0.014	0.937	-0.020	0.906	Hair shape	[1-2]
16	rs75203436	1:151869882	1q21.3	G 0.06	0.075	0.787	-0.015	0.953	0.003	0.991	Hair shape	[1-2]
17	rs55883933	1:151916971	1q21.3	C 0.22	0.108	0.474	0.005	0.974	0.048	0.724	Hair shape	[1-2]
18	rs10788819	1:151922617	*LOC105373470*	T 0.21	0.130	0.399	0.025	0.865	0.074	0.593	Hair shape	[1,6,11]
19	rs114410520	1:152000133	*LOC105371441*	C 0.02	0.438	0.372	0.264	0.547	0.287	0.481	Hair shape	[1-2]
20	rs3007671	1:152026871	*NBPF18P;S100A11*	T 0.30	0.065	0.641	0.030	0.821	0.132	0.288	Hair shape	[1,6,11]
21	rs151069963	1:152031765	1q21.3	C 0.01	0.891	0.081	0.863	0.077	0.841	0.066	Hair shape	[1-2]
22	rs3007681	1:152046881	*LOC101927949*	C 0.50	0.023	0.854	0.010	0.931	-0.039	0.733	Hair shape	[1-2]
23	rs12130862	1:152054539	*near TCHH*	T 0.25	0.205	0.157	0.141	0.295	0.241	0.059	Hair shape	[1,6,11,12]
24	rs17646946	1:152090291	*TCHHL1*	A 0.25	0.183	0.204	0.115	0.393	0.201	0.115	Hair shape	[1,2,12]
25	rs11204925	1:152100644	1q21.3	G 0.30	0.119	0.395	0.180	0.175	0.131	0.298	Hair shape	[1-2]
26	**rs1131471**	**1:152107513**	***TCHH***	G 0.43	-0.247	0.058	-0.237	0.052	**-0.279**	**0.016**	Hair shape	[1-2]
27	rs11803731	1:152110849	*TCHH*	T 0.25	0.186	0.200	0.129	0.341	0.212	0.096	Hair shape	[1,6,11,12]

28	rs72696935	1:152113475	*TCHH*	G 0.04	0.083	0.794	0.020	0.944	0.034	0.901	Hair shape	[1-2]
29	rs36010924	1:152116368	*PUDPP2; TCHH*	G 0.25	0.186	0.200	0.129	0.341	0.212	0.096	Hair loss	[8]
30	rs140371183	1:152125952	1q21.3	G 0.01	0.295	0.668	0.148	0.818	0.096	0.872	Hair shape	[1-2]
31	**rs3001978**	**1:152153991**	***RPTN***	C 0.43	-0.247	0.058	-0.237	0.052	**-0.279**	**0.016**	Hair shape	[1,6,11,13]
32	rs4845418	1:152163754	*near TCHH*	C 0.20	0.149	0.341	0.089	0.539	0.182	0.183	Hair shape	[1,6,11,12]
33	rs10788826	1:152189259	1q21.3	C 0.30	-0.119	0.405	-0.123	0.363	-0.128	0.321	Hair shape	[1-2]
34	rs74868796	1:152218575	*HRNR*	A 0.02	-0.290	0.420	-0.028	0.933	0.130	0.668	Hair shape	[1-2]
35	rs61816764	1:152336495	*FLG-AS1*	T 0.04	-0.550	0.096	-0.514	0.116	-0.511	0.105	Hair shape	[1-2]
36	rs2050631	1:152340121	*FLG-AS1*	G 0.35	0.067	0.611	0.053	0.668	0.063	0.590	Hair shape	[1-2]
37	rs2146114	1:152418145	1q21.3	C 0.14	-0.078	0.676	-0.001	0.995	-0.013	0.939	Hair shape	[1-2]
38	rs12116760	1:152465407	1q21.3	C 0.38	-0.087	0.519	-0.081	0.517	-0.117	0.323	Hair shape	[1-2]
39	rs12123907	1:152495275	1q21.3	A 0.33	0.015	0.917	0.134	0.308	0.157	0.209	Hair shape	[1-2]
40	rs4845779	1:152506700	*CRNN; LCE5A*	T 0.25	-0.192	0.197	-0.203	0.149	-0.199	0.135	Hair shape	[1-2]
41	rs499697	1:152520678	*CRCT1; near LCE3E*	G 024	-0.201	0.185	-0.215	0.136	-0.203	0.137	Hair shape	[1,6,11]
42	rs115813648	1:152949110	*LINC01527*	C 0.00	0.555	0.472	0.414	0.578	0.485	0.475	Hair shape	[1-2]
43	rs10919382	1:170392023	*GORAB*	G 0.33	-0.003	0.980	-0.104	0.423	-0.070	0.573	Hair loss	[7]
44	rs2206310	1:170803635	*PRRX1; MROH9*	G 0.35	0.042	0.755	-0.017	0.890	0.038	0.749	Hair loss	[9]
45	rs78448052	1:203996272	*SOX13*	T 0.02	0.311	0.486	0.119	0.779	0.034	0.931	Hair loss	[8]
46	rs3002288	1:212953223	*VASH2*	A 0.45	0.098	0.469	0.087	0.487	0.055	0.640	Pigmentation	[14]
47	rs4846480	1:218425127	*TGFB2*	T 0.25	-0.044	0.764	-0.085	0.535	-0.059	0.649	Hair shape	[15]
48	rs3768056	1:235744525	*LYST*	G 0.22	0.153	0.323	0.096	0.500	0.099	0.463	Pigmentation	[16]
49	rs12997617	2:6413568	2p25.2	T 0.14	0.073	0.688	0.202	0.229	0.175	0.271	Hair loss	[9]
50	rs6752754	2:6429434	*ESPN*	G 0.15	-0.006	0.972	0.160	0.337	0.157	0.322	Hair loss	[8]
51	rs7586898	2:15821475	*DDX1; RNU5E-7P*	G 0.17	0.274	0.101	0.174	0.261	0.087	0.555	Hair shape	[11]
52	rs844193	2:30403557	2p23.1	T 0.20	0.000	0.999	0.058	0.703	0.075	0.604	Hair loss	[8]
53	rs13021718	2:31956355	*MEMO1*	A 0.13	-0.214	0.269	-0.098	0.591	0.023	0.896	Hair loss	[7]
54	rs6732426	2:43360365	*THADA*	T 0.49	-0.032	0.810	0.039	0.755	0.000	0.998	Hair shape	[11]
55	rs17035085	2:46378148	*EPAS1*	G 0.01	-0.170	0.745	-0.401	0.446	-0.500	0.320	Hair shape	[11]
56	rs12464462	2:60409281	2p16.1	G 0.42	-0.111	0.394	-0.167	0.176	-0.101	0.390	Hair loss	[8]
57	rs9989836	2:70115595	2p13.3	A 0.31	-0.261	0.063	-0.220	0.096	-0.204	0.105	Hair shape	[1-2]
58	rs2706768	2:70229454	*TIA1*	T 0.39	-0.101	0.433	-0.058	0.630	-0.042	0.713	Hair loss	[8]
59	rs12997742	2:70559466	*TGFA*	C 0.40	0.120	0.360	0.055	0.649	0.085	0.455	Hair shape	[1-2]
60	rs205611	2:74744954	2p13.1	G 0.18	-0.051	0.762	-0.026	0.873	-0.036	0.815	Pigmentation	[10]
61	rs3827760	2:108897145	*EDAR*	G 0.01	-0.754	0.221	-0.293	0.606	-0.277	0.624	Hair shape	[17-19]

62	rs365060	2:108959280	*EDAR*	G 0.07	-0.051	0.838	0.038	0.868	0.111	0.610	Hair thickness	[20]
63	**rs10928235**	**2:144920547**	***TEX41***	T 0.24	0.253	0.080	**0.264**	**0.049**	0.177	0.165	Hair loss	[8]
64	rs13405699	2:173740905	*CDCA7*	A 0.03	-0.044	0.908	0.007	0.984	0.119	0.717	Hair loss	[8]
65	**rs71421546**	**2:176127129**	***HOXD-AS2;HOXD3***	A 0.04	-0.660	0.050	**-0.687**	**0.037**	-0.580	0.064	Hair loss	[8]
66	rs881921	2:176831781	*HNRNPA3*	G 0.44	-0.178	0.163	-0.124	0.304	-0.099	0.385	Hair loss	[8]
67	rs692243	2:218830764	*PRKAG3*	C 0.18	-0.203	0.230	-0.228	0.151	-0.125	0.403	Hair shape	[1-2]
68	rs10193725	2:218861775	*WNT6*	C 0.17	-0.096	0.561	-0.120	0.440	-0.009	0.954	Hair loss	[4,5,21]
69	rs74333950	2:218881570	*WNT10A*	G 0.12	-0.334	0.116	-0.310	0.121	-0.214	0.257	Hair shape	[1-2]
70	rs7349332	2:218891661	*WNT10A*	T 0.11	-0.386	0.071	-0.345	0.087	-0.255	0.179	Hair loss/shape	[1,2,6,11,12,21]
71	rs77157375	2:218915189	*LINC01494*	A 0.02	-0.427	0.380	-0.728	0.136	-0.440	0.353	Hair shape	[1-2]
72	**rs4672907**	**2:218956447**	***LOC391485***	A 0.15	**-0.388**	**0.033**	-0.187	0.271	-0.177	0.275	Hair shape	[1,6,11]
73	rs2218065	2:222169363	2q36.1	A 0.35	0.076	0.572	-0.010	0.939	-0.015	0.896	Hair thickness	[20]
74	rs77177529	2:222205957	*PAX3*	T 0.08	-0.038	0.868	-0.152	0.490	-0.208	0.330	Hair loss	[7,8]
75	rs6742078	2:233763993	*UGT1A*	T 0.36	0.092	0.507	-0.065	0.610	0.002	0.986	Pigmentation	[22]
76	rs79693451	2:238387832	*TRAF3IP1*	G 0.07	-0.179	0.494	0.024	0.922	0.032	0.894	Pigmentation	[10]
77	rs9287638	2:238785990	2q37.3	A 0.33	0.189	0.160	0.117	0.356	0.123	0.304	Hair loss	[3,4,5]
78	rs11684254	2:238787252	*TWIST2*	G 0.34	0.188	0.155	0.140	0.262	0.145	0.219	Hair loss	[7,8]
79	rs9751918	2:238827171	2q37.3	G 0.21	0.200	0.224	0.166	0.276	0.181	0.207	Hair loss	[3,5]
80	rs9846246	3:107705243	*BBX*	G 0.48	0.125	0.325	0.016	0.895	-0.026	0.819	Hair loss	[8]
81	rs9850626	3:126349936	*KLF15*	G 0.21	-0.252	0.116	-0.160	0.291	-0.173	0.230	Hair loss	[9]
82	rs35892873	3:126350105	*KLF15*	T 0.28	0.257	0.069	0.146	0.264	0.112	0.365	Hair loss	[8]
83	rs75937217	3:138957929	*FOXL2*	C 0.00	1.675	0.091	1.309	0.142	1.320	0.096	Hair thickness	[20]
84	rs7642536	3:139313491	*MRPS22*	C 0.15	-0.254	0.165	-0.266	0.130	-0.255	0.131	Hair loss	[7]
85	rs11714208	3:141576208	*RASA2; ALPL*	G 0.34	0.067	0.633	-0.050	0.706	-0.026	0.835	Hair loss	[8]
86	rs7648585	3:151921977	*AADACL2; AS1*	G 0.38	0.158	0.234	0.048	0.700	0.075	0.523	Hair loss	[5,21]
87	rs4679955	3:151935580	*SUCNR1*	T 0.41	0.087	0.505	0.002	0.989	0.019	0.866	Hair loss/shape	[4,5,12,21]
88	rs16863765	3:151956689	*AADAC*	A 0.33	0.064	0.642	0.067	0.601	0.048	0.692	Hair loss	[8]
89	rs55854644	3:185508704	*LIPH*	T 0.30	0.062	0.668	0.084	0.536	0.094	0.462	Hair shape	[23]
90	rs505569	3:185518327	*LIPH*	G 0.45	0.097	0.454	0.130	0.287	0.119	0.304	Hair shape	[23]
91	rs551936	3:185545679	*LIPH*	C 0.17	-0.038	0.827	-0.062	0.697	-0.109	0.469	Hair shape	[23]
92	rs4864809	4:53645746	*LNX1*	A 0.35	0.135	0.324	0.102	0.429	0.059	0.633	Hair thickness	[20]
93	rs436034	4:78334882	*FRAS1*	T 0.25	0.084	0.563	0.171	0.214	0.150	0.252	Hair shape	[1,11]
94	rs1268789	4:78359539	*FRAS1*	T 0.31	0.014	0.918	0.052	0.688	0.060	0.626	Hair shape	[1,2,11-13]

95	**rs7680591**	**4:80276795**	***FGF5***	A 0.42	**-0.340**	**0.011**	**-0.376**	**0.003**	**-0.347**	**0.004**	Hair loss	[7]
96	rs142756290	4:104952656	*CXXC4; TET2*	C 0.06	0.134	0.616	0.217	0.370	0.049	0.831	Hair loss	[9]
97	rs12509636	4:105089276	*TET2*	T 0.39	-0.005	0.970	-0.003	0.982	-0.065	0.599	Hair loss	[8]
98	rs76067940	4:107131838	*DKK2*	T 0.03	-0.216	0.527	-0.387	0.257	-0.397	0.221	Hair loss	[8]
99	rs17316633	4:109904478	*EGF*	A 0.25	0.067	0.658	-0.044	0.759	0.004	0.975	Hair shape	[11]
100	rs724818	4:120705958	*PRDM5*	A 0.02	-0.520	0.243	-0.521	0.231	-0.371	0.376	Hair thickness	[20]
101	rs4864363	4:136770595	4q28.3	G 0.14	-0.076	0.678	-0.124	0.472	-0.155	0.349	Hair shape	[20]
102	rs6840361	4:162816969	*NAF1*	G 0.05	0.162	0.583	-0.044	0.879	-0.185	0.502	Hair shape	[11]
103	rs17053607	4:167868421	*ANXA10*	A 0.03	0.127	0.696	0.109	0.725	0.188	0.525	Hair loss	[9]
104	rs13140875	4:167936311	*ANXA10*	C 0.11	-0.181	0.377	-0.316	0.104	-0.148	0.414	Pigmentation	[10]
105	rs12520016	5:6767199	*LOC102724943*	G 0.05	-0.282	0.342	-0.379	0.185	-0.265	0.337	Pigmentation	[14]
106	rs16891982	5:33951588	*SLC45A2*	C 0.04	-0.094	0.780	-0.131	0.690	-0.131	0.680	Pigmentation	[24-28]
107	rs28777	5:33958854	*SLC45A2*	C 0.03	-0.075	0.829	-0.120	0.731	-0.110	0.741	Pigmentation	[25,29]
108	rs26722	5:33963765	*SLC45A2*	T 0.02	-0.141	0.732	0.119	0.760	0.126	0.735	Pigmentation	[24,25,29,30]
109	rs183671	5:33964105	*SLC45A2*	T 0.04	-0.018	0.959	0.083	0.802	0.109	0.728	Pigmentation	[24,31]
110	rs13289	5:33986304	*SLC45A2*	C 0.43	0.216	0.109	0.146	0.248	0.162	0.177	Pigmentation	[32,33]
111	rs7702331	5:73255307	5q13.2	G 0.42	-0.036	0.776	0.061	0.611	0.068	0.551	Hair thickness	[20]
112	rs141577316	5:74129813	*ARHGEF28; ENC1*	T 0.01	0.230	0.632	0.254	0.580	0.248	0.566	Hair loss	[9]
113	rs335145	5:123108890	*PRDM6*	G 0.34	0.056	0.681	0.043	0.736	0.033	0.782	Hair loss	[8]
114	**rs929626**	**5:158883623**	***EBF1***	G 0.45	0.210	0.109	**0.291**	**0.020**	**0.275**	**0.022**	Hair loss	[4,5,21]
115	**rs1081073**	**5:158954504**	***EBF1***	A 0.48	0.201	0.118	**0.285**	**0.020**	**0.271**	**0.021**	Hair loss	[5,21]
116	**rs62389424**	**6:422631**	***IRF4***	A 0.07	0.206	0.418	0.402	0.084	**0.519**	**0.017**	Pigmentation	[34]
117	rs4959270	6:457748	*EXOC2*	A 0.44	0.118	0.380	0.173	0.169	0.216	0.070	Pigmentation	[25,29]
118	**rs1540771**	**6:466033**	***SEC5L1; IRF4***	T 0.45	0.183	0.171	0.216	0.085	**0.269**	**0.024**	Pigmentation	[25,29,35]
119	rs12197419	6:1708803	*GMDS*	C 0.24	-0.042	0.781	-0.067	0.641	-0.082	0.546	Hair thickness	[20]
120	rs4959410	6:6803205	6p25.1	T 0.37	-0.007	0.957	0.022	0.856	0.013	0.906	Hair loss	[5]
121	rs9348805	6:9334286	*OFCC1; SLC35B3*	G 0.40	0.004	0.974	0.045	0.717	0.030	0.799	Hair loss	[7]
122	rs1556547	6:10270144	*near OFCC1*	G 0.49	-0.181	0.164	-0.147	0.228	-0.166	0.151	Hair shape	[6]
123	rs7762265	6:21836604	*SOX4; NBAT1*	A 0.25	0.060	0.690	-0.011	0.941	-0.006	0.964	Hair loss	[9]
124	rs6901317	6:105588276	*PREP*	T 0.31	0.100	0.468	0.131	0.313	0.111	0.365	Hair thickness	[20]
125	rs12214131	6:105760046	6q21	A 0.32	0.046	0.741	0.034	0.798	-0.013	0.917	Hair loss	[8]
126	rs9388490	6:126383649	*CENPW*	T 0.43	-0.135	0.306	-0.094	0.451	-0.089	0.454	Hair loss	[7]
127	rs138345544	6:136204874	*RP1-143G15.4*	A 0.00	-0.113	0.930	0.096	0.929	0.318	0.726	Hair shape	[11]
128	rs9691699	7:493715	*PDGFA*	G 0.46	-0.002	0.989	0.026	0.829	0.025	0.819	Hair loss	[7,8]

129	**rs2110015**	**7:17773115**	***SNX13***	C 0.39	**-0.322**	**0.021**	**-0.260**	**0.040**	**-0.286**	**0.017**	Pigmentation	[36]
130	rs2073963	7:18838251	*HDAC9*	G 0.38	-0.179	0.164	-0.161	0.186	-0.204	0.079	Hair loss	[3-5]
131	rs756853	7:18850377	*HDAC9*	G 0.40	-0.141	0.269	-0.114	0.343	-0.170	0.141	Hair loss	[4,37]
132	rs6461387	7:18858682	*HDAC9*	A 0.39	-0.118	0.363	-0.093	0.444	-0.113	0.326	Hair loss	[4,9]
133	rs58788673	7:46864701	*EPS15P1*	CA 0.24	0.186	0.215	0.060	0.672	0.078	0.562	Hair loss	[8]
134	rs11766798	7:55056626	*EGFR*	A 0.26	0.147	0.314	0.194	0.155	0.095	0.468	Hair shape	[15]
135	rs939963	7:69122810	*RNU6; AUTS2*	G 0.45	-0.083	0.516	-0.070	0.556	-0.090	0.425	Hair loss	[7,8]
136	rs6945541	7:69146973	7q11.22	C 0.43	-0.137	0.296	-0.107	0.386	-0.124	0.291	Hair loss	[3-5,7]
137	rs10235789	7:84027484	*SEMA3A*	C 0.48	0.086	0.513	0.077	0.535	0.072	0.540	Pigmentation	[10]
138	rs2373391	7:88819986	*ZNF804B*	A 0.24	-0.049	0.753	-0.048	0.743	-0.072	0.601	Pigmentation	[38]
139	rs382270	7:102079556	*CUX1*	G 0.44	-0.068	0.614	-0.035	0.781	0.039	0.743	Hair shape	[15]
140	rs73199888	7:110116961	*EIF3IP1*	C 0.25	-0.003	0.983	0.004	0.977	0.033	0.804	Hair shape	[20]
141	rs117717824	7:114349147	*FOXP2*	T 0.01	1.219	0.124	1.180	0.058	0.929	0.109	Hair thickness	[20]
142	rs9719620	7:131309621	*MKLN1*	T 0.49	0.107	0.396	0.051	0.669	0.076	0.498	Hair loss	[8]
143	rs1454292	8:5068943	*PAICSP4; RSL24D1P7*	C 0.47	0.127	0.322	0.088	0.460	0.103	0.366	Hair shape	[11]
144	rs2047198	8:107950399	*RSPO2*	C 0.42	0.045	0.727	0.098	0.415	0.107	0.352	Hair loss	[7,8]
145	rs79206101	8:108133327	*near RSPO2*	T 0.00	0.590	0.521	0.451	0.542	0.903	0.173	Hair loss	[7]
146	rs9297550	8:116023559	*LINC00536*	G 0.36	0.048	0.723	0.089	0.488	0.105	0.382	Hair loss	[8]
147	rs7866411	9:2475084	*LOC101930053*	G 0.32	0.187	0.182	0.245	0.058	0.164	0.178	Pigmentation	[36]
148	rs12236890	9:3157137	9p24.2	C 0.45	-0.001	0.997	0.054	0.644	0.124	0.266	Hair thickness	[20]
149	rs1408799	9:12672097	*TYRP1*	T 0.31	-0.186	0.192	-0.144	0.281	-0.170	0.181	Pigmentation	[14,39-41]
150	rs683	9:12709305	*TYRP1*	C 0.35	-0.110	0.423	-0.102	0.434	-0.189	0.126	Pigmentation	[29,42]
151	rs10756819	9:16858086	*BNC2*	G 0.40	-0.051	0.708	-0.070	0.582	-0.077	0.521	Pigmentation	[22]
152	rs2153271	9:16864523	*BNC2*	C 0.47	-0.224	0.091	-0.224	0.067	-0.181	0.118	Pigmentation	[6,43]
153	rs12350739	9:16885019	*BNC2*	G 0.48	-0.131	0.326	-0.165	0.180	-0.124	0.288	Pigmentation	[44,45]
154	rs12686549	9:106867783	*ZNF462*	G 0.06	0.212	0.444	0.229	0.371	0.150	0.530	Hair loss	[8]
155	**rs2416699**	**9:119434462**	***BRINP1***	T 0.16	**-0.369**	**0.036**	**-0.363**	**0.030**	-0.275	0.083	Hair loss	[9]
156	rs62578126	9:126613059	*LMX1B*	T 0.31	0.035	0.803	0.058	0.658	0.003	0.981	Hair thickness	[20]
157	rs2784081	9:136897439	*TRAF2*	C 0.21	-0.015	0.922	-0.074	0.621	-0.040	0.776	Hair shape	[46]
158	rs4881147	10:3300582	*PFKP; PITRM1*	T 0.25	0.126	0.387	0.095	0.484	0.124	0.333	Hair shape	[20]
159	**rs2489250**	**10:8232904**	**10p14**	C 0.47	**0.310**	**0.019**	**0.315**	**0.010**	**0.274**	**0.019**	Hair shape	[1-2]
160	**rs17143387**	**10:8272081**	***GATA3***	G 0.15	**0.349**	**0.049**	0.247	0.128	0.176	0.254	Hair shape	[20]
161	rs1999874	10:8311138	*GATA3*	A 0.39	0.097	0.465	0.122	0.329	0.094	0.427	Hair shape	[1-2]
162	rs2807691	10:61157638	*RHOBTB1*	A 0.44	0.158	0.217	0.162	0.173	0.152	0.180	Hair loss	[8]

163	rs11593840	10:76436854	*LRMDA*	G 0.43	0.092	0.491	0.098	0.433	0.123	0.307	Hair loss	[8]
164	**rs2814331**	**10:86233584**	***GRID1***	C 0.07	0.483	0.056	**0.479**	**0.032**	**0.448**	**0.032**	Hair loss	[20]
165	rs4752566	10:121508117	*FGFR2*	T 0.47	-0.176	0.193	-0.070	0.579	-0.034	0.775	Hair shape	[15]
166	rs3781452	10:124666560	*FAM53B*	C 0.38	0.100	0.466	0.117	0.352	0.162	0.174	Hair loss	[7]
167	rs2219783	11:27389751	*LGR4*	G 0.06	0.003	0.991	-0.073	0.756	-0.250	0.272	Hair shape	[1-2]
168	rs78321654	11:27431772	*LGR4*	A 0.02	0.559	0.193	0.499	0.206	0.302	0.426	Hair loss	[7]
169	rs79811440	11:27432205	*LGR4*	C 0.02	0.533	0.211	0.478	0.223	0.285	0.450	Hair loss	[8]
170	rs11037975	11:44389312	*ALX4*	G 0.29	-0.097	0.494	-0.027	0.839	-0.020	0.871	Hair loss	[8]
171	rs35264875	11:69078931	*TPCN2*	T 0.24	-0.077	0.629	-0.198	0.186	-0.104	0.462	Pigmentation	[39]
172	rs3829241	11:69087895	*TPCN2*	A 0.38	-0.138	0.313	0.010	0.937	0.055	0.651	Pigmentation	[33,47]
173	rs72930659	11:69105375	*TPCN2*	T 0.19	-0.194	0.253	-0.237	0.140	-0.152	0.321	Pigmentation	[34]
174	rs10831496	11:88824823	*GRM5*	G 0.34	0.122	0.372	0.100	0.422	0.068	0.566	Pigmentation	[48,49]
175	rs1042602	11:89178528	*TYR*	A 0.30	0.037	0.788	-0.046	0.721	0.070	0.563	Pigmentation	[24,35]
176	rs1847134	11:89272085	*TYR*	C 0.28	0.049	0.735	0.092	0.497	0.080	0.537	Pigmentation	[6]
177	rs1393350	11:89277878	*TYR*	A 0.24	0.095	0.541	0.121	0.403	0.098	0.475	Pigmentation	[6,27,29,35,50]
178	rs1126809	11:89284793	*TYR*	A 0.25	0.099	0.519	0.100	0.478	0.098	0.466	Pigmentation	[14]
179	**rs4936890**	**11:124044035**	**11q24.2**	A 0.47	**-0.307**	**0.039**	**-0.335**	**0.017**	-0.249	0.060	Pigmentation	[51]
180	rs9668810	12:26273487	*SSPN*	T 0.24	0.168	0.275	0.238	0.097	0.251	0.063	Hair loss	[4,5,7]
181	rs7975017	12:26275860	*SSPN*	T 0.19	0.091	0.580	0.155	0.314	0.178	0.219	Hair loss	[5,21]
182	rs10843026	12:27940679	*KLHL42; PTHLH*	A 0.03	-0.256	0.521	-0.213	0.561	-0.173	0.619	Hair loss	[9]
183	rs7976269	12:29081094	*FAR2*	A 0.31	0.013	0.939	0.011	0.940	-0.011	0.938	Hair loss	[8]
184	rs12311316	12:52401803	*KRT82*	A 0.18	-0.157	0.339	-0.105	0.508	-0.050	0.743	Hair shape	[1]
185	rs585583	12:52535586	*KRT71*	T 0.36	0.125	0.350	0.116	0.358	0.128	0.285	Hair shape	[52]
186	**rs17662023**	**12:52540565**	***KRT71***	A 0.49	0.116	0.357	**0.242**	**0.043**	**0.224**	**0.049**	Hair shape	[52]
187	**rs10783518**	**12:52544713**	***KRT71***	C 0.49	0.117	0.352	**0.241**	**0.045**	**0.228**	**0.045**	Hair shape	[52]
188	**rs17730088**	**12:52552552**	***KRT71***	G 0.42	0.181	0.164	**0.265**	**0.032**	**0.244**	**0.037**	Hair shape	[52]
189	rs3912631	12:52569069	*KRT74*	C 0.29	0.006	0.966	0.017	0.899	0.005	0.967	Hair shape	[53]
190	rs670741	12:52571389	*KRT74*	T 0.27	-0.072	0.618	-0.038	0.778	-0.021	0.873	Hair shape	[53]
191	rs673449	12:52571977	*KRT74*	C 0.27	-0.072	0.624	-0.033	0.811	-0.019	0.881	Hair shape	[53]
192	rs11170678	12:53760390	*HOX13*	G 0.32	-0.117	0.380	-0.067	0.596	-0.052	0.659	Hair shape	[1-2]
193	rs10777129	12:88567936	*KITLG*	A 0.06	-0.481	0.084	-0.241	0.349	-0.324	0.193	Pigmentation	[54]
194	rs12821256	12:88934558	*KITLG*	C 0.09	-0.023	0.922	0.171	0.419	0.204	0.309	Pigmentation	[14,34,35]
195	rs9568036	13:48397800	*P2RY5*	G 0.44	0.059	0.649	0.025	0.835	0.059	0.607	Hair shape	[55]
196	rs198607	13:48405031	*P2RY5*	C 0.29	-0.183	0.199	-0.170	0.209	-0.121	0.350	Hair shape	[55]

197	**rs2227311**	**13:48412896**	***P2RY5***	G 0.13	**0.438**	**0.023**	**0.377**	**0.035**	**0.411**	**0.015**	Hair shape	[55]
198	rs9535032	13:48438129	*P2RY5*	G 0.31	-0.143	0.308	-0.153	0.247	-0.136	0.282	Hair shape	[55]
199	rs975739	13:77807011	*EDNRB*	G 0.40	-0.147	0.268	-0.133	0.282	-0.055	0.641	Pigmentation	[14]
200	rs3782974	13:94440642	*DCT*	T 0.13	-0.027	0.883	-0.073	0.674	-0.085	0.608	Pigmentation	[51,52]
201	rs417054	14:30078929	*PRKD1*	A 0.19	-0.270	0.109	-0.225	0.150	-0.230	0.118	Hair loss	[8]
202	rs8017455	14:81109110	*TSHR*	C 0.22	0.048	0.759	-0.007	0.959	-0.036	0.798	Hair shape	[11]
203	rs4900109	14:92297047	*SLC32A4*	T 0.42	-0.067	0.614	0.013	0.916	-0.005	0.966	Pigmentation	[10]
204	rs12896399	14:92307319	*SLC24A4*	T 0.41	-0.083	0.532	-0.001	0.992	-0.021	0.858	Pigmentation	[6,14,27,35,40]
205	rs4904868	14:92314657	14q32.12	T 0.48	0.146	0.275	0.021	0.864	0.031	0.793	Pigmentation	[6,29,57]
206	rs8014907	14:92333660	*SLC24A4*	T 0.17	-0.053	0.766	-0.048	0.775	-0.024	0.882	Pigmentation	[34]
207	rs2402130	14:92334859	*SLC24A4*	G 0.17	-0.073	0.681	-0.068	0.683	-0.041	0.799	Pigmentation	[29,42]
208	rs17128291	14:92416482	*SLC24A4*	G 0.15	0.032	0.859	-0.019	0.909	-0.071	0.655	Pigmentation	[31,51]
209	rs3742377	14:99699891	*CYP46A1*	A 0.16	0.175	0.288	0.016	0.918	0.042	0.775	Hair shape	[20]
210	rs142826872	15:24167222	*PWRN2*	T 0.02	0.462	0.322	0.368	0.395	0.334	0.408	Hair shape	[11]
211	rs4906800	15:25875535	*MIR4715*	A 0.06	-0.366	0.166	-0.186	0.466	-0.268	0.275	Hair loss	[9]
212	rs1545397	15:27942626	*OCA2*	T 0.08	0.015	0.949	-0.081	0.718	0.010	0.963	Pigmentation	[51,58,59]
213	**rs7173419**	**15:27951675**	***OCA2***	T 0.24	**0.330**	**0.029**	0.265	0.056	**0.290**	**0.027**	Pigmentation	[14]
214	rs74653330	15:27983407	*OCA2*	T 0.01	-0.786	0.298	-1.063	0.223	-1.130	0.188	Pigmentation	[47]
215	rs121918166	15:27985101	*OCA2*	T 0.00	-9.942	0.985	-18.835	-	-18.841	-	Pigmentation	[47]
216	**rs1800407**	**15:27985172**	***OCA2***	T 0.06	**0.676**	**0.015**	**0.530**	**0.034**	**0.472**	**0.044**	Pigmentation	[27,28,30,40,41,50,60,61,62]
217	rs1800401	15:28014907	*OCA2*	A 0.03	-0.006	0.987	-0.089	0.780	-0.076	0.806	Pigmentation	[60]
218	rs12441727	15:28026629	*OCA2*	A 0.12	-0.234	0.260	-0.219	0.264	-0.291	0.119	Pigmentation	[27]
219	rs4778232	15:28036619	*OCA2*	T 0.21	-0.058	0.722	-0.018	0.906	-0.066	0.651	Pigmentation	[30,63]
220	rs1448485	15:28037595	*OCA2*	T 0.13	-0.139	0.491	-0.140	0.463	-0.188	0.302	Pigmentation	[51]
221	rs1448484	15:28038295	*OCA2*	G 0.00	0.063	0.957	0.024	0.982	0.375	0.694	Pigmentation	[27,60,63]
222	rs16950821	15:28038361	*OCA2*	A 0.11	-0.206	0.326	-0.216	0.273	-0.253	0.175	Pigmentation	[29]
223	rs8024968	15:28038543	*OCA2*	T 0.11	-0.206	0.326	-0.216	0.273	-0.253	0.175	Pigmentation	[30,63]
224	rs1470608	15:28042975	*OCA2*	T 0.13	-0.115	0.557	-0.127	0.496	-0.169	0.343	Pigmentation	[6,54]
225	rs1375164	15:28046666	*OCA2*	T 0.20	-0.032	0.844	-0.010	0.947	-0.041	0.777	Pigmentation	[30,56,60]
226	rs7174027	15:28083619	*OCA2*	A 0.11	-0.006	0.977	-0.011	0.952	-0.079	0.664	Pigmentation	[25,29,54,57]
227	rs4778138	15:28090674	*OCA2*	G 0.15	0.090	0.617	0.044	0.795	0.010	0.951	Pigmentation	[6,29,30,60,63]
228	rs4778241	15:28093567	*OCA2*	A 0.19	0.160	0.337	0.136	0.382	0.043	0.771	Pigmentation	[25,29,60,61-63]
229	rs7495174	15:28099092	*OCA2*	G 0.05	0.522	0.083	0.535	0.050	0.439	0.096	Pigmentation	[25,34,57,61,63]

230	rs1129038	15:28111713	*HERC2*	C 0.18	0.238	0.185	0.252	0.132	0.207	0.195	Pigmentation	[30,47,54,61,62]
231	rs12913832	15:28120472	*HERC2*	A 0.18	0.247	0.169	0.247	0.140	0.207	0.195	Pigmentation	[6,14,25,27,29,45,47,51,61,62,64]
232	rs7183877	15:28120587	*HERC2*	A 0.06	0.197	0.492	0.156	0.541	0.115	0.634	Pigmentation	[16,30,54,61,62]
233	rs11636232	15:28141480	*HERC2*	T 0.42	-0.013	0.922	-0.036	0.774	-0.043	0.718	Pigmentation	[25,30,54]
234	**rs2238289**	**15:28208069**	***HERC2***	G 0.10	0.415	0.063	**0.416**	**0.039**	0.312	0.103	Pigmentation	[54]
235	**rs8182028**	**15:28222789**	***HERC2***	C 0.04	0.626	0.057	**0.734**	**0.015**	**0.609**	**0.036**	Pigmentation	[27,51]
236	**rs6497292**	**15:28251049**	***HERC2***	G 0.04	0.642	0.050	**0.734**	**0.014**	**0.589**	**0.040**	Pigmentation	[63]
237	**rs16950941**	**15:28257598**	***HERC2***	A 0.04	0.626	0.057	**0.734**	**0.015**	**0.609**	**0.036**	Pigmentation	[63]
238	**rs79097182**	**15:28266851**	***HERC2***	T 0.04	0.567	0.075	**0.681**	**0.020**	**0.566**	**0.046**	Pigmentation	[34]
239	rs916977	15:28268218	*HERC2*	T 0.13	0.302	0.126	0.289	0.113	0.224	0.200	Pigmentation	[30,63]
240	rs1667394	15:28285036	*HERC2*	C 0.13	0.313	0.112	0.341	0.060	0.270	0.119	Pigmentation	[30,35,61,63]
241	**rs12592730**	**15:28285213**	***HERC2***	A 0.04	0.626	0.057	**0.734**	**0.015**	**0.609**	**0.036**	Pigmentation	[57,63]
242	rs4424881	15:28969513	*APBA2*	T 0.13	0.045	0.823	-0.015	0.937	-0.017	0.922	Pigmentation	[49,65]
243	rs2924567	15:47976289	*SLC24A5*	T 0.33	-0.083	0.561	-0.022	0.869	-0.033	0.788	Pigmentation	[24,31]
244	rs1834640	15:48099968	*SLC24A5*	G 0.01	-1.724	0.050	-1.597	0.065	-1.361	0.106	Pigmentation	[24]
245	rs1426654	15:48134287	*SLC24A5*	G 0.01	-1.533	0.088	-1.509	0.088	-1.249	0.146	Pigmentation	[24,33,42,51]
246	rs7173477	15:57258766	*TCF12*	C 0.18	0.143	0.403	-0.031	0.844	-0.068	0.652	Hair loss	[9]
247	rs17270216	15:60544595	*RORA*	G 0.23	0.069	0.640	0.061	0.660	0.066	0.611	Hair loss	[9]
248	rs11630290	15:63623587	*HERC1*	T 0.23	-0.140	0.357	-0.126	0.374	-0.140	0.296	Pigmentation	[10]
249	rs12902958	15:69747915	*DRAIC*	A 0.09	0.099	0.665	0.189	0.381	0.150	0.462	Hair loss	[8]
250	rs8033595	15:90540121	*CRTC3; IQGAP1*	G 0.43	-0.204	0.120	-0.100	0.421	-0.063	0.598	Hair loss	[8]
251	rs11150606	16:31087690	*PRSS53*	C 0.03	-0.250	0.497	-0.249	0.487	-0.321	0.357	Hair shape	[20]
252	rs12597422	16:53853826	*FTO*	G 0.25	-0.176	0.234	-0.175	0.207	-0.130	0.323	Hair thickness	[20]
253	**rs2353688**	**16:86329448**	**16q24.1**	C 0.01	**-1.462**	**0.019**	**-1.386**	**0.022**	**-1.470**	**0.013**	Pigmentation	[34]
254	rs3114908	16:89317317	*ANKRD11*	T 0.29	0.091	0.529	0.024	0.857	-0.067	0.601	Pigmentation	[51,66]
255	**rs164741**	**16:89625890**	***DPEP1***	A 0.25	**0.428**	**0.004**	**0.364**	**0.007**	**0.260**	**0.041**	Pigmentation	[25,48,51]
256	rs12931267	16:89752324	*FANCA*	G 0.03	0.367	0.319	0.345	0.302	0.258	0.416	Pigmentation	[6,31,57]
257	rs1805005	16:89919436	*MC1R*	T 0.07	0.003	0.990	-0.112	0.650	-0.115	0.618	Pigmentation	[24,29,31,42,47,67]
258	rs1805006	16:89919510	*MC1R*	A 0.00	-0.961	0.477	-1.092	0.413	-1.181	0.357	Pigmentation	[24,47,67]
259	rs2228479	16:89919532	*MC1R*	A 0.11	-0.372	0.092	-0.352	0.088	-0.345	0.077	Pigmentation	[24,29,31,42,54]
260	rs11547464	16:89919683	*MC1R*	A 0.00	-14.249	0.978	-23.159		-23.717		Pigmentation	[6,29,47]

261	rs1805007	16:89919709	*MC1R*	T 0.03	0.628	0.090	0.543	0.100	0.424	0.177	Pigmentation	[6,14,24,29,31,35,42,45,47,54,67]
262	rs1110400	16:89919722	*MC1R*	C 0.02	-0.538	0.333	-0.407	0.432	-0.497	0.328	Pigmentation	[24,29,31,47]
263	rs1805008	16:89919736	*MC1R*	T 0.08	-0.108	0.663	-0.137	0.564	-0.250	0.268	Pigmentation	[24,29,31,35,42,47,54]
264	rs885479	16:89919746	*MC1R*	A 0.04	0.174	0.621	0.293	0.361	0.223	0.460	Pigmentation	[24,29,54]
265	rs1805009	16:89920138	*MC1R*	C 0.00	0.714	0.700	0.254	0.874	1.050	0.415	Pigmentation	[6,24,29,31,67]
266	rs8049897	16:89957794	*DEF8*	A 0.12	-0.230	0.273	-0.284	0.145	-0.296	0.108	Pigmentation	[6,25,48,51]
267	**rs8051733**	**16:89957798**	***DEF8***	G 0.29	-0.158	0.270	-0.192	0.147	**-0.256**	**0.041**	Pigmentation	[51,68]
268	rs4268748	16:89960104	*DEF8*	C 0.26	-0.123	0.413	-0.189	0.174	-0.246	0.062	Pigmentation	[31,51,68]
269	rs11076649	16:89992928	*AFG3L1P*	G 0.12	-0.166	0.419	-0.233	0.220	-0.214	0.235	Pigmentation	[51]
270	rs333113	17:4497061	*SPNS2*	C 0.24	-0.031	0.837	-0.043	0.760	-0.164	0.226	Pigmentation	[51]
271	rs72809171	17:12553762	*LINC00670*	A 0.04	0.161	0.600	-0.016	0.956	0.218	0.416	Hair loss	[8]
272	rs143290289	17:41060725	*KRTAP2-3*	A 0.09	-0.044	0.833	-0.134	0.503	-0.028	0.883	Hair shape	[1-2]
273	rs12373124	17:45846853	*IMP5*	C 0.16	0.118	0.504	0.143	0.378	0.231	0.129	Hair loss	[4,5,21]
274	rs112385572	17:45988806	*MAPT*	G 0.16	0.125	0.482	0.149	0.362	0.237	0.121	Hair loss	[7,8]
275	rs538628	17:46709947	*ARL17A*	C 0.14	0.059	0.751	0.079	0.646	0.190	0.237	Hair loss	[7]
276	rs17833789	17:57153267	*MSI2*	A 0.48	0.177	0.179	0.113	0.359	0.107	0.356	Hair loss	[8]
277	rs79806428	17:66282767	*APOH;PRKCA*	T 0.06	-0.332	0.251	-0.317	0.252	-0.246	0.346	Hair loss	[9]
278	rs9894429	17:81629785	*NPLOC4*	T 0.43	-0.005	0.970	-0.075	0.551	-0.068	0.566	Pigmentation	[16,69]
279	**rs29073**	**18:9971793**	***APCDD1***	C 0.41	-0.163	0.200	**-0.254**	**0.035**	**-0.254**	**0.028**	Hair loss	[8]
280	rs4800451	18:23136841	*CABLES1; MIR4741*	C 0.28	0.121	0.399	0.063	0.639	0.087	0.499	Hair shape	[20]
281	rs10502861	18:45220183	*SLC14A2*	T 0.27	0.102	0.481	0.055	0.684	0.006	0.965	Hair loss	[3-5]
282	rs7226979	18:63257737	*BCL2*	C 0.48	0.025	0.845	0.041	0.736	0.027	0.814	Hair loss	[8]
283	rs17318596	19:41431190	*B3GNT8*	A 0.39	-0.168	0.211	-0.104	0.408	-0.141	0.240	Hair loss	[9]
284	rs4380312	20:2258361	*TGM3*	T 0.04	0.139	0.685	0.195	0.533	0.200	0.501	Hair shape	[1]
285	rs6132532	20:2334897	*TGM3*	G 0.02	-0.028	0.951	0.026	0.952	0.064	0.875	Hair shape	[15]
286	rs261360	20:5057128	*RPS21P7; TMEM230*	A 0.44	-0.166	0.201	-0.238	0.051	-0.177	0.123	Hair shape	[11]
287	rs733626	20:17704704	*BANF2*	G 0.17	0.084	0.610	0.115	0.456	0.117	0.416	Hair loss	[9]
288	rs2424371	20:21458745	*NKX2*	G 0.19	-0.014	0.933	-0.075	0.622	-0.112	0.436	Hair loss	[9]
289	rs2180439	20:21872462	20p11.22	C 0.43	-0.026	0.836	-0.080	0.505	-0.007	0.950	Hair loss	[4,37,70]
290	rs1998076	20:21899407	20p11.22	A 0.43	-0.034	0.787	-0.088	0.467	-0.013	0.906	Hair loss	[4,70]
291	rs4544515	20:21904673	20p11.22	C 0.44	0.004	0.972	-0.040	0.738	0.009	0.937	Hair loss	[7]

292	rs201593	20:22053181	*LOC100270679*	G 0.45	-0.094	0.458	-0.110	0.363	-0.065	0.574	Hair loss	[7]
293	rs6047844	20:22056937	*LINC01432*	C 0.45	-0.094	0.458	-0.110	0.363	-0.065	0.574	Hair loss	[3-5]
294	rs913063	20:22061780	*LINC01432*	C 0.46	-0.111	0.384	-0.118	0.332	-0.071	0.545	Hair loss	[4]
295	rs1160312	20:22069865	*LINC01432*	G 0.46	-0.111	0.384	-0.118	0.332	-0.071	0.545	Hair loss	[4,71]
296	rs6113491	20:22076777	20p11.22	C 0.46	-0.092	0.469	-0.100	0.412	-0.057	0.628	Hair loss	[4]
297	rs804520	20:22138503	20p11.22	A 0.44	-0.085	0.502	-0.110	0.366	-0.086	0.459	Hair loss	[3,5]
298	rs6113548	20:22156201	20p11.22	C 0.37	-0.092	0.484	-0.143	0.253	-0.109	0.359	Hair loss	[7]
299	rs6059655	20:34077942	*RALY*	A 0.04	0.016	0.964	-0.038	0.904	-0.168	0.574	Pigmentation	[31,72]
300	rs4911414	20:34141638	*ASIP*	T 0.33	0.050	0.724	0.101	0.431	0.096	0.426	Pigmentation	[14,31]
301	rs1015362	20:34150806	*ASIP*	T 0.31	0.001	0.996	0.088	0.505	0.102	0.412	Pigmentation	[39,41,48]
302	rs6119471	20:34197406	*ASIP*	G 0.00	-15.815	0.980	-24.509		-24.443		Pigmentation	[73]
303	rs2424984	20:34262569	*ASIP*	C 0.14	-0.022	0.905	0.062	0.719	0.014	0.932	Pigmentation	[42]
304	rs6058017	20:34269192	*ASIP*	G 0.14	-0.022	0.905	0.062	0.719	0.014	0.932	Pigmentation	[24,29,42,41,48]
305	rs2378249	20:34630286	*ASIP*	G 0.14	0.018	0.925	0.027	0.876	-0.047	0.777	Pigmentation	[29]
306	rs4911442	20:34767243	*NCOA6*	G 0.08	-0.086	0.720	-0.030	0.891	-0.066	0.746	Pigmentation	[6,43,74]
307	rs1885120	20:34989186	*MYH7B*	C 0.04	-0.135	0.700	-0.055	0.858	-0.147	0.609	Pigmentation	[31,51,66]
308	rs6072223	20:40992207	*MAFB; TOP1*	A 0.19	-0.133	0.429	-0.104	0.507	-0.055	0.711	Hair loss	[9]
309	rs17265513	20:41203988	*ZHX3*	C 0.22	-0.027	0.857	-0.033	0.816	-0.021	0.876	Hair loss	[8]
310	rs985546	20:56859627	*TFAP2C*	C 0.18	0.013	0.939	0.002	0.991	0.002	0.991	Hair loss	[8]
311	**rs310642**	**20:63530645**	***PTK6***	C 0.04	-0.595	0.091	**-0.726**	**0.035**	**-0.662**	**0.042**	Hair shape	[1-2]
312	**rs68088846**	**21:34835870**	***RUNX1***	A 0.20	**0.311**	**0.049**	0.240	0.101	0.224	0.110	Hair loss	[8]
313	rs7277820	21:37208008	*DSCR9*	G 0.48	0.015	0.905	0.109	0.359	0.110	0.332	Pigmentation	[16]
314	rs2256843	21:44754576	21q22.3	T 0.38	-0.224	0.091	-0.155	0.206	-0.191	0.103	Hair loss	[8]
315	rs5934505	X:8945785	*FAM9B*	C 0.21	0.092	0.442	0.050	0.647	0.038	0.717	Hair loss	[8]
316	rs147829649	X:57979046	Xp11.21	G 0.04	-0.101	0.691	-0.162	0.505	-0.199	0.383	Hair loss	[7]
317	rs6624142	X:65185289	*ZC4H2*	C 0.28	0.049	0.655	-0.003	0.976	-0.051	0.590	Hair loss	[7]
318	rs147154263	X:65863405	*VSIG4*	T 0.03	-0.306	0.261	-0.336	0.224	-0.316	0.232	Hair loss	[7]
319	rs145867342	X:66322114	*HEPH; VSIG4*	T 0.07	0.110	0.557	-0.027	0.880	-0.066	0.690	Hair loss	[7]
320	rs73221556	X:66713443	*EDA2R; HEPH*	A 0.15	-0.004	0.976	-0.057	0.645	-0.111	0.337	Hair loss	[7]
321	rs1041668	X:66866114	Xq12	G 0.14	-0.035	0.798	-0.077	0.538	-0.135	0.248	Hair loss	[4,75]
322	rs471205	X:67018475	Xq12	T 0.23	-0.035	0.758	-0.081	0.445	-0.123	0.220	Hair loss	[3]
323	rs1511061	X:67096282	Xq12	C 0.15	0.001	0.995	-0.066	0.589	-0.132	0.251	Hair loss	[3,5]
324	rs17216820	X:67117703	Xq12	T 0.08	-0.282	0.138	-0.262	0.152	-0.211	0.235	Hair loss	[7]
325	rs5919324	X:67210798	Xq12	G 0.15	0.022	0.868	-0.048	0.693	-0.116	0.310	Hair loss	[4,76]

326	rs6625150	X:67229126	Xq12	A 0.17	0.054	0.668	-0.018	0.880	-0.102	0.353	Hair loss	[4]
327	rs6625163	X:67291142	Xq12	G 0.19	0.018	0.881	-0.070	0.533	-0.135	0.202	Hair loss	[4,71]
328	rs2497938	X:67343176	Xq12	C 0.18	0.031	0.802	-0.059	0.601	-0.124	0.247	Hair loss	[4,5,75]
329	rs962458	X:67526122	Xq12	G 0.08	-0.122	0.504	-0.207	0.220	-0.179	0.247	Hair loss	[4]
330	rs12007229	X:67528513	Xq12	A 0.09	0.157	0.359	0.082	0.601	-0.025	0.864	Hair loss	[4]
331	rs6152	X:67545785	*AR*	A 0.17	0.027	0.831	-0.059	0.616	-0.107	0.333	Hair loss	[4,75,77]
332	rs5919427	X:67783742	*OPHN1*	C 0.19	0.103	0.369	0.043	0.683	-0.015	0.879	Hair loss	[7]
333	rs148652266	X:67919221	Xq13.1	A 0.03	0.059	0.837	-0.001	0.996	-0.178	0.448	Hair loss	[7]
334	rs7061504	X:68143959	*OPHN1*	G 0.09	0.265	0.112	0.262	0.081	0.157	0.272	Hair loss	[7]
335	rs140488081	X:68276160	*OPHN1*	T 0.03	-0.441	0.124	-0.475	0.096	-0.452	0.105	Hair loss	[7]
336	rs4829906	X:137873245	*ZIC3*	A 0.35	-0.126	0.218	-0.111	0.244	-0.106	0.238	Hair loss	[9]

Significant results are marked with bold.

BLR, binomial logistic regression; MLR3, multinomial ordinal logistic regression for 3 hair greying categories; MLR6, multinomial ordinal logistic regression for 6 hair greying categories; MA, minor allele; fMA, frequency of minor allele.

*Results adjusted for age and sex (in case of pigmentation-linked SNPs adjustment for hair colour was also applied)

References

1. F. Liu, Y. Chen, G. Zhu, P.G. Hysi, S. Wu, et al., Meta-analysis of genome-wide association studies identifies 8 novel loci involved in shape variation of human head hair, Hum. Mol. Genet. 27 (2018) 559–575.
2. E. Pośpiech, Y. Chen, M. Kukla-Bartoszek, K. Breslin K., A. Aliferi, et al., Towards broadening Forensic DNA Phenotyping beyond pigmentation: Improving the prediction of head hair shape from DNA. Forensic Sci Int Genet. 37 (2018) 241-251.
3. R. Li, F.F. Brockschmidt, A.K. Kiefer, H. Stefansson, D.R. Nyholt, et al., Six novel susceptibility loci for early-onset androgenetic alopecia and their unexpected association with common diseases, PLoS Genet. 8 (2012) e1002746. doi: 10.1371/journal.pgen.1002746.
4. Marcińska, E. Pośpiech, S. Abidi, J. Dyrberg Andersen, M. van den Berge, et al., Evaluation of DNA variants associated with androgenetic alopecia and their potential to predict male pattern baldness, PLoS One 10 (2015) e0127852.
5. F. Liu, M.A. Hamer, S. Heilmann, C. Herold, S. Moebus, et al., Prediction of male pattern baldness from genotypes, Eur. J. Hum. Genet. 24 (2016) 895–902.
6. N. Eriksson, J.M. Macpherson, J.Y. Tung, L.S. Hon, B. Naughton, et al., Web-based, participant-driven studies yield novel genetic associations for common traits, PLoS Genet. 6 (2010) e1000993.

7. S.P. Hagenaars, W.D. Hill, S.E. Harris, S.J. Ritchie, G. Davies, et al., Genetic prediction of male pattern baldness, PLoS Genet. 13 (2017) e1006594.
8. N. Pirastu, P.K. Joshi, P.S. de Vries, M.C. Cornelis, P.M. McKeigue, et al., GWAS for male-pattern baldness identifies 71 susceptibility loci explaining 38% of the risk, Nat. Commun. 8 (2017) 1584.
9. S. Heilmann-Heimbach, C. Herold, L.M. Hochfeld, A.M. Hillmer, D.R. Nyholt et al., Meta-analysis identifies novel risk loci and yields systematic insights into the biology of male-pattern baldness, Nat Commun. 8 (2017) 14694.
10. M. Larsson, D.L. Duffy, G. Zhu, J.Z. Liu, S. Macgregor, et al., GWAS findings for human iris patterns: associations with variants in genes that influence normal neuronal pattern development. Am J Hum Genet. 89 (2011) 334-43.
11. S.E. Medland, D.R. Nyholt, J.N. Painter, B.P. McEvoy, A.F. McRae, et al., Common variants in the trichohyalin gene are associated with straight hair in Europeans, Am. J. Hum. Genet. 85 (2009) 750–755.
12. E. Pośpiech, J. Karłowska-Pik, M. Marcińska, S. Abidi, J.D. Andersen, et al., Evaluation of the predictive capacity of DNA variants associated with straight hair in Europeans, Forensic Sci. Int. Genet. 19 (2015) 280–288.
13. E. Pośpiech, S.D. Lee, M. Kukla-Bartoszek, J. Karłowska-Pik, A. Woźniak, M. Boroń, M. Zubańska, A. Bronikowska, S.R. Hong, J.H. Lee, A. Wojas-Pelc, H.Y. Lee, M. Spólnicka, W. Branicki, Variation in the RPTN gene may facilitate straight hair formation in Europeans and East Asians, J. Dermatol. Sci. S0923-1811 (18) (2018) 30246–30249.
14. M. Zhang, F. Song, L. Liang, H. Nan, J. Zhang, et al., Genome-wide association studies identify several new loci associated with pigmentation traits and skin cancer risk in European Americans. Hum Mol Genet. 22 (2013) 2948-59.
15. A. Fujimoto, N. Nishida, R. Kimura, T. Miyagawa, R. Yuliwulandari et al., FGFR2 is associated with hair thickness in Asian populations. J Hum Genet. 54 (2009) 461-5.
16. F. Liu, A. Wollstein, P.G. Hysi, G.A. Ankra-Badu, T.D. Spector, et al., Digital quantification of human eye color highlights genetic association of three new loci. PLoS Genet. 6 (2010) e1000934.
17. A. Fujimoto, R. Kimura, J. Ohashi, K. Omi, R. Yuliwulandari, L. Batubara, M.S. Mustofa, et al., A scan for genetic determinants of human hair morphology: EDAR is associated with Asian hair thickness, Hum. Mol. Genet. 17 (2008) 835–843.
18. Y. Tan, K. Yang, P.C. Tang, L. Sabeti, Jin, et al., The adaptive variant EDARV370A is associated with straight hair in East Asians, Hum. Genet. 132 (2013) 1187–1191.
19. S. Wu, J. Tan, Y. Yang, Q. Peng, M. Zhang, et al., Genome-wide scans reveal variants at EDAR predominantly affecting hair straightness in Han Chinese and Uyghur populations, Hum. Genet. 135 (2016) 1279–1286.
20. K. Adhikari, T. Fontanil, S. Cal, J. Mendoza-Revilla, M. Fuentes-Guajardo, et al., A genome-wide association scan in admixed Latin Americans identifies loci influencing facial and scalp hair features, Nat. Commun. 7 (2016) 10815.

21. S. Heilmann, A.K. Kiefer, N. Fricker, D. Drichel, A.M. Hillmer, C. Herold, et al., Androgenetic alopecia: identification of four genetic risk loci and evidence for the contribution of WNT signaling to its etiology, J. Invest. Dermatol.133 (2013) 1489–1496.
22. L.C. Jacobs, A. Wollstein, O. Lao, A. Hofman, C.C. Klaver, et al., Comprehensive candidate gene study highlights UGT1A and BNC2 as new genes determining continuous skin color variation in Europeans. Hum Genet. 132 (2013) 147-58.
23. Y. Shimomura, M. Wajid, L. Petukhova, L. Shapiro, A.M. Christiano, Mutations in the lipase H gene underlie autosomal recessive woolly hair/hypotrichosis, J. Invest. Dermatol. 129 (2009) 622–628.
24. R.P. Stokowski, P.V. Pant, T. Dadd, A. Fereday, D.A. Hinds, et al., A genomewide association study of skin pigmentation in a South Asian population. Am J Hum Genet. 81 (2007) 1119-32.
25. J. Han, P. Kraft, H. Nan, Q. Guo, C. Chen, et al., A genome-wide association study identifies novel alleles associated with hair color and skin pigmentation, PLoS Genet. 4 (2008) e1000074.
26. W. Branicki, U. Brudnik, J. Draus-Barini, T. Kupiec, A. Wojas-Pelc, Association of the SLC45A2 gene with physiological human hair colour variation. J Hum Genet. 53 (2008) 966-71.
27. F. Liu, K. van Duijn, J.R. Vingerling, A. Hofman, A.G. Uitterlinden, et al., Eye color and the prediction of complex phenotypes from genotypes, Curr. Biol. 19 (2009) R192–R193.
28. J. Mengel-From, C. Børsting, J.J. Sanchez, H. Eiberg, N. Morling, Human eye colour and HERC2, OCA2 and MATP, Forensic. Sci. Int. Genet. 4 (2010) 323–328.
29. W. Branicki, F. Liu, K. van Duijn, J. Draus-Barini, E. Pos´piech, et al., Model-based prediction of human hair color using DNA variants, Hum. Genet. 129 (2011) 443–454.
30. Y. Ruiz, C. Phillips, A. Gomez-Tato, J. Alvarez-Dios, M. de Cal Casares, et al., Further development of forensic eye color predictive tests, Forensic Sci. Int. Genet. 7 (2013) 28–40.
31. F. Liu, M. Visser, D.L. Duffy, P.G. Hysi, L.C. Jacobs, O. Lao, et al., Genetics of skin color variation in Europeans: genome-wide association studies with functional follow-up. Hum Genet. 134 (2015) 823-35.
32. J. Graf, J. Voisey, I. Hughes, A. van Daal, Promoter polymorphisms in the MATP (SLC45A2) gene are associated with normal human skin color variation. Hum Mutat. 28 (2007) 710-7.
33. O. Maroñas, J. Söchtig, Y. Ruiz, C. Phillips, A. Carracedo, et al., The genetics of skin, hair, and eye color variation and its relevance to forensic pigmentation predictive tests. Forensic Sci Rev. 27 (2015) 13-40.
34. B.D. Lin, H. Mbarek, G. Willemsen, C.V. Dolan, I.O. Fedko, et al., Heritability and Genome-Wide Association Studies for Hair Color in a Dutch Twin Family Based Sample. Genes (Basel). 6 (2015) 559-76.
35. P. Sulem, D.F. Gudbjartsson, S.N. Stacey, A. Helgason, T. Rafnar, et al., Genetic determinants of hair, eye and skin pigmentation in Europeans, Nat. Genet. 39 (2007) 1443–1452.

36. A.R. Martin, M. Lin, J.M. Granka, J.W. Myrick, X. Liu, et al., An Unexpectedly Complex Architecture for Skin Pigmentation in Africans. Cell. 30 (2017) 1340-1353.e14.
37. F.F. Brockschmidt, S. Heilmann, J.A. Ellis, S. Eigelshoven, S. Hanneken, et al. Susceptibility variants on chromosome 7p21.1 suggest HDAC9 as a new candidate gene for male-pattern baldness. Br J Dermatol. 165 (2011) 1293–1302.
38. L. Rawofi, M. Edwards, S. Krithika, P. Le, D. Cha, et al., Genome-wide association study of pigmentary traits (skin and iris color) in individuals of East Asian ancestry. PeerJ. 5 (2017) e3951.
39. P. Sulem, D.F. Gudbjartsson, S.N. Stacey, A. Helgason, T. Rafnar, et al., Two newly identified genetic determinants of pigmentation in Europeans, Nat. Genet. 40 (2008) 835–837.
40. E. Pośpiech, J. Draus-Barini, T. Kupiec, A. Wojas-Pelc, W. Branicki, Gene–gene interactions contribute to eye colour variation in humans, J. Hum. Genet. 56 (2011) 447–455.
41. E. Pośpiech, A. Wojas-Pelc, S. Walsh, F. Liu, H. Maeda, et al., The common occurrence of epistasis in the determination of human pigmentation and its impact on DNA-based pigmentation phenotype prediction. Forensic Sci Int Genet. 11 (2014) 64-72.
42. R.K. Valenzuela, M.S. Henderson, M.H. Walsh, N.A. Garrison, J.T. Kelch, et al., Predicting phenotype from genotype: normal pigmentation, J. Forensic Sci. 55 (2010) 315–322.
43. B. Hernando, M.V. Ibañez, J.A. Deserio-Cuesta, R. Soria-Navarro, I. Vilar-Sastre, et al., Genetic determinants of freckle occurrence in the Spanish population: Towards ephelides prediction from human DNA samples. Forensic Sci Int Genet. 33 (2018) 33:38-47.
44. M. Visser, R.J. Palstra, M. Kayser, Human skin color is influenced by an intergenic DNA polymorphism regulating transcription of the nearby BNC2 pigmentation gene. Hum Mol Genet. 23 (2014) 5750-62.
45. P.G. Hysi, A.M. Valdes, F. Liu, N.A. Furlotte, D.M. Evans, et al., Genome-wide association meta-analysis of individuals of European ancestry identifies new loci explaining a substantial fraction of hair color variation and heritability. Nat Genet. 50 (2018) 652-656.
46. G. Lubke, C. Laurin, R. Walters, N. Eriksson, P. Hysi, et al., Gradient boosting as a SNP filter: an evaluation using simulated and hair morphology data, J. Data Mining Genomics Proteomics 4 (2013),.
47. M.D. Morgan, E. Pairo-Castineira, K. Rawlik, O. Canela-Xandri, J. Rees, et al., Genome-wide study of hair colour in UK Biobank explains most of the SNP heritability. Nat Commun. 9 (2018) 5271.
48. H. Nan, P. Kraft, A.A. Qureshi, Q. Guo, C. Chen, et al., Genome-wide association study of tanning phenotype in a population of European ancestry. J Invest Dermatol. 129 (2009) 2250-7.
49. S. Beleza, N.A. Johnson, S. Candille, D.M. Absher, M.A. Coram, et al., Genetic architecture of skin and eye color in an African-European admixed population. PLoS Genet. 9 (2013) e1003372.
50. J.S. Allwood, S. Harbison, SNP model development for the prediction of eye colour in New Zealand, Forensic Sci. Int. Genet. 7 (2013) 444–452.

51. S. Walsh, L. Chaitanya, K. Breslin, C. Muralidharan, A. Bronikowska, et al., Global skin colour prediction from DNA. Hum Genet. 136 (2017) 847-863.
52. A. Fujimoto, M. Farooq, H. Fujikawa, A. Inoue, M. Ohyama, et al., A missense mutation within the helix initiation motif of the keratin K71 gene underlies autosomal dominant woolly hair/hypotrichosis, J. Invest. Dermatol. 132 (2012) 2342–2349.
53. Y. Shimomura, M. Wajid, L. Petukhova, M. Kurban, A.M. Christiano, Autosomaldominant woolly hair resulting from disruption of keratin 74 (KRT74), a potential determinant of human hair texture, Am. J. Hum. Genet. 86 (2010) 632–638.
54. J. Mengel-From, T. Wong, N. Morling, J. Rees, I. Jackson, Genetic determinants of hair and eye colours in the Scottish and Danish populations, BMC Genet. 10 (2009) 88.
55. Y. Shimomura, M. Wajid, Y. Ishii, L. Shapiro, L. Petukhova, et al., Disruption of P2RY5, an orphan G protein-coupled receptor, underlies autosomal recessive woolly hair, Nat. Genet. 40 (2008) 335–339.
56. J.M. de Gruijter, O. Lao, M. Vermeulen, Y. Xue, C. Woodwark, et al., Contrasting signals of positive selection in genes involved in human skin-color variation from tests based on SNP scans and resequencing. Investig Genet. 2 (2011) 24.
57. J. Söchtig, C. Phillips, O. Maroñas, A. Gómez-Tato, R. Cruz, et al., Exploration of SNP variants affecting hair colour prediction in Europeans. Int J Legal Med. 129 (2015) 963-75.
58. M. Edwards, A. Bigham, J. Tan, S. Li, A. Gozdzik, et al., Association of the OCA2 polymorphism His615Arg with melanin content in east Asian populations: further evidence of convergent evolution of skin pigmentation. PLoS Genet. 6 (2010) e1000867.
59. K.L. Hart, S.L. Kimura, V. Mushailov, Z.M. Budimlija, M. Prinz, et al., Improved eye- and skin-color prediction based on 8 SNPs, Croat. Med. J. 54 (2013) 248–256.
60. D.L. Duffy, G.W. Montgomery, W. Chen, Z.Z. Zhao, L. Le, et al., A three-single-nucleotide polymorphism haplotype in intron 1 of OCA2 explains most human eye-color variation. Am J Hum Genet. 80 (2007) 241-52.
61. R.A. Sturm, D.L. Duffy, Z.Z. Zhao, F.P. Leite, M.S. Stark, et al., A single SNP in an evolutionary conserved region within intron 86 of the HERC2 gene determines human blue-brown eye color, Am. J. Hum. Genet. 82 (2008) 424–431.
62. H. Eiberg, J. Troelsen, M. Nielsen, A. Mikkelsen, J. Mengel-From, et al., Blue eye color in humans may be caused by a perfectly associated founder mutation in a regulatory element located within the HERC2 gene inhibiting OCA2 expression, Hum. Genet. 123 (2008) 177–187.
63. M. Kayser, F. Liu, A.C.J.W. Janssens, F. Rivadeneira, O. Lao, et al., Three genome- wide association studies and a linkage analysis identify HERC2 as a human iris color gene, Am. J. Hum. Genet. 82 (2008) 422–423.
64. L. Chaitanya L, K. Breslin, S. Zuñiga, L. Wirken, E. Pośpiech, et al., The HIrisPlex-S system for eye, hair and skin colour prediction from DNA: Introduction and forensic developmental validation. Forensic Sci Int Genet. 35 (2018) 123-135.
65. F. Lona-Durazo, N. Hernandez-Pacheco, S. Fan, T. Zhang, J. Choi, et al., Meta-analysis of GWA studies provides new insights on the genetic architecture of skin pigmentation in recently admixed populations. BMC Genet. 20 (2019) 59.

66. A. Visconti, D.L. Duffy, F. Liu, G. Zhu, W. Wu, et al. Genome-wide association study in 176,678 Europeans reveals genetic loci for tanning response to sun exposure. Nat Commun. 9 (2018) 1684.
67. W. Branicki, U. Brudnik, T. Kupiec, P. Wolan´ ska-Nowak, A. Wojas-Pelc, Determination of phenotype associated SNPs in the MC1R gene, J. Forensic Sci. 52 (2007) 349–354.
68. M.H. Law, D.T. Bishop, J.E. Lee, M. Brossard, N.G. Martin, et al., Genome-wide meta-analysis identifies five new susceptibility loci for cutaneous malignant melanoma. Nat Genet.47 (2015) 987-995.
69. M. Kukla-Bartoszek, E. Pośpiech, A. Woźniak, M. Boroń, J. Karłowska-Pik, et al., DNA-based predictive models for the presence of freckles. Forensic Sci Int Genet. 42 (2019) 252-259.
70. A.M. Hillmer, F.F. Brockschmidt, S. Hanneken, S. Eigelshoven, M. Steffens, A. Flaquer, et al., Susceptibility variants for male-pattern baldness on chromosome 20p11. Nat Genet. 40 (2008) 1279–1281.
71. J.B. Richards, X. Yuan, F. Geller, D. Waterworth, V. Bataille, et al., Male pattern baldness susceptibility locus at 20p11. Nat Genet. 40 (2008) 40: 1282–1284.
72. L.C. Jacobs, M.A. Hamer, D.A. Gunn, J. Deelen, J.S. Lall, et al., A Genome-Wide Association Study Identifies the Skin Color Genes IRF4, MC1R, ASIP, and BNC2 Influencing Facial Pigmented Spots. J Invest Dermatol. 135 (2015) 1735-1742.
73. K.L. Hart, S.L. Kimura, V. Mushailov, Z.M. Budimlija, M. Prinz, et al., Improved eye- and skin-color prediction based on 8 SNPs, Croat. Med. J. 54 (2013) 248-256.
74. D.T. Bishop, F. Demenais, M.M. Iles, M. Harland, J.C. Taylor, et al., Genome-wide association study identifies three loci associated with melanoma risk. Nat Genet. 41 (2009) 920-5.
75. F.F. Brockschmidt, A.M. Hillmer, S. Eigelshoven, S. Hanneken, S. Heilmann, et al., Barth S, Fine mapping of the human AR/EDA2R locus in androgenetic alopecia. Br J Dermatol. 162 (2010) 899–903.
76. J.E. Cobb, S.G. Zaloumis, K.J. Scurrah, S.B. Harrap, J.A. Ellis, et al., Evidence for two independent functional variant for androgenetic alopecia around the androgen receptor gene. Exp Dermatol. 19 (2010) 1026–1028.
77. D.A. Prodi, N. Pirastu, G. Maninchedda, A. Sassu, A. Picciau A, et al. EDA2R is associated with androgenetic alopecia. J Invest Dermatol. 128 (2008) 2268–2270.

Supplementary Table 6 The results of mRMRe analysis for binary and 3-stage hair greying classification conducted in a 849-sample cohort

Rank	mRMRe analysis							
	Binary hair greying classification (greying vs. no greying)				3-stage hair greying classification (no greying vs. mild greying vs. severe greying)			
	SNP_ID	Chr position GRCh38	Gene/Locus	mRMRe Score	SNP_ID	Chr position GRCh38	Gene/Locus	mRMRe Score
**1**	**Age**	**-**	**-**	**0.2429273**	**Age**	**-**	**-**	**0.2555938**
**2**	**rs59733750**	**2:240780193**	***KIF1A***	**0.0026937**	**Sex**	**-**	**-**	**0.0071533**
**3**	**Sex**	**-**	**-**	**0.0026841**	**rs7680591**	**4:80276795**	***FGF5***	**0.0026000**
**4**	**rs68088846**	**21:34835870**	***RUNX1***	**0.0026483**	**rs59733750**	**2:240780193**	***KIF1A***	**0.0024376**
**5**	**rs1005241**	**22:47291868**	***TBC1D22A***	**0.0023004**	**rs10928235**	**2:144920547**	***TEX41***	**0.0024138**
**6**	**rs7680591**	**4:80276795**	***FGF5***	**0.0022084**	**rs68088846**	**21:34835870**	***RUNX1***	**0.0023223**
**7**	**rs2361506**	**2:233830694**	***MROH2A***	**0.0020803**	**rs2361506**	**2:233830694**	***MROH2A***	**0.0023167**
**8**	**rs12203592**	**6:396321**	***IRF4***	**0.0016857**	**rs45483393**	**9:89378809**	***SEMA4D***	**0.0020403**
**9**	**rs45483393**	**9:89378809**	***SEMA4D***	**0.0015962**	**rs12203592**	**6:396321**	***IRF4***	**0.0020377**
**10**	**rs2416699**	**9:119434462**	***BRINP1***	**0.0015622**	**rs1005241**	**22:47291868**	***TBC1D22A***	**0.0016952**
**11**	**rs164741**	**16:89625890**	***DPEP1***	**0.0014107**	**rs2416699**	**9:119434462**	***BRINP1***	**0.0016313**
**12**	**rs1683723**	**12:128415460**	***TMEM132C***	**0.0010814**	**rs2814331**	**10:86233584**	***GRID1***	**0.0009898**
**13**	**rs17143387**	**10:8272081**	***GATA3***	**0.0010660**	**rs164741**	**16:89625890**	***DPEP1***	**0.0008983**
14	rs1131471	1:152107513	*TCHH*	0.0007969	**rs1127228**	**16:27226789**	***NSMCE1***	**0.0005584**
15	rs1081073	5:158954504	*EBF1*	0.0007834	**rs17143387**	**10:8272081**	***GATA3***	**0.0003995**
16	rs2814331	10:86233584	*GRID1*	0.0007746	**rs29073**	**18:9971793**	***APCDD1***	**0.0003662**
17	rs62472724	7:143391804	*EPHA1*	0.0007642	rs1912702	11:79462038	*MIR708;TENM4*	0.0001052
18	rs2999852	1:163339512	*NUF2*	0.0007541	rs11621135	14:71192892	*PCNX*	0.0000844
19	rs1800209	13:77000848	*CLN5*	0.0005128	rs2306406	10:1015922	*GTPBP4*	0.0000623
20	rs310642	20:63530645	*PTK6*	0.0005033	rs34306906	4:121944043	*TRPC3*	0.0000511
21	rs4672907	2:218956447	*LOC391485*	0.0003816	rs36139924	7:259915	*FAM20C*	-0.0001330
22	rs71421546	2:176127129	*HOXD-AS2,HOXD3*	0.0000096	rs4656076	1:89013171	*GBP3*	-0.0001416
23	rs35780223	5:179083069	*ZNF354C*	0.0000036	rs10951154	7:27095695	*HOXA2*	-0.0001731
24	rs2227311	13:48412896	*P2RY5*	0.0000018	rs72811791	16:54245728	*LINC02169*	-0.0001805
25	rs1127228	16:27226789	*NSMCE1*	-0.0000688	rs13131900	4:82580733	*AC067942.2*	-0.0002247
26	rs1912702	11:79462038	*MIR708;TENM4*	-0.0000877	rs7009516	8:24351334	*ADAM28*	-0.0002249

27	rs10951154	7:27095695	*HOXA2*	-0.0001697	rs77995042	19:35508296	*DMKN*	-0.0002859
28	rs17752628	14:77732443	*SNW1*	-0.0001928	rs2379206	X:7077274	*HDHD1*	-0.0002955
29	rs2379206	X:7077274	*HDHD1*	-0.0002205	rs74393830	3:23134423	*RPL24P7*	-0.0002990
30	rs29073	18:9971793	*APCDD1*	-0.0002279	rs2507940	3:37494493	*ITGA9*	-0.0003308

Exome-wide candidate and literature-based selected SNPs were considered in analyses. The top 30 variants with the highest mRMRe score were extracted and pruned based on scree plots analysis. Variables marked with bold were included in the pruned list of predictors.

Supplementary Table 7 (The genotype-phenotype dataset generated with whole-exome sequencing) and **Supplementary Table 8** (The genotype-phenotype dataset generated with targeted next-generation sequencing) are in separate Excel files.
